# Trends of stillbirth, early neonatal and perinatal mortality rates and sociodemographic differentials of perinatal mortality rates in 17 low- and middle-income countries: A modeling study

**DOI:** 10.1371/journal.pmed.1004791

**Published:** 2026-08-24

**Authors:** Mohamed M. Ali, Luca Cegolon, Saverio Bellizzi

**Affiliations:** 1 The UNDP-UNFPA-UNICEF-WHO-World Bank Special Programme of Research, Development and Research Training in Human Reproduction, Department of Sexual and Reproductive Health and Research, WHO, Geneva, Switzerland; 2 Department of Medical, Surgical and Health Science, University of Trieste, Trieste, Italy; 3 Public Health Unit, University Health Agency Guliano-Isontina (ASUGI), Trieste, Italy; 4 United Nations University Institute for Water, Environment and Health (UNU-INWEH), Richmond Hill, Ontario, Canada; Washington University In St Louis: Washington University in St Louis, UNITED STATES OF AMERICA

## Abstract

**Background:**

Stillbirths and early neonatal deaths account for a substantial proportion of global child mortality. Reliable measurement of these outcomes remains challenging in settings where civil registration systems are incomplete and household surveys are the primary data source. However, survey-based estimates are often affected by underreporting and misclassification between stillbirths and early neonatal deaths. We aimed to apply a statistical adjustment approach to improve estimates of stillbirth, early neonatal, and perinatal mortality trends and to assess socioeconomic inequalities in countries with repeated Demographic and Health Surveys (DHS).

**Methods and findings:**

We analyzed 93 DHS surveys with reproductive calendar data from 17 LMICs conducted between 1990 and 2023, covering 1,019,253 pregnancies reported by 733,181 women. Pregnancies of at least 7 months’ gestation were included to align with the standard definition of stillbirth. Age-specific risks of stillbirth and early neonatal death were estimated using life-table methods and a modified Gompertz–Makeham (mGM) model that adjusts for common survey data quality issues, including underreporting, misclassification between stillbirths and deaths on days 0–1, and age-at-death heaping, i.e., the tendency to systematically misreport age of neonatal death often at 7 days at the expenses of neighboring days. Survey-specific estimates were used to evaluate trends over time and to examine associations with maternal and household characteristics through mGM models and the pooled etimates random-effects meta-regression; all covariates were used for adjusting the perinatal mortality rates and also taking the survey sampling design into consideration. A sensitivity analysis using alternate assumptions confirmed the robustness of the findings at country level.

Across countries, stillbirth, early neonatal, and perinatal mortality rates generally declined over time, although trends varied substantially between settings. The pooled annual decline was −0.60 deaths per 1,000 births for stillbirths (95% CI [−0.78, −0.41]), −0.50 for early neonatal mortality (95% CI [−0.66, −0.35]), and −1.08 for perinatal mortality (95% CI [−1.40, −0.76]). Lower perinatal mortality was associated with urban residence, higher maternal education, wealthier households, and higher national Human Development Index levels, whereas maternal age ≥30 years was associated with higher risk. Limitations include potential residual underreporting of pregnancy losses and lack of information on causes of death, in addition to our model assumptions relying on constant perinatal mortality metrics across different settings.

**Conclusions:**

Adjusting for survey data quality issues may potentially improve the use of household survey data for estimating stillbirth and early neonatal mortality. Although most countries experienced declines in perinatal mortality, progress remains uneven and significant socioeconomic disparities persist. Strengthening maternal and newborn health services and improving data systems will be essential to accelerate progress toward global targets for reducing perinatal mortality.

## Author summary

**Why was this study done?:**

- Stillbirths and early neonatal deaths remain a major global health challenge, with the vast majority occurring in low- and middle-income countries (LMICs).
- Many LMICs rely on household surveys such as the Demographic and Health Surveys (DHS) to estimate perinatal mortality because civil registration systems are incomplete.
- However, survey data often suffer from underreporting and misclassification of stillbirths and early neonatal deaths, limiting their usefulness for monitoring progress and designing interventions.

**What did the researchers do and find?:**

- We analyzed 93 DHS surveys from 17 LMICs, covering over a million pregnancies reported by more than 733,000 women across Africa, Asia, and Latin America.
- We applied a modified Gompertz–Makeham statistical model to adjust for common survey data quality issues, including underreporting, misclassification between stillbirths and early neonatal deaths.
- Overall, perinatal mortality declined in most countries, although progress varied substantially across settings.
- We assessed the association between sociodemographic variables and perinatal mortality, which revealed a significant reduction in the risks of death for those living in urban settings and affluent households

**What do these findings mean?:**

- Adjusting for survey data quality may improve estimates of stillbirth and early neonatal mortality derived from household surveys.
- Despite overall declines, large disparities remain between and within countries, particularly affecting rural and poorer populations.
- Applying robust statistical adjustment methods to household survey data with noticeable data quality issues can provide valuable evidence for monitoring progress toward global maternal and newborn health goals.
- Using fixed adjustment factors to adjust data quality in the proposed modeling framework may be a limitation.

## Abstract 3

Using data from demographic surveys, Mohamed Ali and colleagues applied statistical adjustments to improve estimates of stillbirth, early neonatal, and perinatal mortality trends for 17 low- and middle-income countries. Their analysis suggests that mortality rates have generally declined since 1990, but progress has varied across countries.

## Introduction

Perinatal mortality refers to the death of a fetus at 28 weeks of gestation or later (stillbirth) [1] or the death of a newborn infant within the first seven days of life (early neonatal mortality) [2]. More than 50 million stillbirths have occurred since 2000, with about 2 million stillbirths in 2023, the most recent year of estimation [3]; similarly, there were 2.3 (2.2–2.6) million deaths in the neonatal period in 2022 alone, with the biggest burden in low- and lower-middle-income countries (LMIC) [4]. Specifically, 98% of globally reported neonatal deaths and 97% of stillbirths occur in LMIC [5].

Despite the global relevance of stillbirth and early neonatal mortality, survey data have low quality that need advanced statistical tools to be used to produce sound evidence. In particular, the analysis of trends and differentials are rarely undertaken because of these issues. In this regard, some efforts have been ongoing in accurately describing variations in both the level and the shape of under-5 mortality across a variety of contexts. However, these are based on databases containing information by detailed age in countries with high-quality vital registration systems [6].

To design appropriate interventions, policy makers and program managers need information on population groups at high risk of stillbirth and early neonatal mortality. In addition, to assess how countries are making progress in achieving Sustainable Development Goals (SDGs) and in reducing the burden of stillbirths and early neonatal deaths, countries need data over time to assess the trends.

The Demographic and Health Survey (DHS) program plays a central role in generating nationally representative data on maternal and child health in low- and middle-income countries and is a key source for global perinatal and stillbirth mortality estimates, including those produced by the United Nations Inter-Agency Group for Child Mortality Estimation (UN IGME) [3]. DHS birth histories and reproductive calendars enable standardized cross-country comparisons over time. However, estimates are subject to limitations, including recall bias (especially for events occurring several years before the survey), misclassification between stillbirths and very early neonatal deaths, and underreporting due to stigma or cultural factors. These data quality issues have greatly limited the usefulness of such data and a recent joint assessment of stillbirth and early neonatal mortality data, proposed by Ali and colleagues [7], aimed at enhancing the use of data in surveys by proposing data quality metrics and exploring adjustment procedures to obtain the best possible measure of perinatal mortality.

Specifically, a flexible statistical Gompertz–Makeham (mGM) model was developed to adjust for data quality collected by the DHS in measuring the stillbirth and the early neonatal mortality collected within the same survey by adjusting for possible omission, transference, and under-reporting, as well as smoothing; the model was established checking such indicators against figures from more reliable sources and proposing cut-offs for adjustment. The mGM adjusted the observed data by correcting implausible reporting patterns, including under-reported stillbirths, under-reported deaths on days 0–1, and age heaping at day 7. These corrections reallocate deaths to their most likely timing and outcome, resulting in higher estimated stillbirth and perinatal mortality rates than those obtained directly from the survey data. The application of the proposed adjustment to the study sample of 157 DHS led to median stillbirth rates increased by (110%) from 12.2 to 25.6 per 1,000 births, and to perinatal mortality rate increasing by (73%) from 32.6 to 44.8 per 1,000 births [7].

The analysis indicated that stillbirth and age-specific neonatal mortality data from household surveys can be more effectively utilized by incorporating data quality metrics and accounting for under-reporting and misclassification of deaths. By examining stillbirths alongside early neonatal mortality, it becomes possible to thoroughly evaluate the impact of incomplete or inaccurate reporting in surveys. This approach supports countries in maximizing the value of household survey data for planning and monitoring strategies aimed at reducing late fetal loss and early neonatal mortality.

We aimed to apply the proposed adjustment methodology to estimate trends in stillbirth, early neonatal, and perinatal mortality rates, and to assess inequalities in perinatal mortality for the 17 LMICs with at least four standard publicly available DHS surveys with reproductive calendar, thereby improving the use of survey-based data in settings where death registration systems are inadequate.

## Materials and methods

### Data source

DHS is the major data source for pregnancy outcomes worldwide. From 1984 to date, the DHS programme had eight phases (DHS-I to DHS-VIII) collecting data from more than 400 surveys in more than 90 countries [8]. Throughout all DHS phases, the model questionnaire included Full Birth History (FBH), capturing a woman’s lifetime live births and survival status, which most countries have implemented to calculate early childhood mortality. FBH has undergone minor changes during the last three decades. Stillbirths were initially not captured or reported in DHS-I. In DHS-II to DHS-VIII, reproductive calendars were used to collect data on pregnancy outcomes including abortion and stillbirth. Since DHS-III, stillbirth rates have been shown in the standard national DHS tabulation. DHS-VII introduced a reverse truncated history for non-live births, and in the most recent DHS round (VIII), the full birth history module is replaced by a full pregnancy history module.

The reproductive calendar takes the form of a grid, where interviewers are trained to enter on the monthly grid all live births (and current pregnancy if any), ascertained earlier in the interview using the FBH module. Any pregnancy loss detected at this stage of detailed questioning is entered into the calendar, but no attempt is made to distinguish induced from spontaneous abortions. The exact length of the period covered by the calendar varies depending on the defined recall period and duration of the survey data collection. In most surveys, the period covered by the calendar includes the months up to the month of interview in the year of interview, plus the five calendar years preceding the year of interview. The reproductive calendar records the current pregnancy and the pregnancy loss in months and the FBH module records the child death in days, months or years, and current age in months, therefore the exact gestational age at pregnancy loss or age at death or current age are not reported.

This analysis is based on the publicly available 93 DHS surveys with reproductive calendar from 17 low- and middle-income countries with at least four standard DHS surveys. Country-specific surveys included 51 from sub-Saharan Africa (Ghana 4, Kenya 5, Malawi 5, Mali 4, Nigeria 4, Rwanda, 5, Senegal 7, Tanzania 4, Uganda 4, Zambia 4, and Zimbabwe 5), 12 from North Africa and Western Asia (Egypt 6, and Jordan 6), 16 from Central, South, and Southeast Asia (Bangladesh, 9 and Indonesia 7), and 14 from Latin America and the Caribbean (Colombia 5, and Peru 9) S1 Table. We excluded Armenia’s four surveys (2000–2016), Colombia 1990 survey and Jordan 2023 by following the same inclusion criteria in *Ali* and colleagues in retaining surveys with a minimum of 25 stillbirths or deaths on day zero and one (S2 Table). We further excluded the two interim surveys: Egypt Interim DHS 2003 and Jordan Interim DHS 2009.

We extracted the gestational age (in months) and pregnancy outcomes (birth, termination, currently pregnant) from the calendar. Pregnancies terminated before 7 months’ gestation were excluded, in line with WHO’s definition of stillbirths (A baby who dies after 28 weeks of pregnancy, approximately 7 months, but before or during birth) and perinatal mortality [1]. Livebirths reported in the calendar were linked with the corresponding livebirths (including multiple births) reported in the birth history, using Century Month Code of birth to obtain the survival status, current age, and age at death (day, month, and year). Current pregnancies of seven or more months were also included, as censored on the survey month. We retained data for pregnancies that were conceived between 7 and 66 months before the survey date. Livebirths that occurred in the month of the interview (age zero-month) were randomly imputed to be between 10 and 27 days old.

In addition, we extracted covariates based on women’s characteristics and grouped them where relevant, household wealth tertiles (first tertile = poor, second tertile = middle, and third tertile = rich), urban-rural residence, mother’s level of education (No education, primary, and secondary plus), age of the respondent at conception of the index pregnancy in years (<25, 25–29, and ≥30 years), and number of living children at conception of the index pregnancy (0–1, and 2+). We compute the three data quality indicators, Stillbirth to D0–1 ratio, D0–1 to D2–6 ratio, and heaping index at day 7, calculated as five times the number of deaths reported on day 7 divided by the sum of all deaths on days 5–9 (S3 Table).

### The methods

Because stillbirths and the current age of living children are reported in months, whereas the age at death of neonates is reported in days, the data were considered interval-censored, the pregnancy-based file were restructured to create an epoch-based file to compute conditional probabilities of death; and fit models for interval-censored data. Age-specific risks of death and the cumulative probabilities of death from 7-month gestation to day 10 were computed using life-table methods for real cohorts. Then the underlying risks of stillbirth and daily deaths were modeled applying mGM model on each survey.

If *p*_*ij*_ is the probability of pregnancy loss or child death reported for the *i*th pregnancy/child, where *i* = 1, 2,  .  .  ., *n*, at *j*th time interval, *j* = 1, 2,  .  .  ., *k*. Also let π_*ij*_ be the probability that the *i*th of *n* pregnancies/children is still alive at time interval *t*_*j−1*_ and has experienced the loss or death in the *j*th time interval, *j* = 1, 2,  .  .  ., *k*. This is, therefore, the conditional probability of pregnancy loss or child death in the *j*th time interval, given that loss/death occurs after *t*_*j−1*_.

Using *T*_*i*_ to denote the random variable associated with the event time of the *i*th of pregnancy/child, we therefore have


$$\begin{array}{c} p_{ij}=P(t_{j-\text{1}}\leq T_{i}<t_{j}) \end{array}$$

and


$$\begin{array}{c} \pi_{ij}=P(t_{j-\text{1}}\leq T_{i}<t_{j}|T_{i}\geq t_{j-\text{1}}) \end{array}$$

For *j* = 1, 2,  .  .  ., *k*


$$\begin{array}{c} {\text{1}-\pi }_{ij}=P\left(T_{i}\geq t_{j}|T_{i}\geq t_{j-\text{1}}\right)=\frac{S_{i}(t_{j)}}{S_{i}\left(t_{j-\text{1}}\right)} \end{array}$$

Adopting a proportional hazards model for the event times, the hazard of loss or death being reported at time *t*_*j*_ for the *i*th pregnancy/child can be expressed


$$\begin{array}{c} h_{i}\left(t_{j}\right)=\text{exp}(\eta_{i})h_{\text{0}}(t_{j}) \end{array}$$

$$\begin{array}{c} {\text{1}-\pi }_{ij}={\left[\frac{S_{\text{0}}(t_{j})}{S_{\text{0}}(t_{j-\text{1}})}\right]}^{\text{exp}(\eta_{i})} \end{array}$$

$$\begin{array}{c} og\left\{-\text{log}(\text{1}-\pi_{ij})\right\}=\eta_{i}+log\left[-log\left\{S_{\text{0}}(t_{j})/S_{\text{0}}(t_{j-\text{1}})\right\}\right] \end{array}$$

$$\begin{array}{c} =\eta_{i}+\gamma_{j} \end{array}$$

$$\begin{array}{c} =\beta X+\gamma_{\text{1}}+\gamma_{\text{2}}+\gamma_{\text{3}}+\ldots +\gamma_{k} \end{array}$$

This is a piecewise exponential linear model for the complementary log-log transformation of π_*ij*_, in which the parameters γ_*j*_, *j* = 1, 2,  .  .  ., *k*, are associated with the *j* time intervals. Where $\gamma_{j}\;=\gamma_{\text{1}}+\gamma_{\text{2}}+\gamma_{\text{3}}+\ldots +\gamma_{k}$ is the time interval (age in months and days), specifically *γ*_1_ = month 7–9, *γ*_2_ = death on day 0 and 1, and *γ*_3_ = death on day 2, etc. and $\eta_{i}={BX}_{i}\;$ is the linear predictors.

Then the mGM model parametrization is


$$\begin{array}{c} og\left\{-\text{log}(\text{1}-\pi_{ij})\right\}=\beta X+\gamma_{\text{1}}+\gamma_{\text{2}}+\gamma_{j},\;where\;j\geq \text{3} \end{array}$$

where exp(γ_1_) is the risk of stillbirth, exp(γ_2_) is the risk of death on day zero and one (D0-1, defined as deaths within the first 24 h of life is classified as first-day deaths which we refer to as day 0, and those occurring 24–47 h after birth as second-day deaths (i.e., day 1*)*, and exp(γ_j_) is the risk of death on day 2, 3, 4, etc. To account for underreporting and transference between stillbirth to death on D0-1 in both directions, the mGM model with no intercept included a constrain equation for the first two coefficients stillbirth and death D0-1 to be [$\gamma_{\text{1}}-\gamma_{\text{2}}=\text{log}(\text{1}.\text{8}\text{9})]$ if the observed ratio is below the median value of 1.89. If the observed ratio of deaths on D0-1 to deaths on day two to six (D2-6, refers to deaths occurring between day three and day seven of life) is lower than 2.4, then deaths on D0-1 were weighted by 2.40 divided by the observed ratio, and weighted by 1.89 to simultaneously adjust for underreporting and transference, and the linear functional form of γ_*j*_ (*j* ≥ 3) smooths the heaping on day 7.

Prior to fitting the mGM model, the correlations between the unadjusted survey-specific stillbirth and early neonatal mortality rates, generated from the lifetable (the piece-wise model) and the three data quality indicators (Stillbirths to D0–1 ratio, D0–1 to D2–6 ratio, and heaping index at day 7) were assessed, overall and disaggregated by the above covariates using the Pearson correlation coefficient (ecological correlation).

The survey-specific rates of stillbirth, and early neonatal from the mGM models was used to measure trends over time, by fitting country-specific linear models that include the calendar mid-point. The survey-specific full mGM models that also include the above selected covariates were fitted for perinatal mortality only. The random-effects meta-regression models were used to estimate the pooled effects (in terms of adjusted rate ratios) of each covariate on perinatal mortality, generated from the full mGM models. Correlations of the survey-specific Human Development Index (HDI) at the time of each survey with stillbirth, early neonatal and perinatal mortality were plotted. In all analyses, the survey’s sampling design, namely the geographical clusters and the design weights were taken into consideration using the survey commands, and all analyses were conducted in Stata (version 19.0).

### Sensitivity analysis

To assess the robustness of the findings, we conducted a sensitivity analysis using alternative adjustment assumptions. Instead of applying the fixed thresholds used in the primary model, we adopted plausible ranges reported by Ali and colleagues for the stillbirth-to-D0–1 and D0–1-to-D2–6 ratios [7]. Ratios within the plausible ranges were retained, whereas values outside these ranges were adjusted to the reference values.

The country-specific stillbirth, early neonatal and perinatal mortality rates with their 95%CIs that are based on the original and the alternative assumptions are depicted in (S1 Fig). The median and the lower quartile differences between the model-based rates and the alternative adjustment are zero (S2 Fig).

## Results

This study was based on the publicly available 93 DHS surveys with reproductive calendar from 17 low- and middle-income countries with at least four standard DHS surveys. Specifically, the analysis included data from four regions: sub-Saharan Africa (51 surveys from 11 countries), North Africa and Western Asia (12 surveys from 2 countries), South and Southeast Asia (16 surveys from 2 countries), and Latin America and the Caribbean (14 surveys from 2 countries).

The total number of pregnancies extracted from all surveys for this study are 1,019,253, contributed by 733,181 women with an average number of 1.40 pregnancies per woman. The number of pregnancies ranged from 3,074 in Ghana 2008 to 34,904 in Nigeria 2018 (S1 Table). The number of stillbirths ranged from 39 in Ghana 2003–571 in Nigeria 2018; the number of livebirths ranged from 2,921 in Ghana 2008–32,887 in Nigeria 2018 (S4 Table). The heaping index (the tendency to systematically misreport age of neonatal death often at 7 days at the expenses of neighboring days) and the D0-1 (defined as deaths within the first 24 h of life is classified as first-day deaths which we refer to as day 0, and those occurring 24–47 h after birth as second-day deaths (i.e., day 1)/D2-6 (Similarly, D2-6 refers to deaths occurring between day three and day seven of life) disaggregated by covariates were not correlated with lifetable generated stillbirth nor with early neonatal mortality rates, and as expected, the stillbirth/D0-1 ratio strongly correlated with stillbirth and early neonatal mortality, although with few exceptions (S5 Table), which do not require disaggregation of the data quality in the model fitting.

Table 1 shows the adjusted stillbirth, early neonatal and perinatal mortality rates per 1,000 births for the first survey round and the last survey round in each country. Stillbirth was highest in Bangladesh 55.7 per 1,000 births and then dropped by 58% to 23.3 per 1,000 births and was lowest in Ugandan 21.9 per 1,000 births and only dropped by just 7% to 20.4. Early neonatal mortality was highest in Bangladesh 43.7 per 1,000 births and then dropped by 59% to 17.9 per 1,000 births and was lowest in Colombia 17.3 per 1,000 births then dropped by 75% to 4.3 per 1,000 births. The rates in Zimbabwe in the first and last surveys were the same. Perinatal mortality. Perinatal mortality was highest in Bangladesh 97.0 per 1,000 births then dropped by 58% to 40.8 per 1,000 births and lowest in Colombia 39.9 per 1,000 births then dropped by 70% to12.2 per 1,000 births. In 13 countries, the reductions in perinatal mortality were contributed by the reduction in stillbirth with the exception of Kenya, Mali, Uganda and Indonesia. The country-specific trends in stillbirth, early neonatal and perinatal mortality over time were depicted on S3 Fig. Although there is regular declining patterns in the three rates, however, there is noticeable erratic declining patterns in some countries.

**Table 1:** Adjusted stillbirth, early neonatal and perinatal mortality rates, per 1,000 births, by first and last survey for each country.

Region	Country	Survey year	Stillbirth rate	95%CI	Early neonatal mortality rate	95%CI	Perinatal mortality rate	95%CI
**Sub-Saharan Africa**								
	Ghana							
		2003	43.8	[35.0,54.9]	36.1	[28.0, 46.6]	78.3	[62.0, 98.9]
		2022/23	20.6	[16.9,25.0]	14.4	[11.4, 18.3]	34.7	[28.1, 42.9]
	Kenya							
		1998	24.9	[19.8,31.5]	19.4	[14.8, 25.7]	43.9	[34.2, 56.4]
		2022	25.2	21.0,30.2]	18.5	[14.7, 23.5]	43.3	[35.4, 53.0]
	Malawi							
		2000	40.1	[34.8,46.4]	33.1	[28.1, 39.1]	72.0	[61.9, 83.7]
		2024	19.2	[16.8,22.0]	15.6	[12.9, 18.9]	34.5	[29.5, 40.5]
	Mali							
		2001	45.5	[38.7,53.5]	37.7	[31.9, 44.4]	81.5	[69.4, 95.5]
		2018	37.9	[32.3,44.5]	29.8	[24.5, 36.4]	66.6	[56.0, 79.3]
	Nigeria							
		2008	35.1	[31.9,38.6]	29.0	[26.2, 32.2]	63.1	[57.3, 69.6]
		2023/24	27.1	[24.8,29.5]	23.2	[20.8, 26.0]	49.7	[45.1, 54.7]
	Rwanda							
		2000	39.3	[34.0,45.4]	31.1	[26.3, 36.8]	69.2	[59.4, 80.5]
		2019/20	20.8	[17.5,24.7]	14.8	[11.8, 18.7]	35.3	[29.0, 43.0]
	Senegal							
		2005	43.8	[37.5,51.3]	33.5	[28.2, 39.8]	75.9	[64.6, 89.1]
		2023	26.1	[21.7,31.5]	21.5	[17.2, 26.7]	47.0	[38.5, 57.4]
	Tanzania							
		2004/5	22.8	[19.6,26.6]	18.7	[15.2, 23.1]	41.1	[34.5, 49.1]
		2022	20.7	[17.8,24.1]	16.9	[13.8, 20.7]	37.2	[31.3, 44.3]
	Uganda							
		2000/1	21.9	[18.6,25.7]	19.0	[15.3, 23.6]	40.4	[33.6, 48.7]
		2016	20.4	[18.3,22.7]	16.9	[14.4, 19.9]	36.9	[32.4, 42.1]
	Zambia							
		2007	34.0	[28.2,41.0]	26.5	[21.4, 32.9]	59.6	[49.0, 72.6]
		2024	23.3	[20.0,27.3]	16.2	[13.3, 19.9]	39.2	[33.0, 46.6]
	Zimbabwe							
		1994	28.8	[22.2,37.5]	21.8	[16.0, 29.8]	50.0	[37.8, 66.1]
		2015	26.9	[21.9,33.1]	21.9	[17.0, 28.2]	48.2	[38.5, 60.4]
**North Africa Western Asia**								
	Egypt							
		1992/93	37.1	[30.8,44.7]	31.3	[25.6, 38.3]	67.2	[55.6, 81.3]
		2014	16.1	[12.9,20.1]	12.7	[9.9, 16.3]	28.6	[22.7, 36.0]
	Jordan							
		1990	25.0	[20.3,30.8]	19.6	[15.5, 24.8]	44.1	[35.5, 54.8]
		2017/18	16.5	[11.6,23.5]	13.7	[9.3, 20.1]	30.0	[20.8, 43.1]
**South & Southeast Asia**								
	Bangladesh							
		1993/94	55.7	[48.1,64.5]	43.7	[37.0, 51.6]	97.0	[83.3, 112.8]
		2022	23.3	[20.2,26.9]	17.9	[14.7, 21.9]	40.8	[34.6, 48.2]
	Indonesia							
		1991	23.0	[19.3,27.4]	20.5	[16.6, 25.4]	43.1	[35.6, 52.2]
		2017	17.6	[15.2,20.3]	13.1	[11.0, 15.8]	30.5	[26.0, 35.8]
**Latin America & Caribbean**								
	Colombia							
		1995	23.0	[17.6,30.1]	17.3	[12.9, 23.4]	39.9	[30.2, 52.8]
		2015/16	7.9	[6.0,10.4]	4.3	[3.1, 6.1]	12.2	[9.1, 16.5]
	Peru							
		1991/92	23.8	[20.1,28.1]	18.8	[15.5, 22.9]	42.1	[35.3, 50.3]
		2012	10.1	[8.0,12.9]	7.5	[5.5, 10.6]	17.6	[13.4, 23.3]

**Fig 1:**
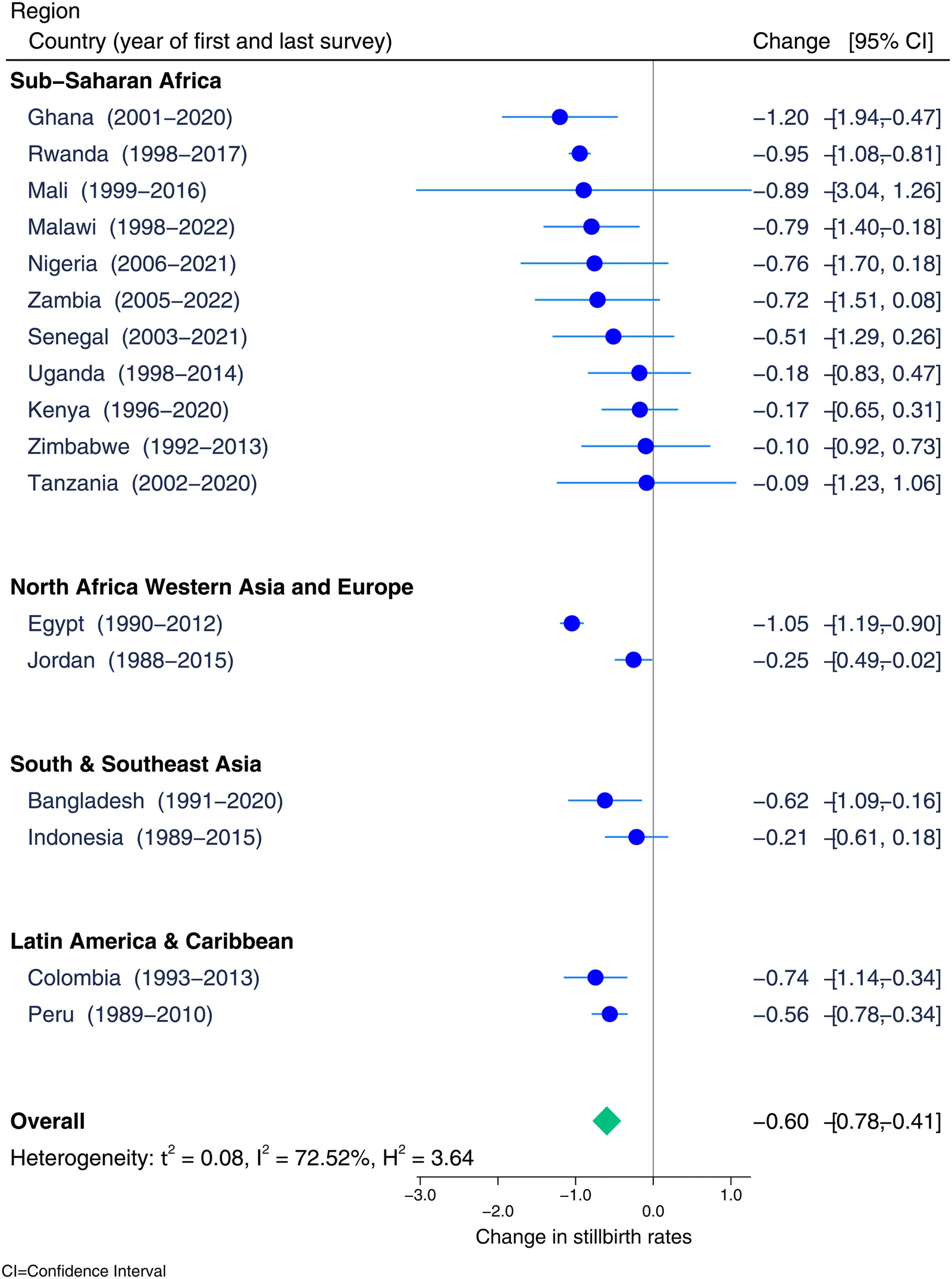
Annual declines in stillbirth rates, by country.

**Fig 2:**
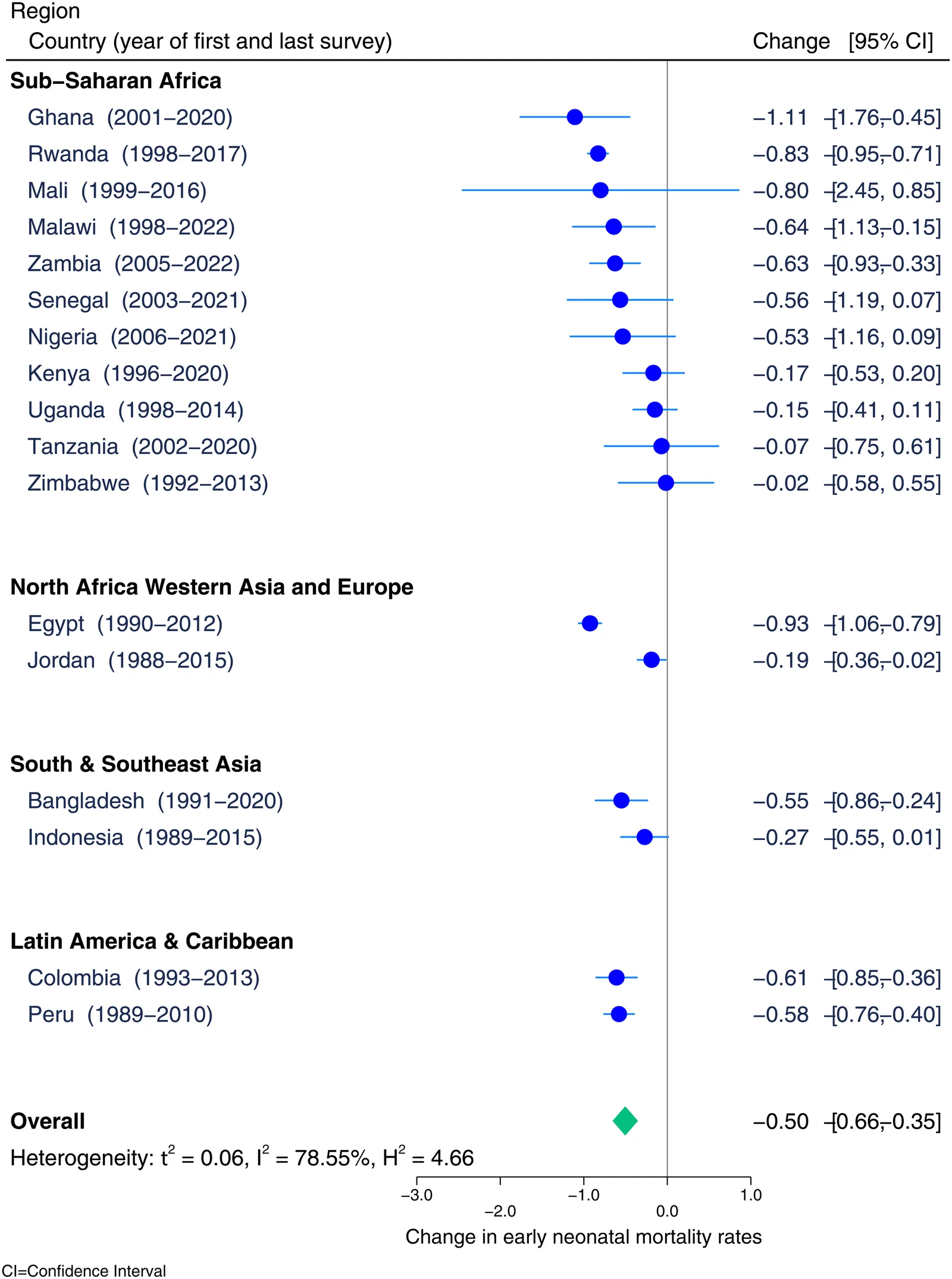
Annual declines in early neonatal mortality rates, by country.

**Fig 3:**
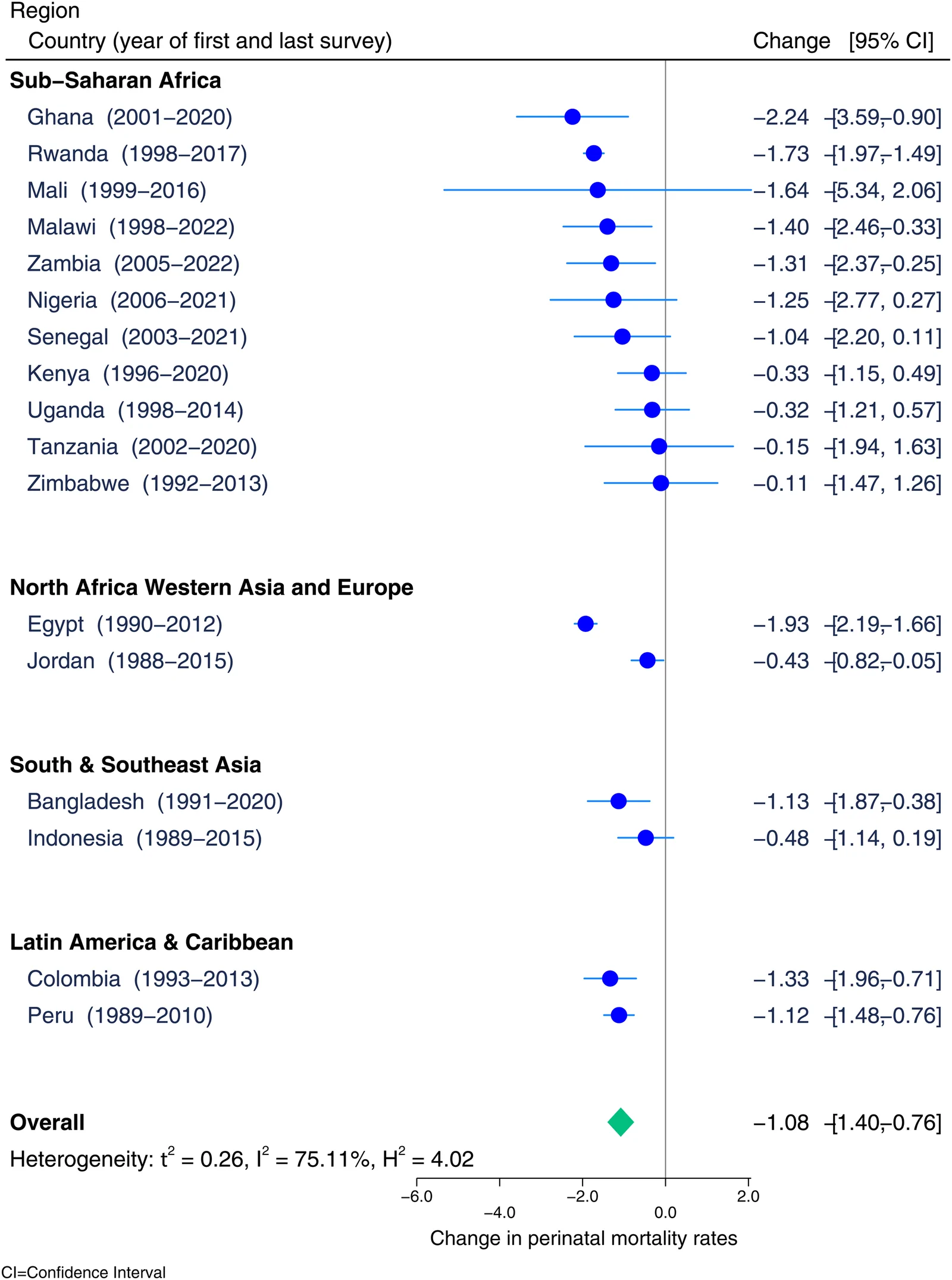
Annual declines in perinatal mortality rates, by country.

The pooled annual rates of decline in stillbirth, early neonatal and perinatal mortality rates are depicted in Figs 1–3. The pooled annual decline rate in stillbirth is −0.60 (95%CI [−0.78,-0.41]), and the decline was significant in eight out of 17 countries. The pooled annual decline of early neonatal mortality rate is −0.50 (95%CI [−0.66, −0.35]), and the decline was significant in nine countries. The pooled annual decline of perinatal mortality rate is −1.08 (95%CI [−1.40, −0.76]), and the decline was significant in nine countries. For the three rates, the largest annual declines were observed in Ghana and the smallest declines in Zimbabwe.

The annual rate ratios of perinatal mortality adjusting for the selected covariates are summarized in Table 2 and depicted in S4 Fig. The rate ratios of perinatal mortality adjusted for data quality indicators decreased for mothers in urban settings compared to their rural counterpart, with mothers’ level of education, with household wealth, and for mothers with two or more live births, however, the rate ratios significantly increased with maternal age. The within covariates increase or decrease in rate ratios are in the expected direction and dose–response trend. The scatter plots of stillbirths, early neonatal and perinatal mortality rates against the HDI (S5 Fig), respectively, showed statistically significant correlations between increase in HDI and lower stillbirth, early neonatal and perinatal mortality rates.

**Table 2:** Pooled adjusted rate ratios (aRRs) of perinatal mortality from all 17 countries, using a modified Gompertz–Makeham (mGM) model, for the selected covariates.

Covariates	aRR	95% CIs
Place of residence		
Rural	1.00	
Urban	0.78	[0.73, 0.82]
Level of education		
No education	1.00	
Primary	0.80	[0.70, 0.90]
Secondary+	0.62	[0.53, 0.72]
Household wealth tertile		
Poor(first tertile)	1.00	
Middle (second tertile)	0.86	[0.81, 0.91]
Rich (third tertile)	0.65	[0.57, 0.73]
Maternal age group		
15–24	1.00	
25–34	1.16	[1.06, 1.26]
35+	1.78	[1.64, 1.92]
No of living children		
0–1	1.00	
2+	0.39	[0.36, 0.43]

aRR is the adjusted rate ratios adjusted from place of residence; level of education; household wealth; maternal age; and number of living children.

The sensitivity analysis conducted to assess the robustness of the findings showed the same temporal dynamics, with heavy overlaps of the 95% confidence intervals, highlighting no statistically significant differences between the primary model and the sensitivity test.

## Discussion

We explored the trends of stillbirth, early neonatal and perinatal mortality rates for 17 countries across four regions using a flexible statistical model that adjusts for data quality when measuring stillbirth and early neonatal mortality. Our analysis revealed a steady decline on stillbirth, early neonatal and prenatal mortality rates in most countries. However, trend patterns were highly variable across countries, a variation that was also evident in the annual changes. Specifically, in stillbirth and early neonatal mortality rates declined drastically and consistently for countries in Latin America and North Africa. For example, Egypt and Peru exhibited sharp initial declines in both stillbirth and early neonatal mortality (and the combined perinatal mortality), which later plateaued. These patterns align with global data showing East Asia and Latin America achieving the largest stillbirth reductions between 2000 and 2019, with annual reduction rates exceeding 3% [9]. In contrast, countries in South Asia such as Bangladesh, and approximately one-third of countries in sub-Saharan Africa, including Kenya, showed either flat or inconsistent trends in stillbirth reduction. This regional divergence mirrors global findings, where progress in sub-Saharan Africa has remained stubbornly slow, with some countries even experiencing stagnation or reversals.

Annual changes were generally more pronounced for early neonatal mortality than for stillbirths, which affected the overall perinatal mortality. In several countries, such as Malawi, Bangladesh, and Tanzania, the trends in early neonatal mortality showed a certain improvement, even when stillbirth rates remained stagnant. This may reflect recent efforts to improve intrapartum and neonatal care, which may not yet be reflected in stillbirth data due to persistent issues such as poor antenatal care (ANC) or delays in accessing facility-based deliveries.

Countries like Malawi, Rwanda, Egypt, Colombia, Peru, and Ghana demonstrated accelerated declines in stillbirth and early neonatal mortality in the early years of the study period, followed by more modest progress. Such trends might be attributed to increased coverage along the continuum of care, particularly in public health services and among lower-income and rural populations [10]. In Malawi, for instance, the facility delivery rate rose sharply from 53% in 2000 to 90% in 2014, driven in large part by a 2007 ban on childbirths assisted by traditional birth attendants [11]. Similarly, Rwanda implemented a health system strengthening initiative that combined clinical mentoring with improved facility readiness, which significantly enhanced the quality of newborn care and contributed to reduction in neonatal mortality [12].

In contrast, countries such as Jordan, Indonesia, and Bangladesh displayed limited improvement across the study period. In Jordan, neonatal mortality has remained stagnant since 2012, with a significant share of neonatal deaths deemed preventable [13]. Researchers have called for enhanced death registration systems, better documentation of causes of mortality, and stronger antenatal care services. In Bangladesh stagnated post-2000, largely due to disparities in quality and readiness between public and private maternity sectors [14]. The country has experienced low levels of institutional births (53%), which has limited the population-level impact of interventions such as chlorhexidine cord care.

Tanzania presents a mixed picture. Our analysis indicates a certain stagnation in perinatal mortality from 2006 to 2013, which confirms the findings from other sources such as hospital-based retrospective surveys [15]. Senegal, uniquely, showed a divergence between stillbirth rates (which slightly increased), and early neonatal mortality (which decreased), leading to a slightly decreased perinatal mortality. This may point to inconsistent care across the maternal-newborn continuum, where quality childbirth services fail to compensate for gaps in antenatal care, leading to preventable stillbirths due to causes like malaria, sepsis, and other infections [16].

Zimbabwe’s trend is particularly concerning. Since the early 2000′ both stillbirth rates and perinatal mortality rates have increased, despite high levels of antenatal care coverage (93% with ≥1 visit and 74% with ≥4 visits), institutional deliveries (88%) [17], of prevention of mother-to-child transmission (PMTCT) participation (>90% in 2018) [18]. These troubling trends reflect broader systemic challenges [19], including chronic economic stress and high HIV prevalence, which have eroded the quality of maternal and newborn care.

Nigeria continues to face a very high burden of perinatal mortality and stillbirth compared with global averages. Between 2000 and 2019, the total number of stillbirths in Nigeria increased by about 15%, and in 2019 the country had an estimated 171,428 stillbirths, placing it among the highest globally and in Africa [20]. Our analysis confirms that Nigeria’s stillbirth rate has remained high and far above targets such as ≤12 per 1,000 total births, despite some slow declines in national estimates. Early neonatal mortality rates, and overall perinatal mortality, have also stayed unacceptably high, reflecting both intrapartum stillbirths and early neonatal deaths as ongoing challenges [21].

Our findings on urban-rural disparities confirm that urban settings are associated with lower perinatal mortality rates. This aligns with existing evidence showing that women in urban areas in sub-Saharan Africa have 54% higher odds of receiving ANC compared to rural women [22]. ANC4+ coverage ranges from 32% to 92% across urban-rural divides [23], indicating that location remains a major determinant of service access and quality. In terms of education, our analysis did not reveal a significant relationship between maternal education and stillbirth rates, which contrasts with findings from a multi-country cross-sectional study indicating that low maternal education (OR: 1.50, 95% CI [1.01,2.24]) is significantly associated with increased stillbirth risk [24]. However, we found stronger alignment with wealth status, where higher financial security correlated with reduced stillbirth and early neonatal mortality. Previous studies have consistently shown that infants born to mothers with lower educational attainment or living in rural settings experience higher risks of early neonatal mortality, largely due to disparities in health literacy, care-seeking behavior, and access to skilled obstetric and neonatal services [25,26]. In contrast, stillbirths are often more strongly linked to biological and obstetric conditions, including placental dysfunction, hypertensive disorders of pregnancy, infections, fetal growth restriction, and congenital anomalies [27]. Global analyses highlight that many stillbirths occur due to medical complications during pregnancy or labor rather than postnatal care practices [27]. Consequently, socioeconomic determinants may show stronger statistical associations with early neonatal mortality than with stillbirth, as the former is more directly influenced by care-seeking behaviors and access to neonatal health services.

The patterns we identified may be best interpreted through the lens of the maternal mortality transition framework, which links mortality trends to broader eco-social forces. In low HDI contexts, high mortality is often driven by systemic challenges such as political instability, weak health systems, and social inequities. In contrast, higher-HDI countries tend to experience declining mortality because of stronger institutions, better governance, and effective social development programs [28]. Our findings indicate that stillbirth and perinatal mortality are subject to the same underlying dynamics.

By mapping country-specific trends, we offer a nuanced view that challenges any notion of one-size-fits-all solution. For example, while both Bangladesh and Nepal introduced chlorhexidine cord-care in homes and facilities, population-level coverage differed substantially due to disparity in institutional births rates, 78% in Nepal versus 53% in Bangladesh, leading to higher effective coverage in Nepal (61% versus 56%) [29]. Similarly, in some countries like Senegal, even a cesarean section rate of 5% among the poorest remains unmet exacerbating stillbirth risks.

At the current rate of progress, 64 countries, primarily in sub-Saharan Africa and South Asia, are off-track to meet the newborn mortality reduction targets in the UN Sustainable Development Goals by 2030.^4^ Although the global decline in perinatal mortality was promising between 1990 and 2015, progress has since slowed significantly. Reaching the most disadvantaged populations, particularly in rural and underserved communities, will be critical.

Our study had several limitations. Fetal sex was not recorded, and ethnicity data were inconsistent. Another limitation is that we do not have data on whether the stillbirth was antepartum or intrapartum, or cause of neonatal death. Severe under-reporting of stillbirths in surveys remained a key data quality issue, and although model-based adjustments improved mortality estimates, some surveys still showed lower-than-expected stillbirth rates with wide uncertainty. This seems, for instance, the case for Tanzania and Kenya, where probably trends are not completely reliable due to earlier survey figures being too low. Furthermore, as per the original study by Ali and colleagues, we assumed quality metrics were constant to simplify analyses, which is line also with the observation of Perin and colleagues on assuming a constant cause of mortality distribution across the neonatal period [30]; in addition, we applied the DHS 7-month gestational age cutoff (based on WHO 28-week definition) whereas a more precise gestational age reporting could strengthen future models. Importantly, the validity of the estimates relies on assumptions regarding the relationship between stillbirth and very early neonatal mortality (e.g., Stillbirth:D0-1 ratio = 1.89; threshold = 2.4), which are based on available evidence, and may not hold across settings. Accordingly, the results should be interpreted as model-based, calibrated adjustments rather than direct measures.

As highlighted by Campbell and colleagues, politics will play a pivotal role in shaping maternal and newborn health (MNH) outcomes through policies, resource allocation, and health system reform. strategies, laws and financing to improve [31]. The 77th World Health Assembly in 2024 passed a resolution, calling for urgent global action to reduce maternal, newborn, and child mortality. This marks a crucial turning point in reinvigorating global commitments to equity and survival in maternal and newborn health [32].

Our analysis contributes to the growing recognition that tailored, context-sensitive strategies are essential for reducing the overall perinatal mortality, comprehensive of stillbirth and early neonatal mortality. By leveraging detailed reproductive calendar data from over 733,000 women across 17 countries and adjusting for data quality limitations, we demonstrated how national trends can differ significantly—even within the same region—highlighting the critical importance of granular, country-specific analyses. For instance, Egypt and Malawi showed remarkable progress due to strong public health initiatives, such as Egypt’s early investments in maternal care and Malawi’s crackdown on unsafe deliveries by traditional birth attendants. Conversely, countries like Zimbabwe and Jordan, despite high levels of antenatal and institutional care coverage, saw stagnation or even reversals, likely due to systemic barriers such as economic instability and inadequate postnatal services.

The inclusion of data quality adjustments—accounting for underreporting and misclassification—ensures that mortality trends are not simply artifacts of poor survey reporting. This is particularly vital in settings where weak civil registration systems make household surveys the primary data source. Our findings demonstrate that incorporating metrics for stillbirth and early neonatal mortality, when paired with quality checks, can yield more reliable and actionable insights for health policy.

Moreover, we found that structural determinants such as HDI, wealth, place of residence, and maternal age significantly influence the pace of mortality reduction. In countries like Senegal and Bangladesh, improvements in perinatal mortality occurred without corresponding reductions in stillbirths, underscoring gaps in care continuity—from antenatal to intrapartum. These patterns stress the need to use household surveys not only for tracking outcomes but also for diagnosing where along the care continuum interventions are failing.

Ultimately, our work illustrates that maximizing the importance of household surveys requires both technical adjustments and policy contextualization. Countries can harness this dual approach to identify bottlenecks in their maternal-newborn health systems, align resources where needs are greatest, and design interventions that are locally relevant and equity-focused. Doing so will be essential for accelerating progress toward the SDG target, especially in the 64 countries currently off-track.

## Supporting information

S1 Table Number of clusters, mothers and pregnancies, by survey. (PDF)

S2 Table Excluded surveys. (PDF)

S3 Table Data quality indicators and survey round. (PDF)

S4 Table Number of pregnancies with 7+ months gestation, pregnancy outcome (unweighted). (PDF)

S5 Table Pairwise correlations between the Data Quality indicators and stillbirth and early neonatal mortality rates, by selected covariates. (PDF)

S1 Fig Sensitivity analysis: comparing model-based adjustment against alternative adjustment. (PDF)

S2 Fig Differences between model-based rate and alternative adjustment rate. (PDF)

S3 Fig Trends in Stillbirth, Early neonatal and Perinatal mortality. (PDF)

S4 Fig Rate ratios of perinatal mortality, by survey and covariates. (PDF)

S5 Fig Correlation of HDI with stillbirth, early neonatal and perinatal mortality rates. (PDF)

S1 Data Data used to generate figures. (XLSX)

S1 File Inclusivity in global research. (DOCX)

## Abbreviations

ANC: antenatal care
aRRs: adjusted rate ratios
DHS: Demographic and Health Surveys
FBH: Full Birth History
HDI: Human Development Index
LMICs: low- and middle-income countries
mGM: modified Gompertz–Makeham
MNH: maternal and newborn health
PMTCT: prevention of mother-to-child transmission
SDGs: Sustainable Development Goals
UN IGME: United Nations Inter-Agency Group for Child Mortality Estimation
