# Supplementary material for: Trends of stillbirth, early neonatal and perinatal mortality rates and sociodemographic differentials of perinatal mortality rates in 17 low- and middle-income countries: A modeling study
Source: PLoS Med. 2026 Aug 24;23(8):e1004791. doi: 10.1371/journal.pmed.1004791 (PMC13502698; doi:10.1371/journal.pmed.1004791)
Supplement: Supplementary file 1 [file pmed.1004791.s001.pdf]

S1-Table: Number of clusters, mothers and pregnancies, by survey

Region<br>Survey	Survey round	Sample	No. of Clusters	No. of households	HHs per cluster	No. of women	Women per HH	No. of pregnancie	Pregnancies per woman	Number of pregnancies per woman<br>1	2	3	4	5+
**Sub-Saharan Africa**														
Ghana	2003	AW	411	2,600	6.33	2,821	1.09	3,967	1.41	1,796	917	96	11	1
Ghana	2008	AW	408	2,059	5.05	2,188	1.06	3,074	1.40	1,401	695	85	7	0
Ghana	2014	AW	427	4,148	9.71	4,367	1.05	6,004	1.37	2,904	1,304	144	15	0
Ghana	2022/23	AW	618	6,603	10.68	7,038	1.07	9,481	1.35	4,826	1,991	211	10	0
Kenya	1998	AW	529	3,545	6.70	3,790	1.07	5,586	1.47	2,230	1,339	207	13	1
Kenya	2003	AW	400	3,813	9.53	4,020	1.05	6,020	1.50	2,323	1,417	257	23	0
Kenya	2008/9	AW	398	3,906	9.81	4,114	1.05	6,149	1.49	2,400	1,416	279	16	3
Kenya	2014	AW	1,573	6,971	4.43	7,240	1.04	10,232	1.41	4,641	2,221	364	13	1
Kenya	2022	AW	1,665	7,205	4.33	7,500	1.04	10,119	1.35	5,266	1,882	321	29	2
Malawi	2000	AW	559	7,692	13.76	8,112	1.05	12,372	1.53	4,363	3,273	445	27	4
Malawi	2004/5	AW	521	7,197	13.81	7,444	1.03	11,214	1.51	4,065	3,011	346	21	1
Malawi	2010	AW	849	13,313	15.68	13,839	1.04	20,104	1.45	8,208	5,027	575	28	1
Malawi	2015/16	AW	850	13,055	15.36	13,551	1.04	17,387	1.28	9,968	3,345	225	11	2
Malawi	2024	AW	769	9,854	12.81	10,268	1.04	11,882	1.16	8,739	1,446	81	2	0
Mali	2001	AW	402	1,929	4.80	8,278	4.29	12,755	1.54	4,301	3,506	443	27	1
Mali	2006	AW	407	7,893	19.39	9,156	1.16	14,644	1.60	4,488	3,894	734	35	5
Mali	2012/13	AW	413	5,997	14.52	6,757	1.13	10,398	1.54	3,627	2,659	434	34	3
Mali	2018	AW	345	5,677	16.46	6,442	1.13	10,150	1.58	3,292	2,623	499	25	3
Nigeria	2008	AW	886	15,978	18.03	18,294	1.14	29,416	1.61	8,866	7,858	1,454	108	8
Nigeria	2013	AW	896	17,826	19.90	20,385	1.14	31,486	1.54	10,759	8,232	1,323	63	8
Nigeria	2018	AW	1,389	19,406	13.97	22,046	1.14	34,904	1.58	11,152	9,072	1,690	124	8
Nigeria	2023/24	AW	1,373	16,647	12.12	18,528	1.11	28,059	1.51	10,364	6,886	1,201	66	11
Rwanda	2000	AW	443	4,923	11.11	5,032	1.02	8,149	1.62	2,434	2,119	440	38	1
Rwanda	2005	AW	462	5,338	11.55	5,463	1.02	8,725	1.60	2,699	2,293	447	21	3
Rwanda	2010/11	AW	492	6,233	12.67	6,392	1.03	9,147	1.43	3,980	2,092	297	23	0
Rwanda	2014/15	AW	492	5,873	11.94	6,049	1.03	8,046	1.33	4,228	1,652	162	7	0
Rwanda	2019/20	AW	500	6,069	12.14	6,211	1.02	8,180	1.32	4,420	1,624	157	9	1
Senegal	2005	AW	376	4,795	12.75	7,213	1.50	10,980	1.52	3,909	2,872	402	29	1
Senegal	2010/11	AW	391	5,316	13.60	8,210	1.54	12,414	1.51	4,537	3,174	472	22	5
Senegal	2015	AW	214	2,989	13.97	4,746	1.59	6,998	1.47	2,738	1,778	217	12	1
Senegal	2016	AW	214	2,976	13.91	4,637	1.56	6,824	1.47	2,673	1,750	205	9	0
Senegal	2018	AW	214	3,030	14.16	4,777	1.58	6,839	1.43	2,927	1,645	198	7	0
Senegal	2019	AW	214	2,901	13.56	4,428	1.53	6,271	1.42	2,759	1,504	156	9	0
Senegal	2023	AW	399	5,045	12.64	7,575	1.50	10,337	1.36	5,051	2,296	218	10	0

Tanzania	2004/5	AW	475	5,243	11.04	5,780	1.10	8,875	1.54	3,137	2,220	397	24	2
Tanzania	2009/10	AW	475	4,866	10.24	5,338	1.10	7,804	1.46	3,176	1,876	271	13	2
Tanzania	2015/16	AW	608	6,418	10.56	7,136	1.11	10,415	1.46	4,322	2,374	417	22	1
Tanzania	2022	AW	628	7,116	11.33	7,684	1.08	10,658	1.39	5,060	2,290	318	16	0
Uganda	2000/1	AW	297	4,052	13.64	4,314	1.06	7,327	1.70	1,874	1,903	502	34	1
Uganda	2006	AW	368	4,737	12.87	5,058	1.07	8,441	1.67	2,272	2,231	515	38	2
Uganda	2011	AW	404	4,667	11.55	4,980	1.07	8,117	1.63	2,372	2,101	487	18	2
Uganda	2016	AW	696	9,757	14.02	10,394	1.07	15,789	1.52	5,768	3,899	687	38	2
Zambia	2007	AW	319	3,909	12.25	4,214	1.08	6,538	1.55	2,185	1,756	253	18	2
Zambia	2013/14	AW	721	8,790	12.19	9,477	1.08	13,697	1.45	5,730	3,297	427	23	0
Zambia	2018/19	AW	545	6,850	12.57	7,457	1.09	10,108	1.36	5,050	2,175	220	12	0
Zambia	2024	AW	544	6,319	11.62	6,878	1.09	8,489	1.23	5,368	1,416	87	7	0
Zimbabwe	1994	AW	230	2,741	11.92	3,064	1.12	4,169	1.36	2,064	901	95	3	1
Zimbabwe	1999	AW	228	2,605	11.43	2,836	1.09	3,702	1.31	2,050	712	70	2	2
Zimbabwe	2005/6	AW	398	3,799	9.55	4,125	1.09	5,305	1.29	3,051	973	97	3	1
Zimbabwe	2010/11	AW	406	4,190	10.32	4,470	1.07	5,715	1.28	3,313	1,073	81	2	1
Zimbabwe	2015	AW	399	4,530	11.35	4,860	1.07	6,125	1.26	3,697	1,064	96	3	0
**North Africa Western Asia and Europe**														
Egypt	1992/93	EMW	377	5,229	13.87	5,791	1.11	8,968	1.55	3,212	2,036	491	49	3
Egypt	1995/96	EMW	927	7,333	7.91	8,135	1.11	12,420	1.53	4,596	2,853	630	52	4
Egypt	2000	EMW	500	7,157	14.31	7,919	1.11	11,182	1.41	5,081	2,447	361	27	3
Egypt	2005	EMW	682	9,087	13.32	10,025	1.10	13,788	1.38	6,655	3,000	350	18	2
Egypt	2008	EMW	1,242	7,555	6.08	8,049	1.07	10,719	1.33	5,619	2,204	214	10	2
Egypt	2014	EMW	874	11,018	12.61	11,569	1.05	15,817	1.37	7,721	3,477	343	27	1
Jordan	1990	EMW	348	4,309	12.38	4,479	1.04	8,571	1.91	1,626	1,780	919	142	12
Jordan	1997	EMW	300	3,624	12.08	3,761	1.04	6,578	1.75	1,624	1,529	542	63	3
Jordan	2002	EMW	495	3,793	7.66	3,876	1.02	6,213	1.60	1,999	1,451	394	30	2
Jordan	2007	EMW	927	6,721	7.25	6,825	1.02	10,704	1.57	3,612	2,587	588	36	2
Jordan	2012	EMW	806	6,711	8.33	6,820	1.02	10,416	1.53	3,843	2,418	505	50	4
Jordan	2017/18	EMW	964	7,216	7.49	7,355	1.02	10,731	1.46	4,475	2,421	422	37	0
**South & Southeast Asia**														
Bangladesh	1993/94	EMW	301	4,941	16.42	5,301	1.07	7,344	1.39	3,478	1,611	205	6	1
Bangladesh	1996/97	EMW	313	4,439	14.18	4,711	1.06	6,472	1.37	3,146	1,379	177	8	1
Bangladesh	1999/0	EMW	341	4,967	14.57	5,319	1.07	7,125	1.34	3,694	1,453	163	9	0
Bangladesh	2004	EMW	361	5,103	14.14	5,491	1.08	7,216	1.31	3,951	1,364	167	9	0
Bangladesh	2007	EMW	361	4,658	12.90	4,982	1.07	6,262	1.26	3,813	1,066	96	6	1
Bangladesh	2011	EMW	600	6,963	11.61	7,439	1.07	9,006	1.21	5,977	1,362	95	5	0
Bangladesh	2014	EMW	600	6,612	11.02	7,026	1.06	8,180	1.16	5,954	992	78	2	0
Bangladesh	2017/18	EMW	672	7,192	10.70	7,683	1.07	8,981	1.17	6,465	1,140	76	2	0
Bangladesh	2022	EMW	674	7,340	10.89	7,791	1.06	8,968	1.15	6,673	1,060	57	1	0
Indonesia	1991	EMW	1,068	11,332	10.61	11,770	1.04	15,982	1.36	8,076	3,208	456	28	2

Indonesia	1994	EMW	1,414	13,787	9.75	14,086	1.02	18,625	1.32	10,057	3,539	470	20	0
Indonesia	1997	EMW	1,413	13,694	9.69	13,934	1.02	17,780	1.28	10,480	3,083	352	17	2
Indonesia	2002/3	EMW	1,387	13,153	9.48	13,576	1.03	16,544	1.22	10,824	2,546	197	8	1
Indonesia	2007	EMW	1,694	15,020	8.87	15,482	1.03	18,927	1.22	12,362	2,815	286	18	1
Indonesia	2012	AW	1,826	14,777	8.09	15,342	1.04	18,180	1.18	12,718	2,422	192	8	2
Indonesia	2017	AW	1,968	15,076	7.66	15,600	1.03	18,235	1.17	13,151	2,269	174	6	0
**Latin America & Caribbean**														
Colombia	1995	AW	936	3,637	3.89	3,825	1.05	5,138	1.34	2,706	937	172	8	2
Colombia	2000	AW	923	3,420	3.71	3,599	1.05	4,658	1.29	2,665	819	105	10	0
Colombia	2004/5	AW	3,423	11,131	3.25	11,696	1.05	14,650	1.25	9,088	2,279	312	17	0
Colombia	2009/10	AW	3,770	13,416	3.56	14,435	1.08	17,606	1.22	11,567	2,577	280	10	1
Colombia	2015/16	AW	3,371	9,302	2.76	9,736	1.05	11,410	1.17	8,192	1,419	120	5	0
Peru	1991/92	AW	892	6,069	6.80	6,396	1.05	9,472	1.48	3,842	2,073	440	41	0
Peru	1996	AW	1,482	11,860	8.00	12,520	1.06	17,789	1.42	8,003	3,803	676	38	0
Peru	2000	AW	1,403	10,095	7.20	10,556	1.05	13,812	1.31	7,663	2,544	335	14	0
Peru	2004/6	AW	840	6,008	7.15	6,286	1.05	7,830	1.25	4,855	1,319	111	1	0
Peru	2007/8	AW	989	7,204	7.28	7,485	1.04	9,226	1.23	5,887	1,465	123	10	0
Peru	2009	AW	1,127	8,072	7.16	8,415	1.04	10,249	1.22	6,722	1,554	137	2	0
Peru	2010	AW	1,124	7,316	6.51	7,590	1.04	9,180	1.21	6,088	1,415	86	1	0
Peru	2011	AW	1,121	7,257	6.47	7,535	1.04	9,102	1.21	6,085	1,337	109	4	0
Peru	2012	AW	1,400	7,638	5.46	7,994	1.05	9,610	1.20	6,497	1,382	111	4	0
**All countries**			74,476	670,623	10.67	733,181	1.13	1,019,253	1.40	483,565	215,509	31,922	2,039	146

AW=All woman, EMW=Ever-married woman, HH=Household
