# Supplementary material for: Trends of stillbirth, early neonatal and perinatal mortality rates and sociodemographic differentials of perinatal mortality rates in 17 low- and middle-income countries: A modeling study
Source: PLoS Med. 2026 Aug 24;23(8):e1004791. doi: 10.1371/journal.pmed.1004791 (PMC13502698; doi:10.1371/journal.pmed.1004791)
Supplement: Supplementary file 2 [file pmed.1004791.s002.pdf]

S2 Table: Excluded survey

**Country**	**Survey round**	Stillbirth	Death D0-1
Armenia	2000	28	17
Armenia	2005	12	4
Armenia	2010	10	5
Armenia	2015/16	10	3
Colombia	1990	47	22
Jordan	2023	34	15
