# Supplementary material for: Trends of stillbirth, early neonatal and perinatal mortality rates and sociodemographic differentials of perinatal mortality rates in 17 low- and middle-income countries: A modeling study
Source: PLoS Med. 2026 Aug 24;23(8):e1004791. doi: 10.1371/journal.pmed.1004791 (PMC13502698; doi:10.1371/journal.pmed.1004791)
Supplement: Supplementary file 3 [file pmed.1004791.s003.pdf]

S3-Table: Data quality indicators, and survey round

Region				
Survey	Survey round	Stillbirth to D0-1 ratio	D0-1 to D2-6 ratio	Heaping Index at day 7
**Sub-Saharan Africa**				
Ghana	2003	0.50	1.76	1.95
Ghana	2008	0.82	2.21	2.55
Ghana	2014	0.73	4.28	2.78
Ghana	2022/23	1.65	2.31	2.62
Kenya	1998	0.66	2.38	3.08
Kenya	2003	0.81	2.01	2.11
Kenya	2008/9	0.74	2.16	3.82
Kenya	2014	1.32	1.89	3.66
Kenya	2022	1.51	2.05	2.21
Malawi	2000	0.79	1.52	3.01
Malawi	2004/5	1.20	2.14	2.74
Malawi	2010	0.95	2.86	3.64
Malawi	2015/16	0.97	2.80	3.72
Malawi	2024	1.30	2.60	2.99
Mali	2001	0.53	1.92	2.13
Mali	2006	0.65	1.60	1.85
Mali	2012/13	0.29	3.67	1.69
Mali	2018	0.78	1.50	1.60
Nigeria	2008	0.41	1.97	1.67
Nigeria	2013	0.76	1.59	1.07
Nigeria	2018	0.77	2.58	1.72
Nigeria	2023/24	0.80	2.67	1.91
Rwanda	2000	0.90	2.16	2.87
Rwanda	2005	1.08	2.39	2.87
Rwanda	2010/11	1.41	2.10	4.01
Rwanda	2014/15	1.87	1.47	3.35
Rwanda	2019/20	1.36	2.34	2.72
Senegal	2005	1.60	1.28	1.35
Senegal	2010/11	1.15	2.34	1.64
Senegal	2015	2.99	0.79	1.89
Senegal	2016	3.61	0.60	0.74
Senegal	2018	1.72	1.23	0.49
Senegal	2019	4.72	0.43	0.95
Senegal	2023	2.23	0.74	1.32
Tanzania	2004/5	1.15	2.56	2.89
Tanzania	2009/10	1.45	2.06	3.35
Tanzania	2015/16	1.35	1.77	1.80
Tanzania	2022	1.32	2.62	3.51
Uganda	2000/1	0.94	2.93	3.39
Uganda	2006	1.29	2.14	3.19

Uganda	2011	1.42	2.51	3.75
Uganda	2016	1.09	2.69	3.30
Zambia	2007	0.98	1.73	3.20
Zambia	2013/14	1.08	1.94	2.66
Zambia	2018/19	0.76	3.01	4.22
Zambia	2024	1.75	2.36	1.64
Zimbabwe	1994	1.59	1.38	2.55
Zimbabwe	1999	0.91	3.90	3.48
Zimbabwe	2005/6	0.62	2.88	3.32
Zimbabwe	2010/11	0.98	2.85	2.65
Zimbabwe	2015	0.99	1.94	3.59
**North Africa Western Asia and Europe**				
Egypt	1992/93	1.51	1.17	3.05
Egypt	1995/96	1.53	1.06	3.02
Egypt	2000	1.78	1.24	4.18
Egypt	2005	1.35	1.23	2.74
Egypt	2008	1.53	1.16	2.20
Egypt	2014	1.58	1.09	2.65
Jordan	1990	1.34	1.51	2.10
Jordan	1997	0.94	1.93	2.47
Jordan	2002	2.07	0.63	2.23
Jordan	2007	1.28	1.19	0.72
Jordan	2012	0.90	1.08	1.19
Jordan	2017/18	1.23	0.88	2.04
**South & Southeast Asia**				
Bangladesh	1993/94	1.50	1.46	1.75
Bangladesh	1996/97	2.02	1.37	1.90
Bangladesh	1999/0	1.56	1.68	1.15
Bangladesh	2004	1.92	2.00	1.59
Bangladesh	2007	1.60	2.30	1.22
Bangladesh	2011	1.73	1.62	1.36
Bangladesh	2014	1.64	1.44	0.35
Bangladesh	2017/18	1.85	1.58	1.35
Bangladesh	2022	1.96	1.75	2.11
Indonesia	1991	0.69	2.10	2.95
Indonesia	1994	0.82	2.00	1.66
Indonesia	1997	0.90	2.81	2.90
Indonesia	2002/3	0.99	2.12	1.43
Indonesia	2007	1.01	2.75	3.04
Indonesia	2012	1.06	2.28	2.85
Indonesia	2017	1.06	2.06	2.13
**Latin America & Caribbean**				
Colombia	1995	1.20	1.45	0.33
Colombia	2000	1.37	3.38	1.61
Colombia	2004/5	1.49	1.54	0.50
Colombia	2009/10	1.91	1.66	1.97
Colombia	2015/16	2.40	2.91	0.55

Peru	1991/92	0.90	1.80	1.59
Peru	1996	0.81	2.75	1.84
Peru	2000	1.31	2.02	2.36
Peru	2004/6	1.17	5.12	2.38
Peru	2007/8	3.35	1.98	0.77
Peru	2009	2.52	1.25	1.61
Peru	2010	2.51	0.79	0.72
Peru	2011	3.28	1.49	2.92
Peru	2012	2.06	1.31	1.82
