# Supplementary material for: Trends of stillbirth, early neonatal and perinatal mortality rates and sociodemographic differentials of perinatal mortality rates in 17 low- and middle-income countries: A modeling study
Source: PLoS Med. 2026 Aug 24;23(8):e1004791. doi: 10.1371/journal.pmed.1004791 (PMC13502698; doi:10.1371/journal.pmed.1004791)
Supplement: Supplementary file 4 [file pmed.1004791.s004.pdf]

S4 Table: Number of pregnancies with 7+ months gestation, pregnancy outcome (unweighted), by country and survey round

Region<br>Country name	Survey round	Calendar mid-year	Births and pregnancies of 7+ months gestation	Stillbirth	Currently pregnant	Live births	% Singleton
**Sub-Saharan Africa**							
Ghana	2003	2001	3,967	39	164	3,764	95.9
Ghana	2008	2006	3,074	41	112	2,921	95.8
Ghana	2014	2012	6,004	73	220	5,711	95.1
Ghana	2022/23	2020	9,481	122	383	8,976	95.2
Kenya	1998	1996	5,586	53	194	5,339	97.2
Kenya	2003	2001	6,020	86	241	5,693	96.7
Kenya	2008/9	2006	6,149	57	203	5,889	97.0
Kenya	2014	2012	10,232	131	330	9,771	97.3
Kenya	2022	2020	10,119	172	382	9,565	96.7
Malawi	2000	1998	12,372	156	555	11,661	95.9
Malawi	2004/5	2002	11,214	169	489	10,556	96.6
Malawi	2010	2008	20,104	302	703	19,099	95.8
Malawi	2015/16	2013	17,387	203	658	16,526	96.2
Malawi	2024	2022	11,882	187	434	11,261	96.3
Mali	2001	1999	12,755	173	594	11,988	96.7
Mali	2006	2004	14,644	169	615	13,860	96.7
Mali	2012/13	2010	10,398	67	337	9,994	96.6
Mali	2018	2016	10,150	130	360	9,660	96.5
Nigeria	2008	2006	29,416	233	1,228	27,955	96.6
Nigeria	2013	2011	31,486	401	1,597	29,488	96.6
Nigeria	2018	2016	34,904	571	1,446	32,887	96.3
Nigeria	2023/24	2021	28,059	484	1,087	26,488	96.3
Rwanda	2000	1998	8,149	138	330	7,681	97.4
Rwanda	2005	2003	8,725	153	341	8,231	97.4
Rwanda	2010/11	2008	9,147	147	292	8,708	97.1
Rwanda	2014/15	2012	8,046	122	293	7,631	97.1
Rwanda	2019/20	2017	8,180	111	278	7,791	97.2
Senegal	2005	2003	10,980	220	408	10,352	96.4
Senegal	2010/11	2008	12,414	212	468	11,734	96.1
Senegal	2015	2013	6,998	163	256	6,579	96.2
Senegal	2016	2014	6,824	129	252	6,443	96.0
Senegal	2018	2016	6,839	143	296	6,400	96.5
Senegal	2019	2017	6,271	126	273	5,872	96.3
Senegal	2023	2021	10,337	151	365	9,821	95.8
Tanzania	2004/5	2002	8,875	185	383	8,307	95.9
Tanzania	2009/10	2008	7,804	137	334	7,333	96.9
Tanzania	2015/16	2013	10,415	213	354	9,848	96.5
Tanzania	2022	2020	10,658	184	398	10,076	97.0
Uganda	2000/1	1998	7,327	128	290	6,909	97.3
Uganda	2006	2004	8,441	123	348	7,970	97.1

Uganda	2011	2009	8,117	144	333	7,640	96.9
Uganda	2016	2014	15,789	247	602	14,940	96.7
Zambia	2007	2005	6,538	86	267	6,185	95.9
Zambia	2013/14	2011	13,697	179	480	13,038	96.8
Zambia	2018/19	2016	10,108	133	392	9,583	96.9
Zambia	2024	2022	8,489	136	333	8,020	97.0
Zimbabwe	1994	1992	4,169	57	166	3,946	97.3
Zimbabwe	1999	1997	3702	50	143	3509	96.8
Zimbabwe	2005/6	2003	5,305	42	197	5,066	96.8
Zimbabwe	2010/11	2009	5,715	72	265	5,378	97.6
Zimbabwe	2015	2013	6,125	78	203	5,844	96.8
**North Africa Western Asia and Europe**							
Egypt	1992/93	1990	8,968	138	394	8,436	97.4
Egypt	1995/96	1993	12,420	201	514	11,705	96.9
Egypt	2000	1998	11,182	164	359	10,659	96.5
Egypt	2005	2003	13,788	134	469	13,185	96.0
Egypt	2008	2006	10,719	89	398	10,232	96.0
Egypt	2014	2012	15,817	95	585	15,137	96.1
Jordan	1990	1988	8,571	98	388	8,085	97.5
Jordan	1997	1995	6,578	57	252	6,269	97.2
Jordan	2002	2000	6213	59	270	5884	97.0
Jordan	2007	2005	10,704	64	506	10,134	96.9
Jordan	2012	2010	10,416	70	363	9,983	96.4
Jordan	2017/18	2015	10,731	52	548	10,131	96.6
**South & Southeast Asia**							
Bangladesh	1993/94	1991	7,344	194	369	6,781	98.5
Bangladesh	1996/97	1994	6,472	196	303	5,973	98.2
Bangladesh	1999/0	1997	7,125	197	289	6,639	98.4
Bangladesh	2004	2001	7,216	258	232	6,726	98.1
Bangladesh	2007	2005	6,262	166	224	5,872	98.4
Bangladesh	2011	2009	9,006	225	373	8,408	98.2
Bangladesh	2014	2012	8,180	178	371	7,631	98.5
Bangladesh	2017/18	2015	8,981	215	363	8,403	98.1
Bangladesh	2022	2020	8,968	186	412	8,370	98.1
Indonesia	1991	1989	15,982	119	651	15,212	98.6
Indonesia	1994	1992	18,625	170	787	17,668	98.5
Indonesia	1997	1995	17,780	154	688	16,938	98.4
Indonesia	2002/3	2000	16544	157	637	15750	98.5
Indonesia	2007	2005	18,927	176	694	18,057	98.5
Indonesia	2012	2010	18,180	204	744	17,232	98.4
Indonesia	2017	2015	18,235	176	770	17,289	98.6
**Latin America & Caribbean**							
Colombia	1995	1993	5,138	47	191	4,900	98.7
Colombia	2000	1998	4,658	54	195	4,409	98.1
Colombia	2004/5	2002	14,650	103	501	14,046	98.4
Colombia	2009/10	2008	17,606	109	634	16,863	98.6
Colombia	2015/16	2013	11,410	83	407	10,920	98.6
Peru	1991/92	1989	9,472	93	334	9,045	98.4

Peru	1996	1994	17,789	186	585	17,018	98.4
Peru	2000	1998	13,812	129	490	13,193	98.6
Peru	2004/6	2003	7,830	69	328	7,433	98.5
Peru	2007/8	2005	9,226	109	397	8,720	98.4
Peru	2009	2007	10,249	89	374	9,786	98.5
Peru	2010	2008	9,180	70	335	8,775	98.7
Peru	2011	2009	9,102	92	323	8,687	98.6
Peru	2012	2010	9,610	73	361	9,176	98.3
