# Supplementary material for: Trends of stillbirth, early neonatal and perinatal mortality rates and sociodemographic differentials of perinatal mortality rates in 17 low- and middle-income countries: A modeling study
Source: PLoS Med. 2026 Aug 24;23(8):e1004791. doi: 10.1371/journal.pmed.1004791 (PMC13502698; doi:10.1371/journal.pmed.1004791)
Supplement: Supplementary file 5 [file pmed.1004791.s005.pdf]

S5 Table: Pair-wise correlations between the Data Quality indicators and stillbirth and early neonatal mortality rates, by selected covariates

	Heaping index at day 7	Stillbirths to deaths on D0-1	Deaths on D0-1 to deaths on D2-6
**All**			
Stillbirth rate	-0.0593	0.2613	-0.0684
Early neonatal rate	0.0898	-0.4713*	0.1544
**Place of residence**			
Stillbirth rate	-0.0937	0.2657*	-0.0574
Early neonatal rate	-0.0515	-0.4121*	0.1057
**Level of education**			
Stillbirth rate	-0.0284	0.3550*	-0.0481
Early neonatal rate	-0.0204	-0.4652*	0.1176
**Wealth index**			
Stillbirth rate	-0.0567	0.0344	-0.0691
Early neonatal rate	0.0405	-0.2112*	-0.0754
**Maternal age**			
Stillbirth rate	-0.0695	0.4556*	-0.1214
Early neonatal rate	0.0392	-0.3988*	0.0357
**Number of livebirth**			
Stillbirth rate	-0.0225	0.1453	-0.0809
Early neonatal rate	0.0903	-0.4294*	0.0502

*=p<0.01
