# Supplementary material for: Trends of stillbirth, early neonatal and perinatal mortality rates and sociodemographic differentials of perinatal mortality rates in 17 low- and middle-income countries: A modeling study
Source: PLoS Med. 2026 Aug 24;23(8):e1004791. doi: 10.1371/journal.pmed.1004791 (PMC13502698; doi:10.1371/journal.pmed.1004791)

S1.1 Fig: Sensitivity analysis: comapring model-based adjusted against alternative adjustment -- Ghana

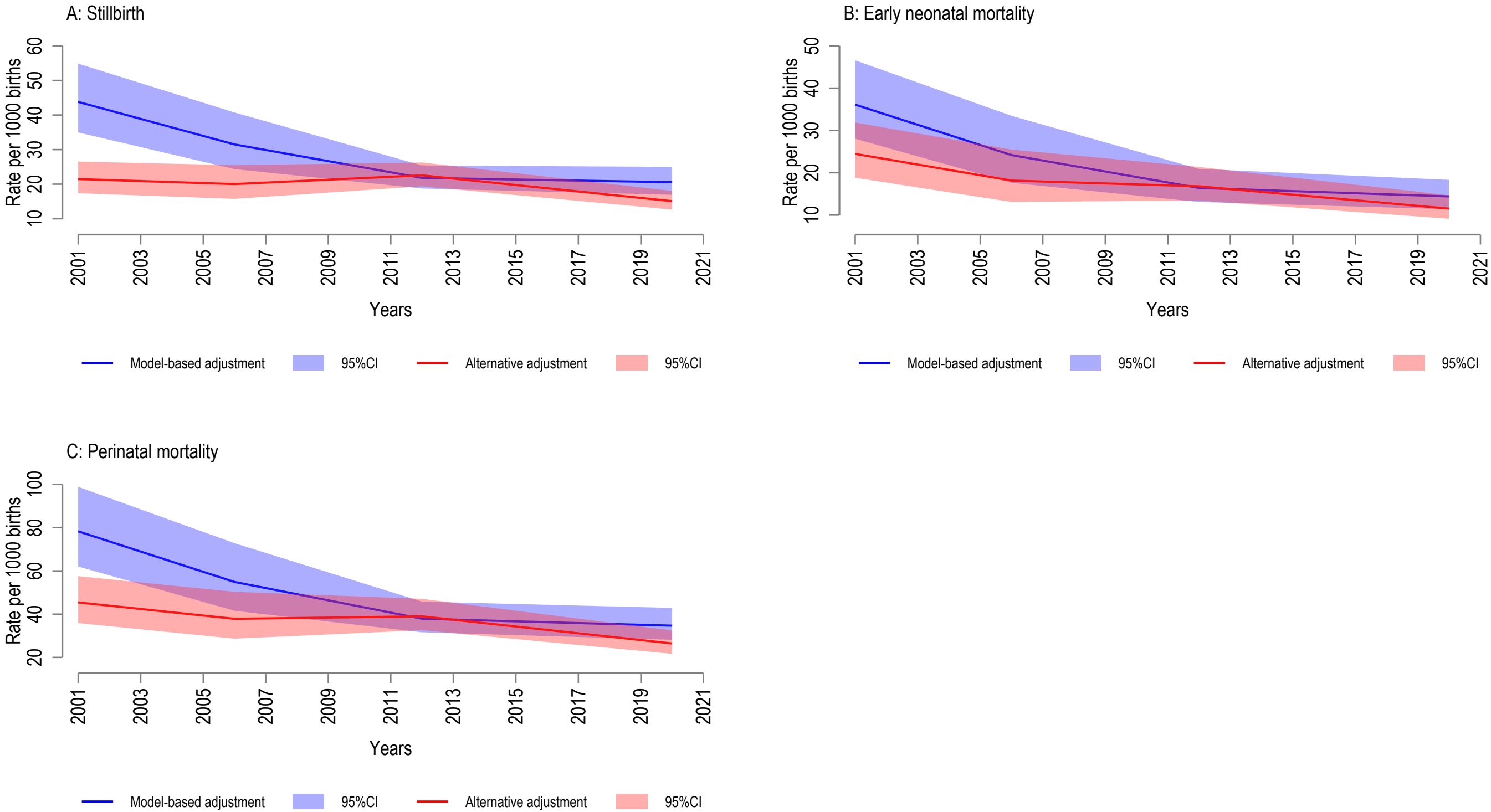

S1.2 Fig: Sensitivity analysis: comapring model-based adjusted against alternative adjustment -- Kenya

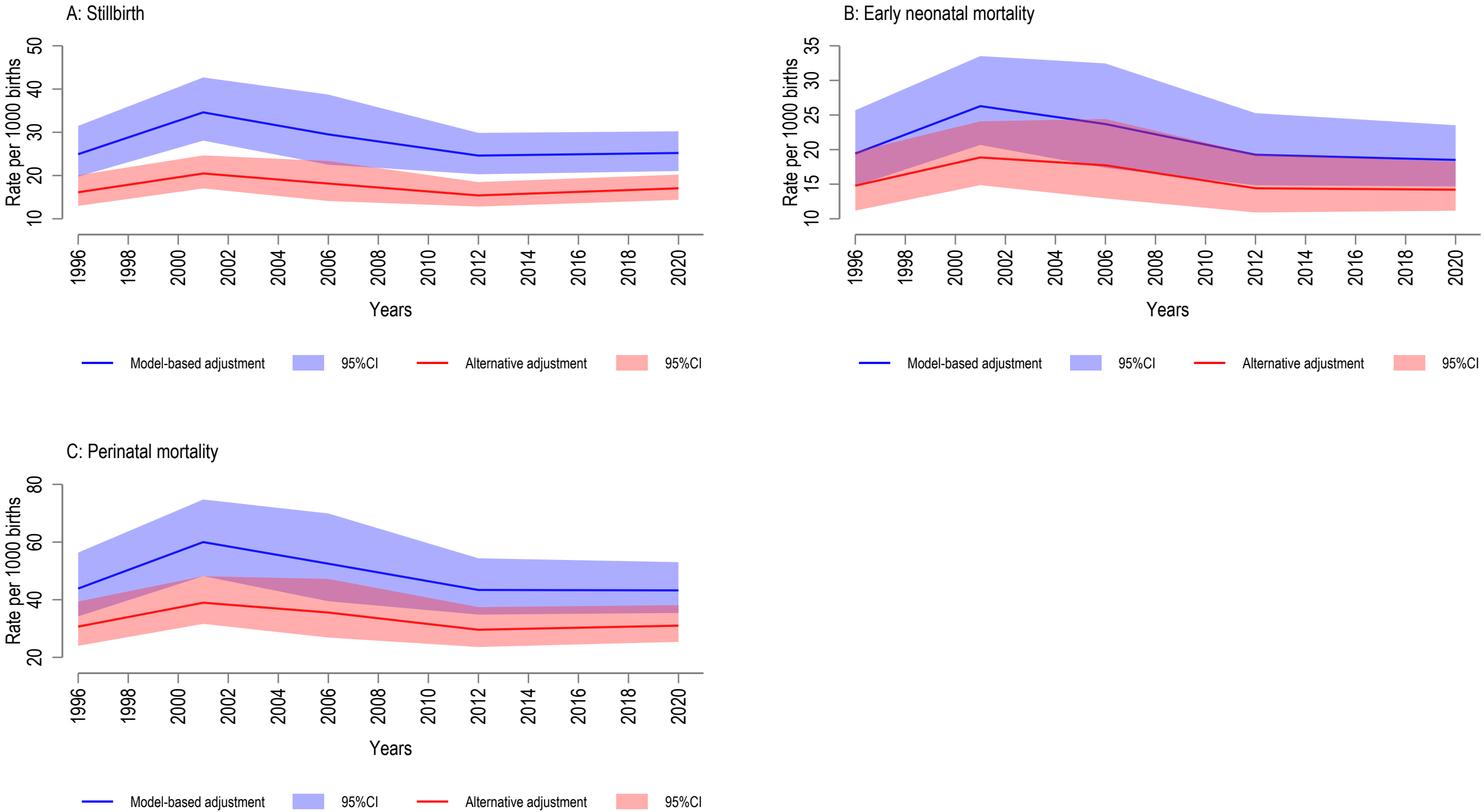

S1.3 Fig: Sensitivity analysis: comapring model-based adjusted against alternative adjustment -- Malawi

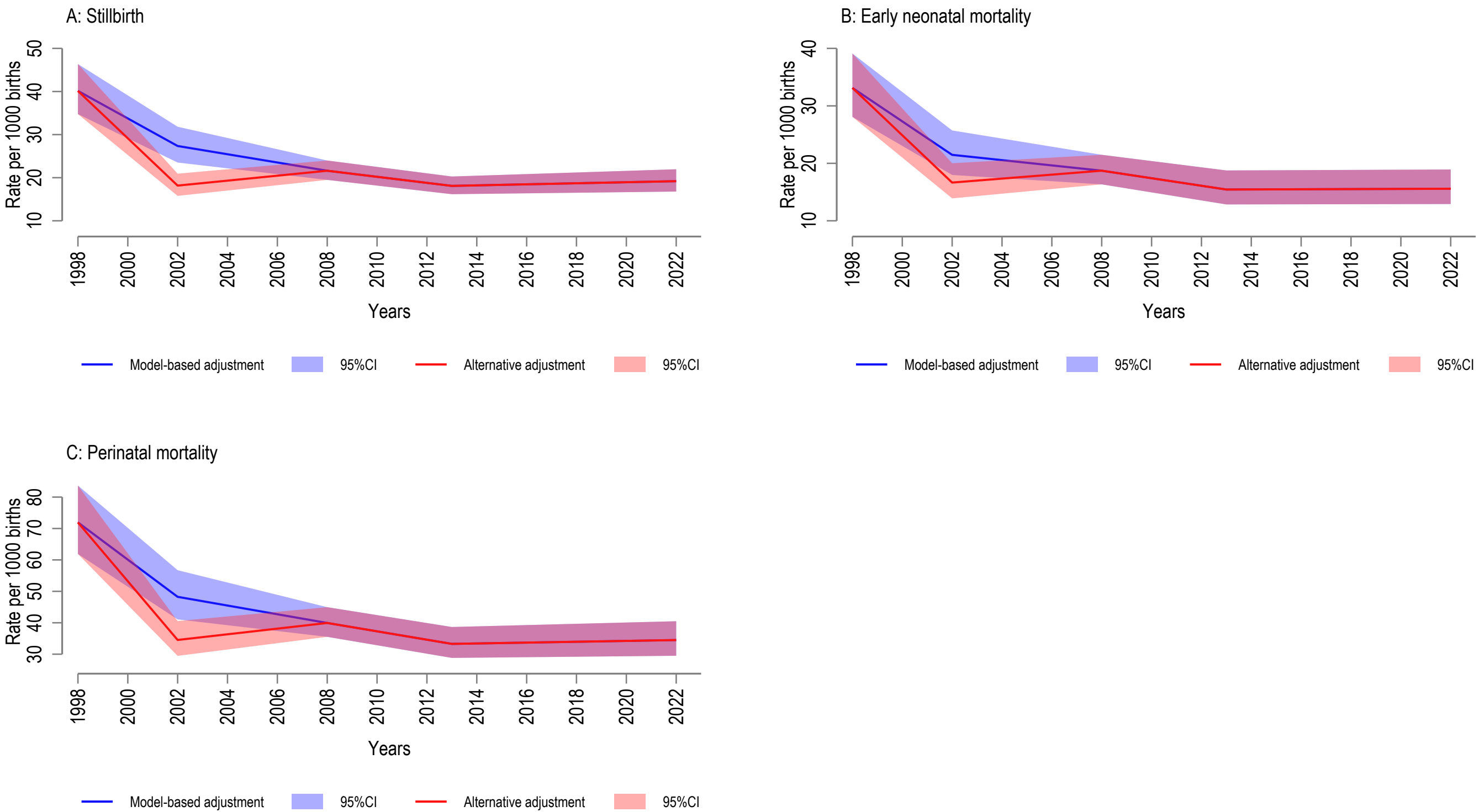

S1.4 Fig: Sensitivity analysis: comapring model-based adjusted against alternative adjustment -- Mali

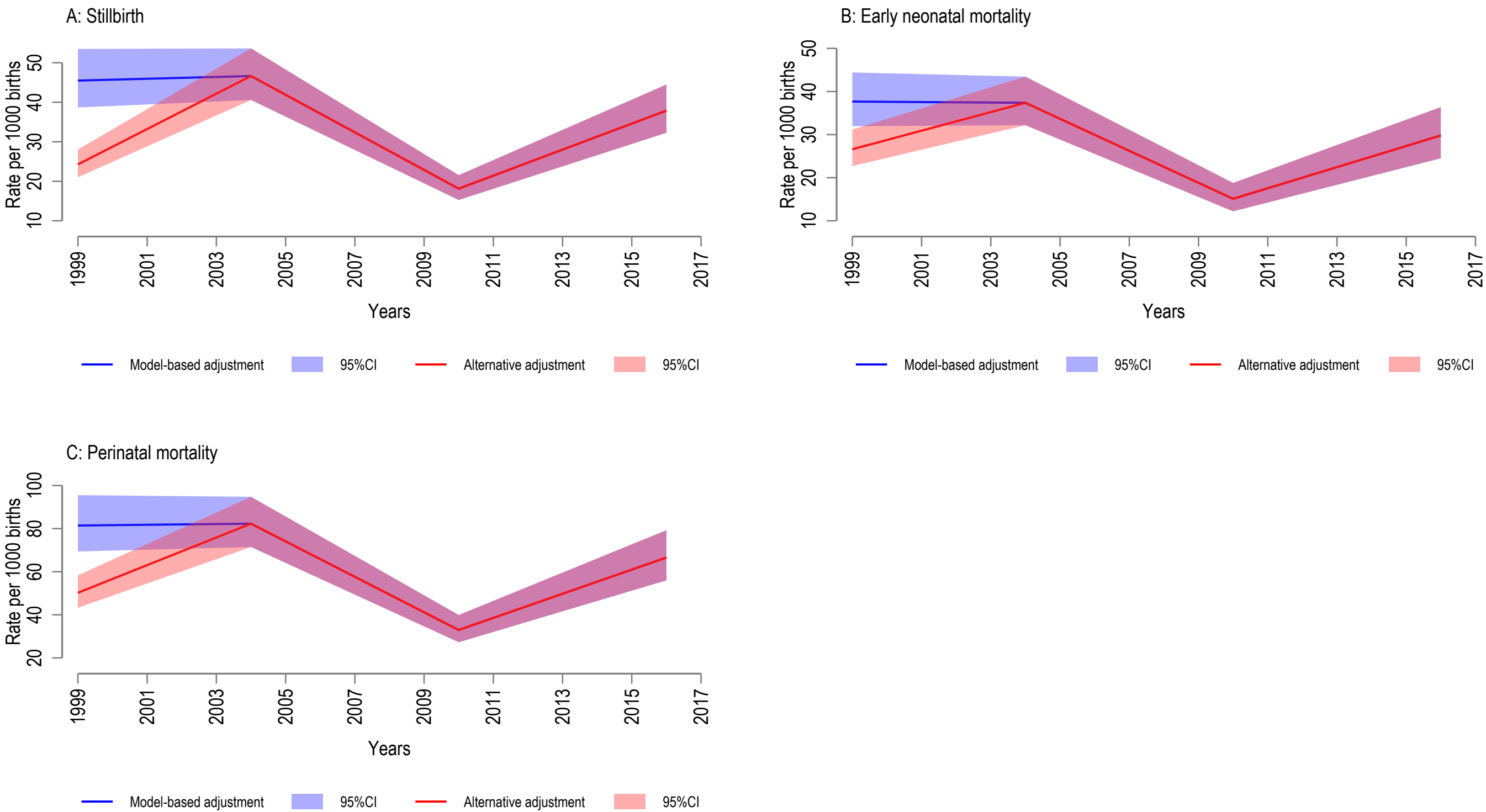

S1.5 Fig: Sensitivity analysis: comapring model-based adjusted against alternative adjustment -- Nigeria

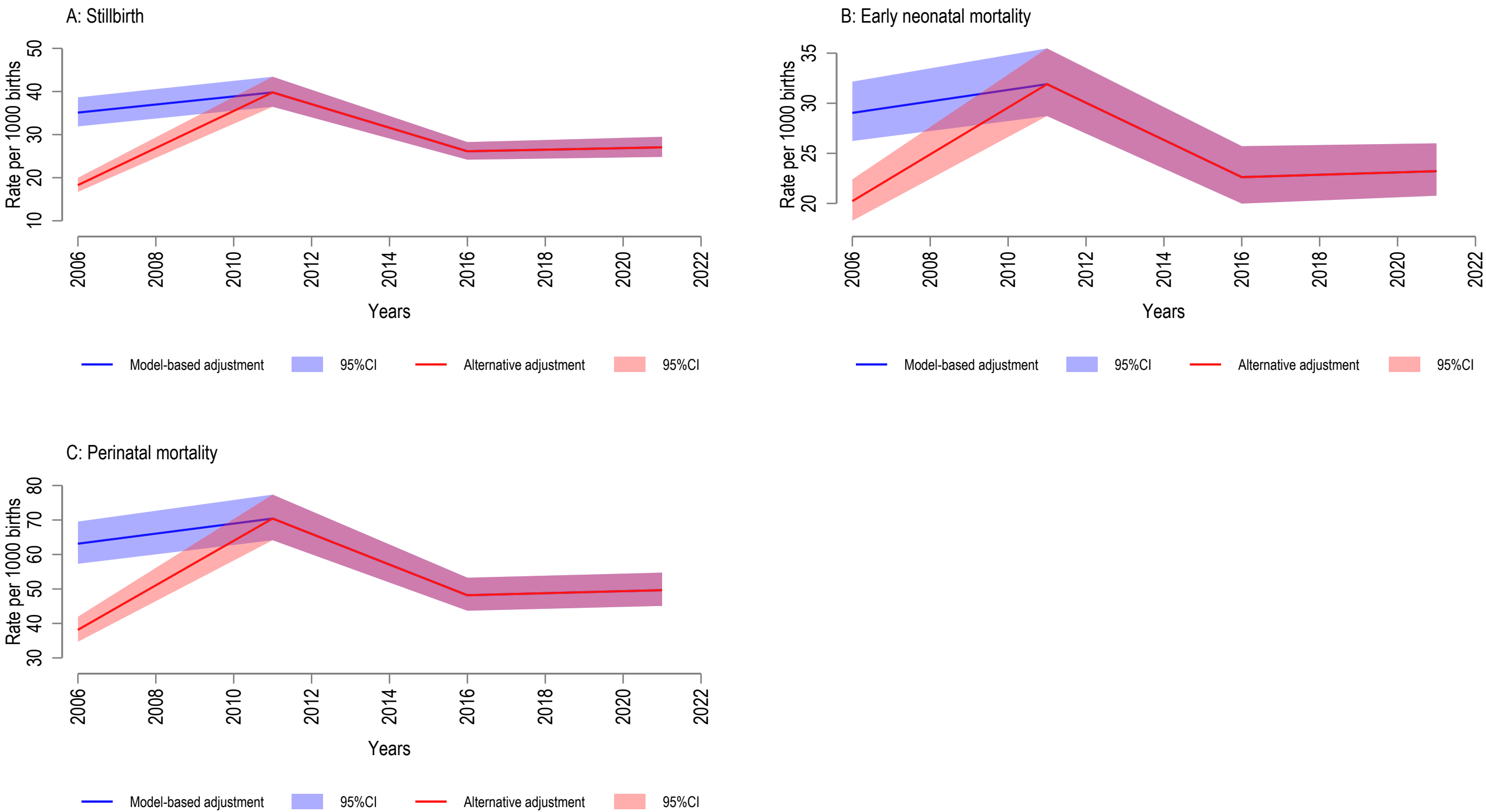

S1.6 Fig: Sensitivity analysis: comapring model-based adjusted against alternative adjustment -- Rwanda

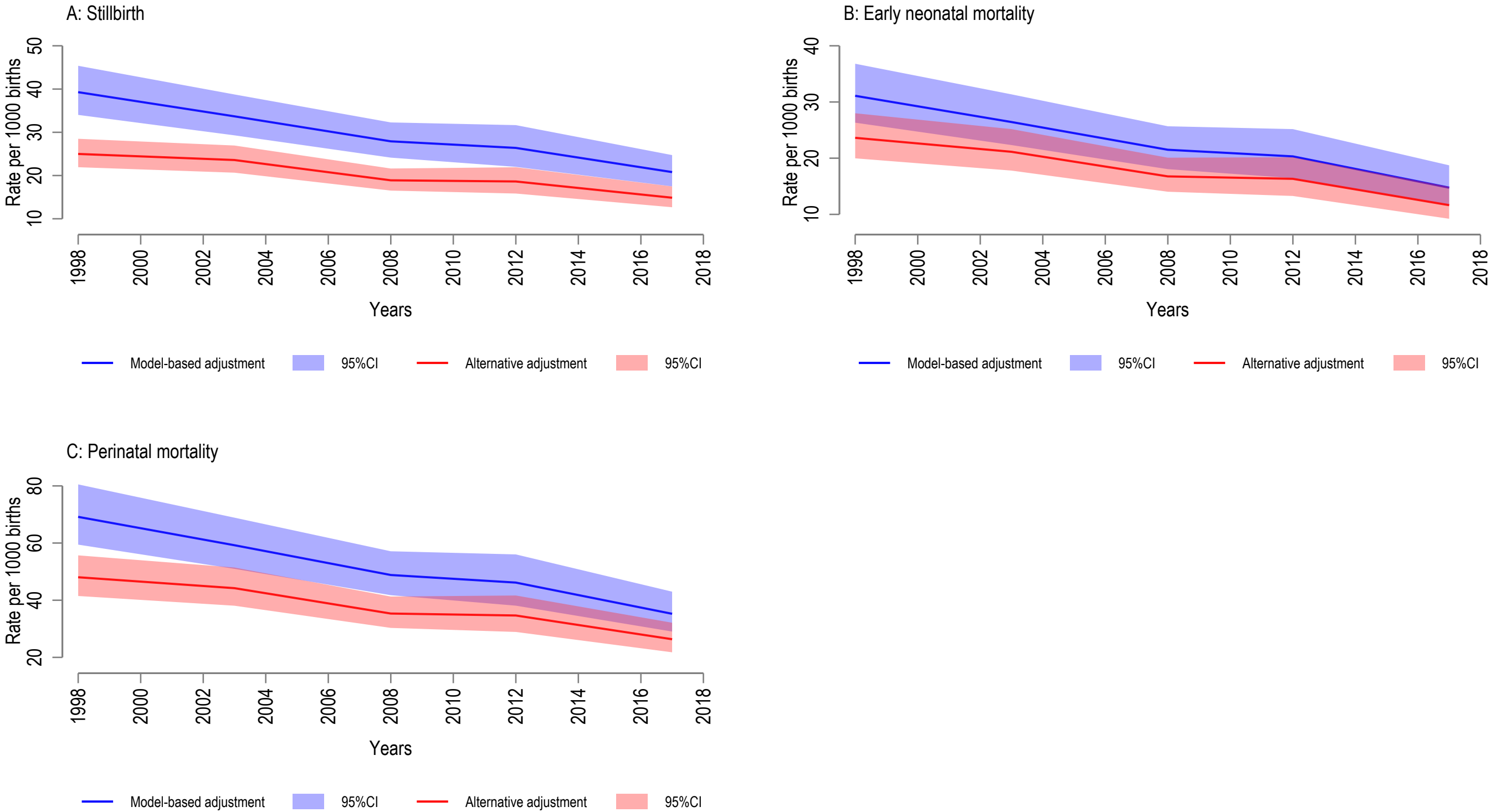

S1.7 Fig: Sensitivity analysis: comapring model-based adjusted against alternative adjustment -- Senegal

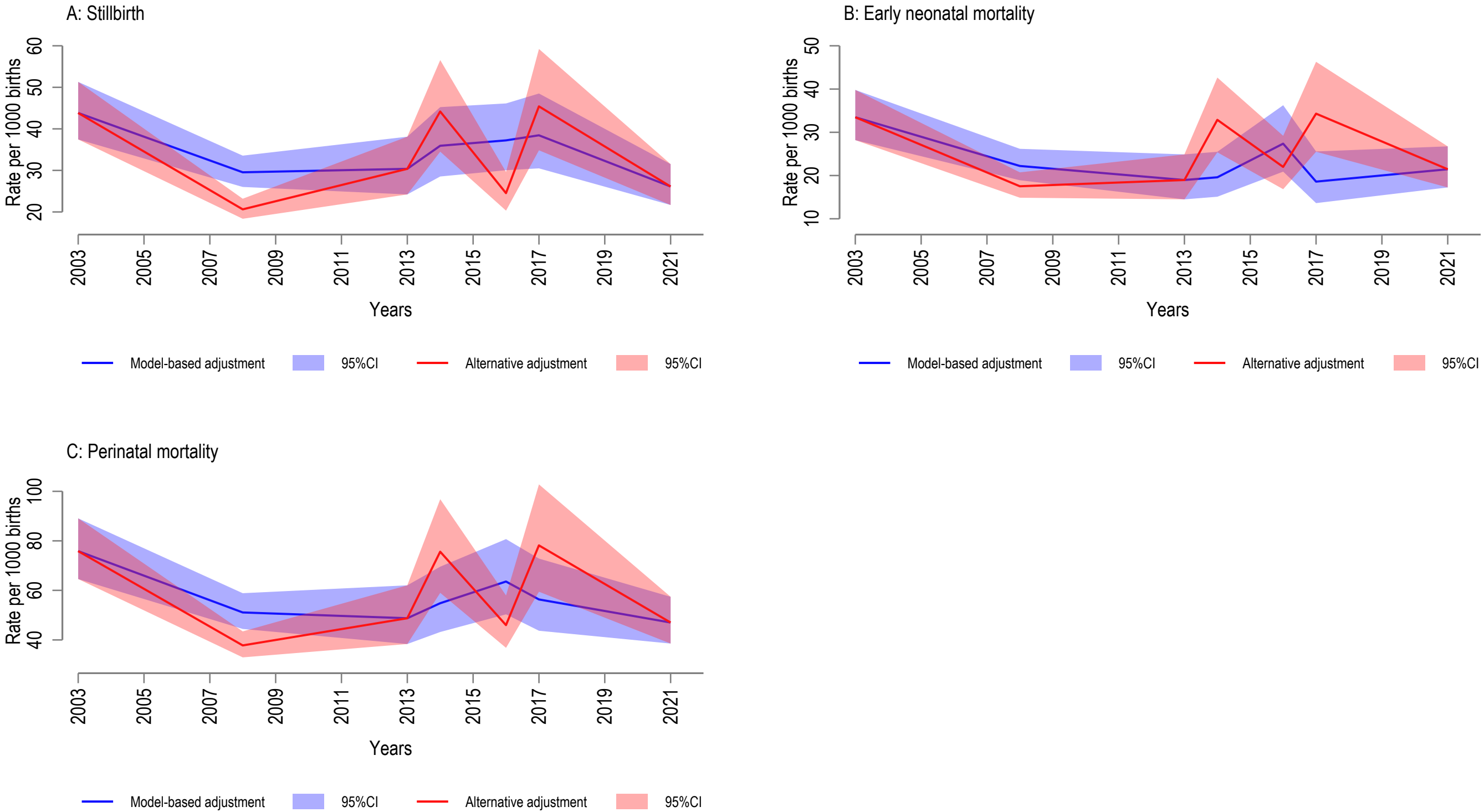

S1.8 Fig: Sensitivity analysis: comapring model-based adjusted against alternative adjustment -- Tanzania

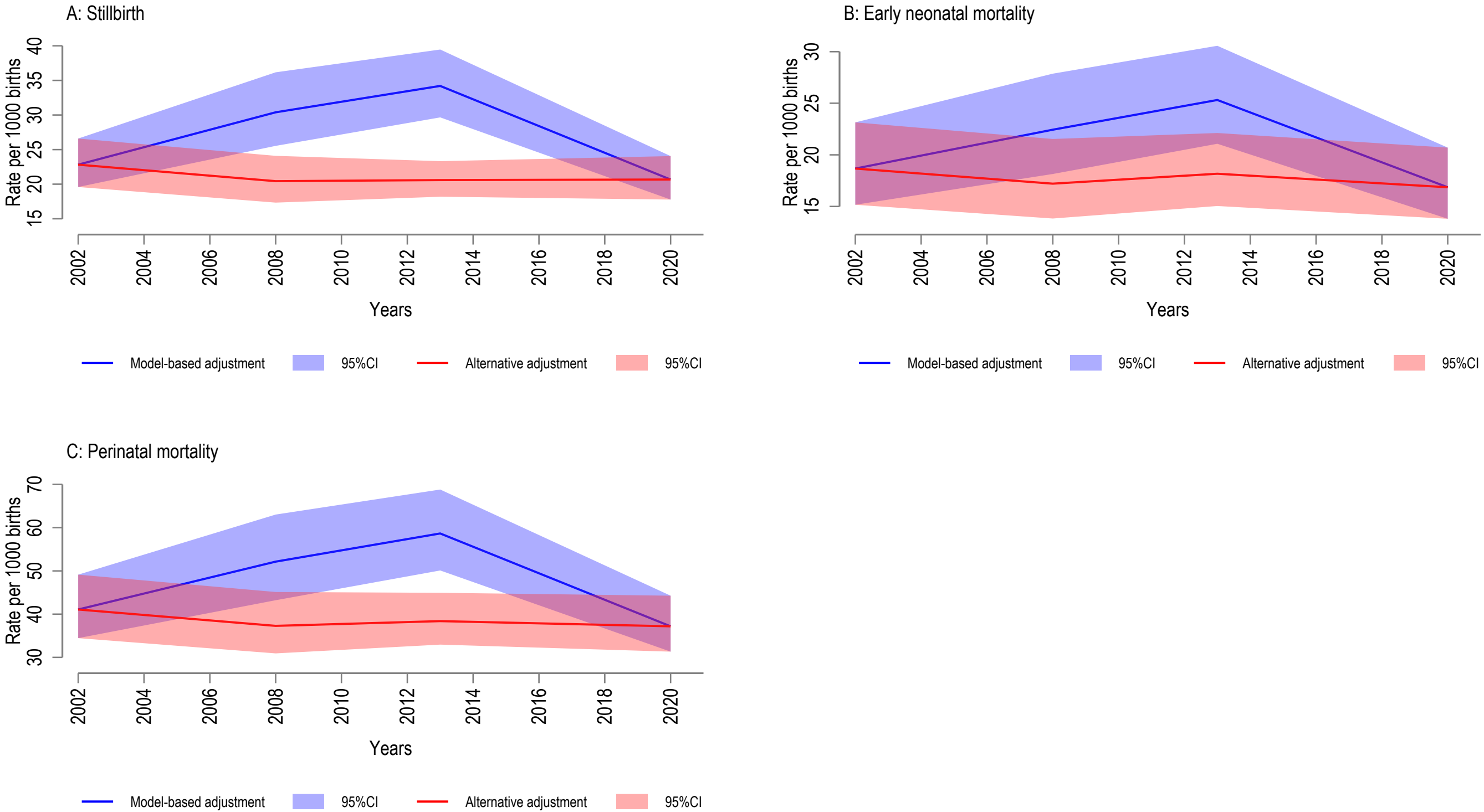

S1.9 Fig: Sensitivity analysis: comapring model-based adjusted against alternative adjustment -- Uganda

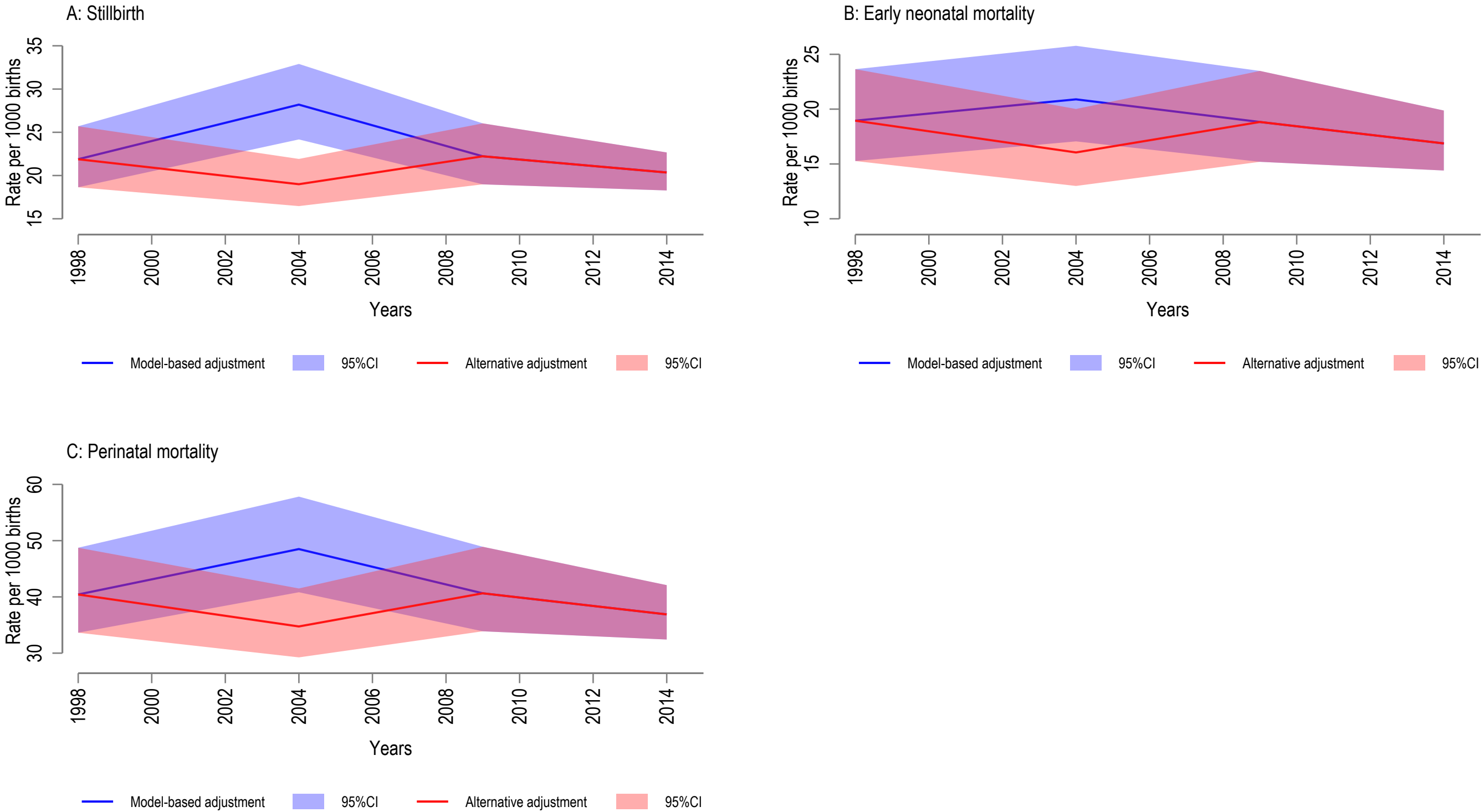

S1.10 Fig: Sensitivity analysis: comapring model-based adjusted against alternative adjustment -- Zambia

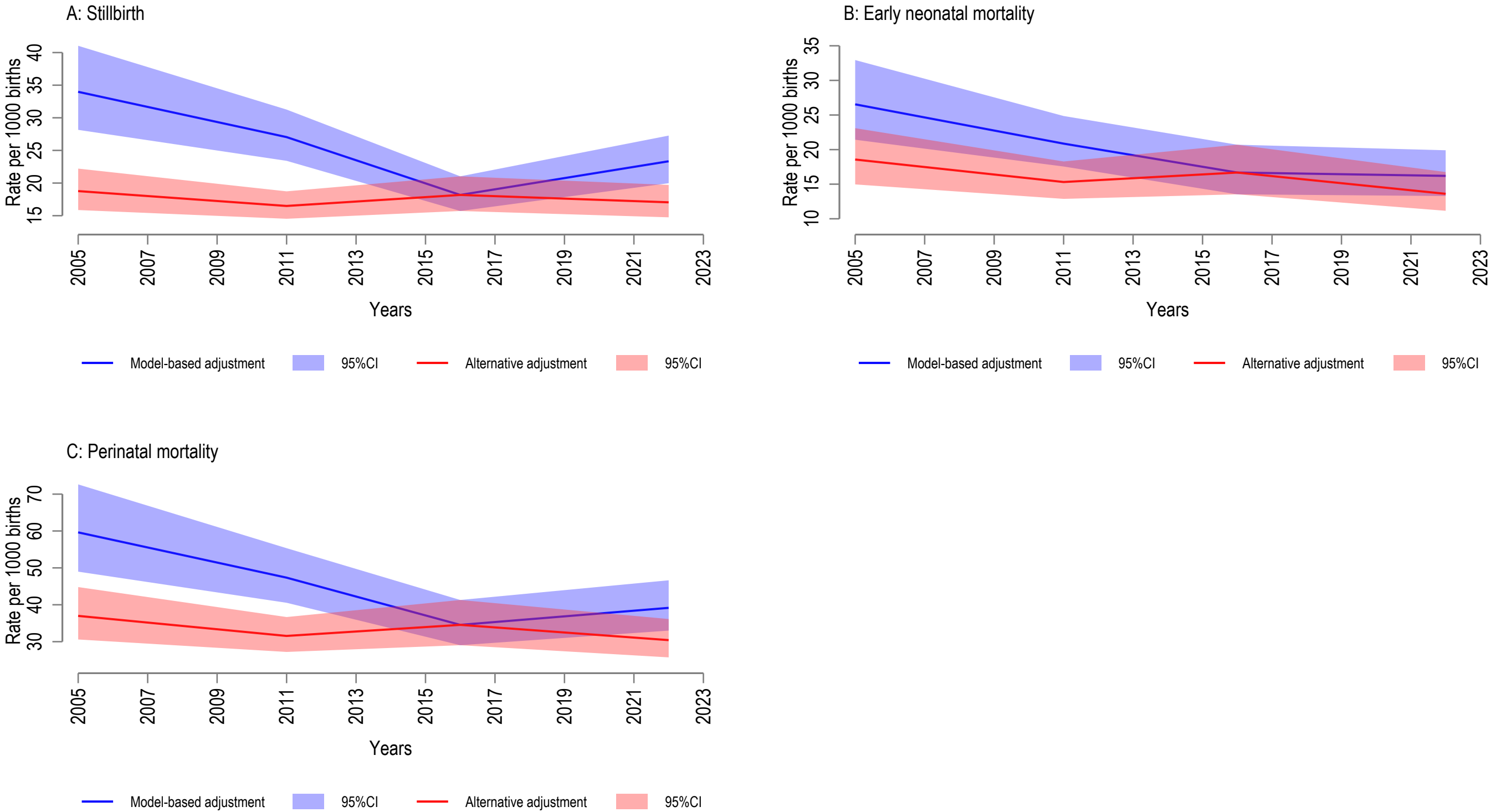

S1.11 Fig: Sensitivity analysis: comapring model-based adjusted against alternative adjustment -- Zimbabwe

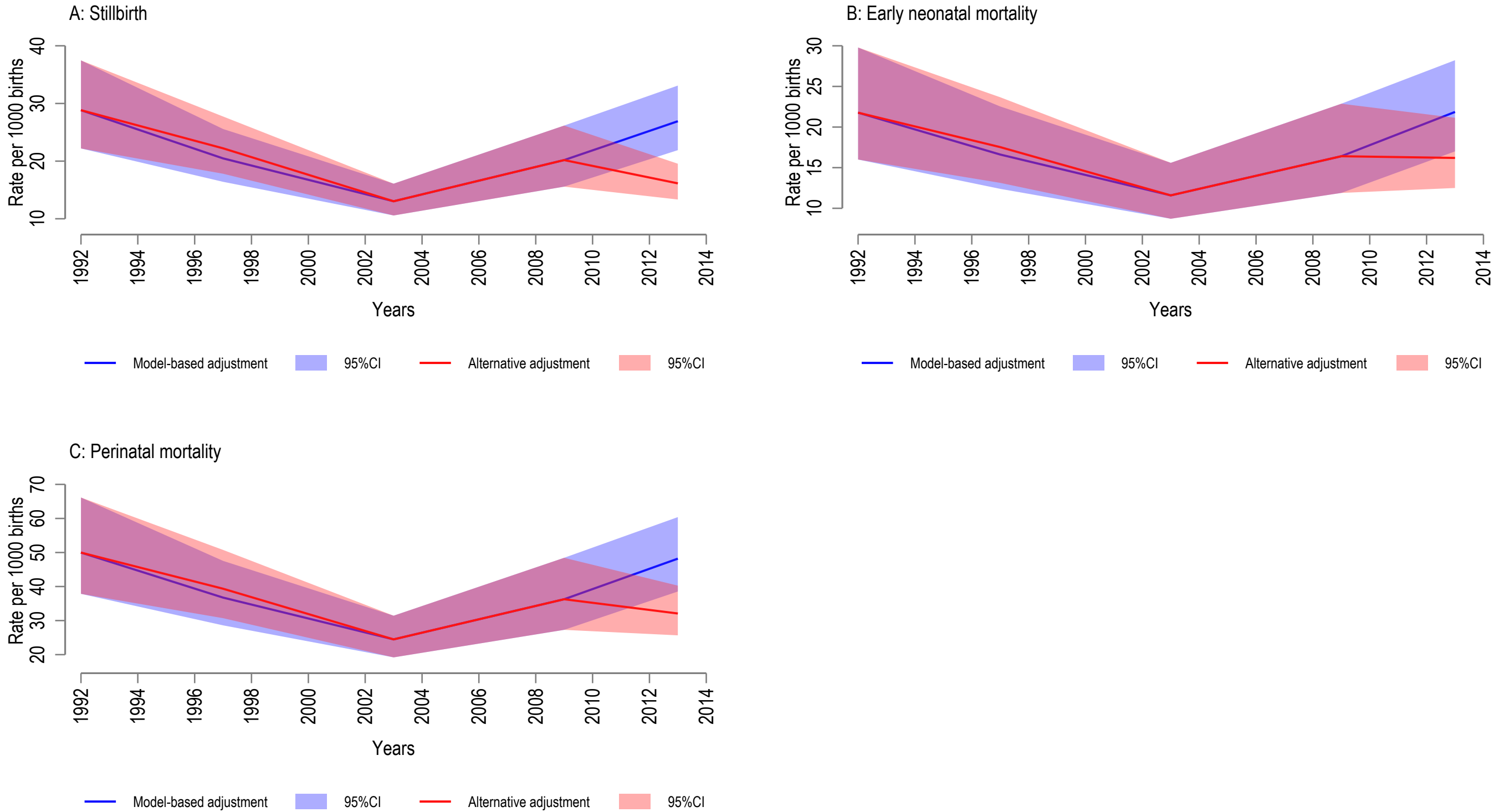

S1.12 Fig: Sensitivity analysis: comapring model-based adjusted against alternative adjustment -- Egypt

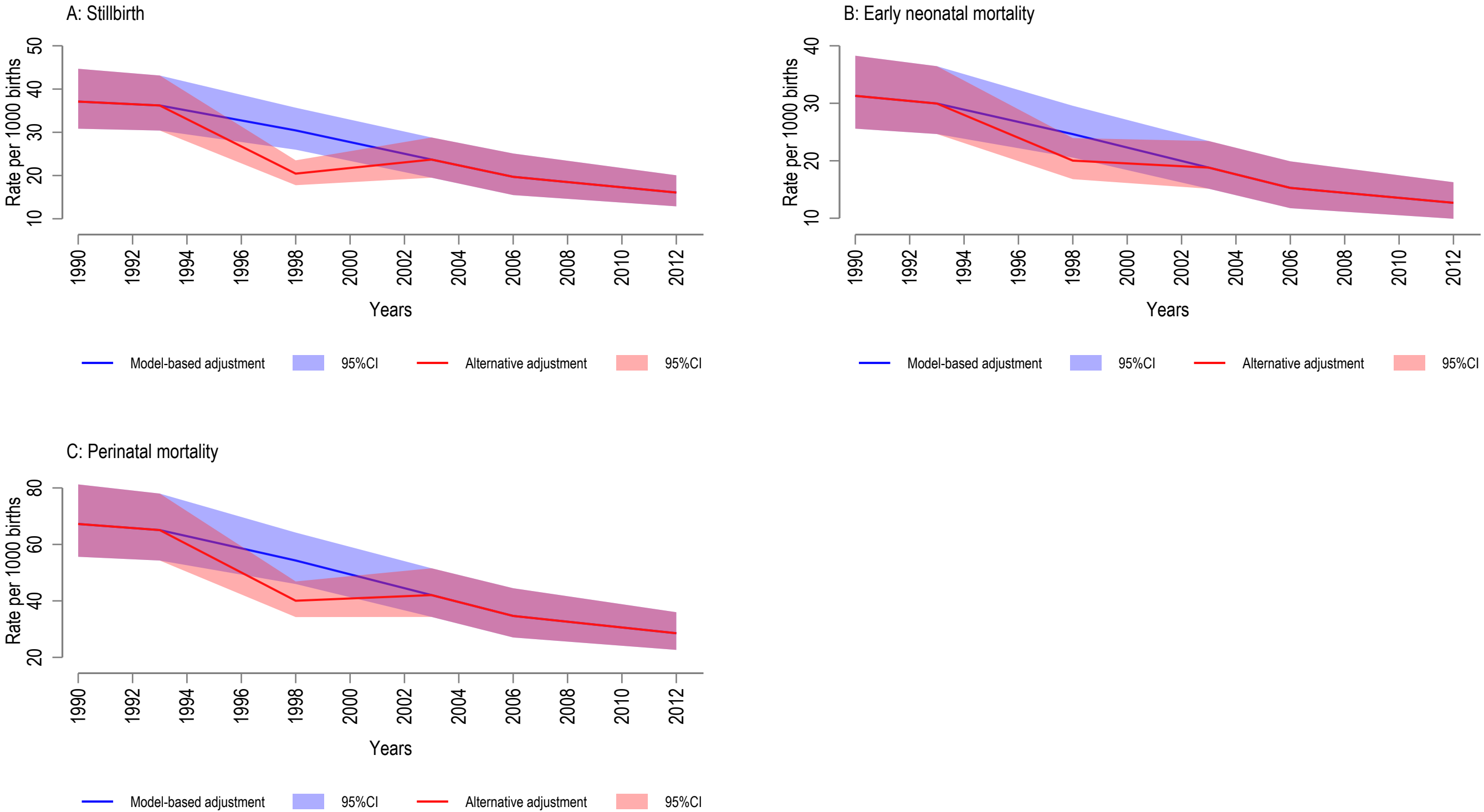

S1.13 Fig: Sensitivity analysis: comapring model-based adjusted against alternative adjustment -- Jordan

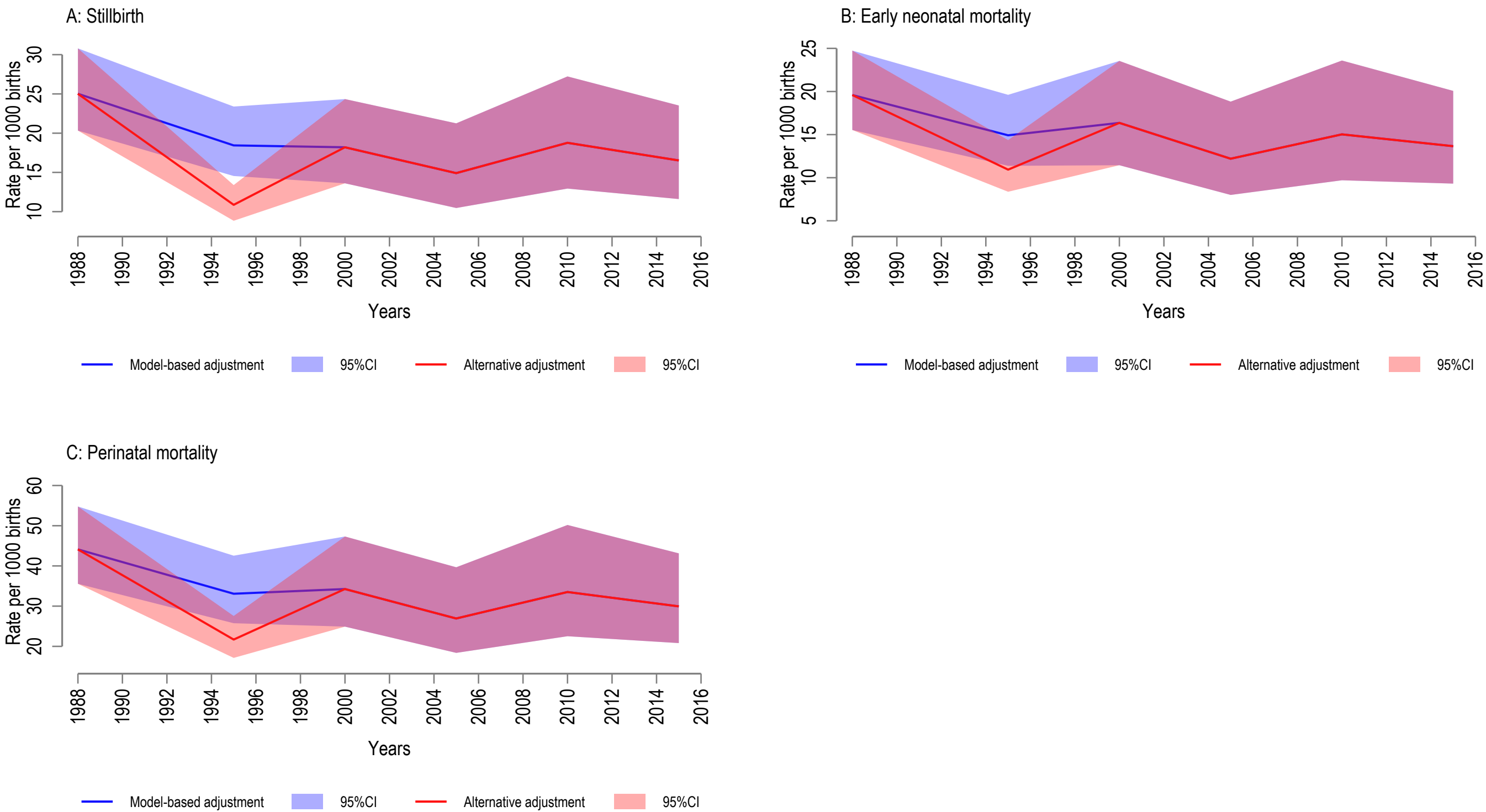

S1.14 Fig: Sensitivity analysis: comapring model-based adjusted against alternative adjustment -- Bangladesh

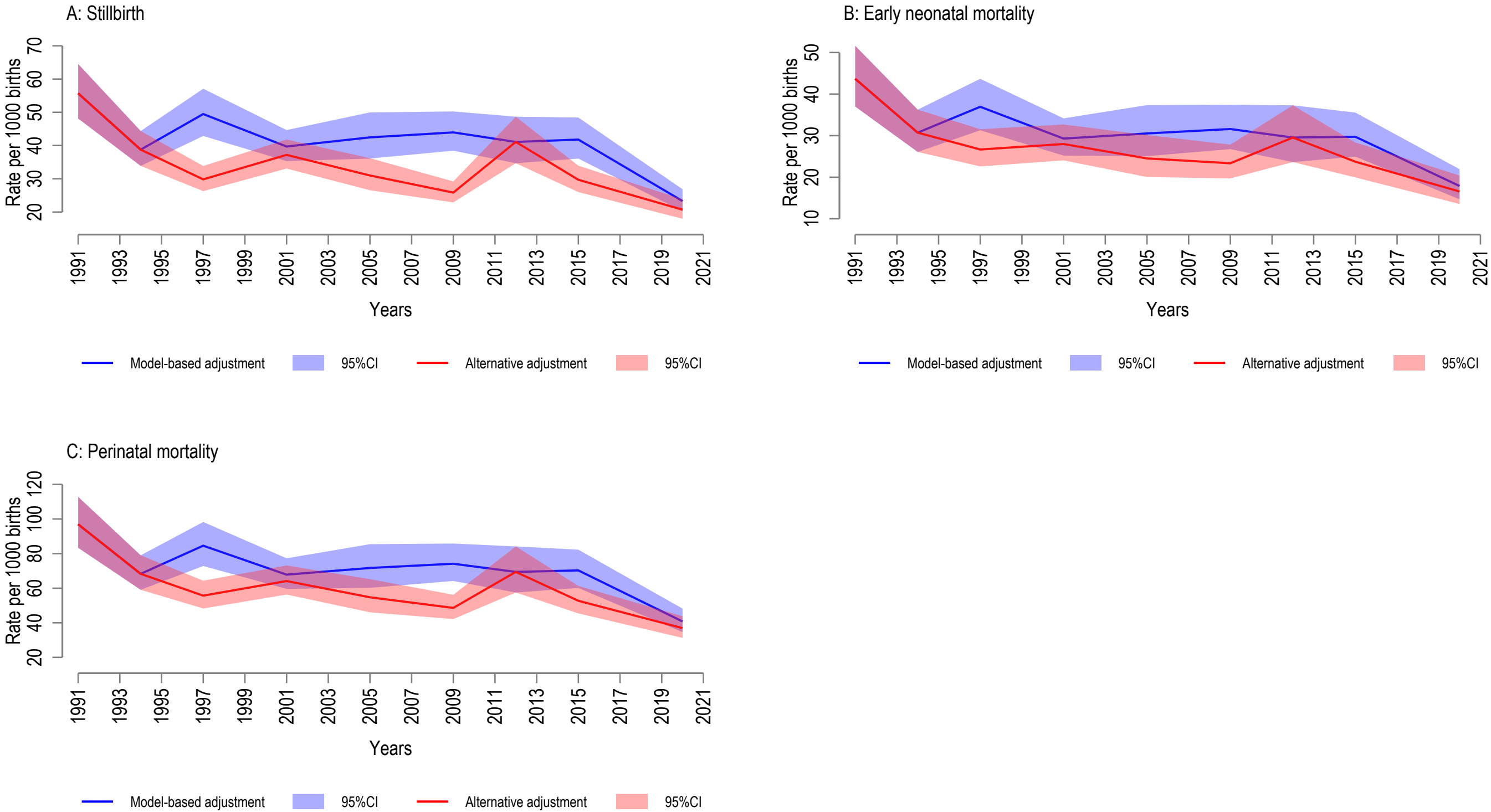

S1.15 Fig: Sensitivity analysis: comapring model-based adjusted against alternative adjustment -- Indonesia

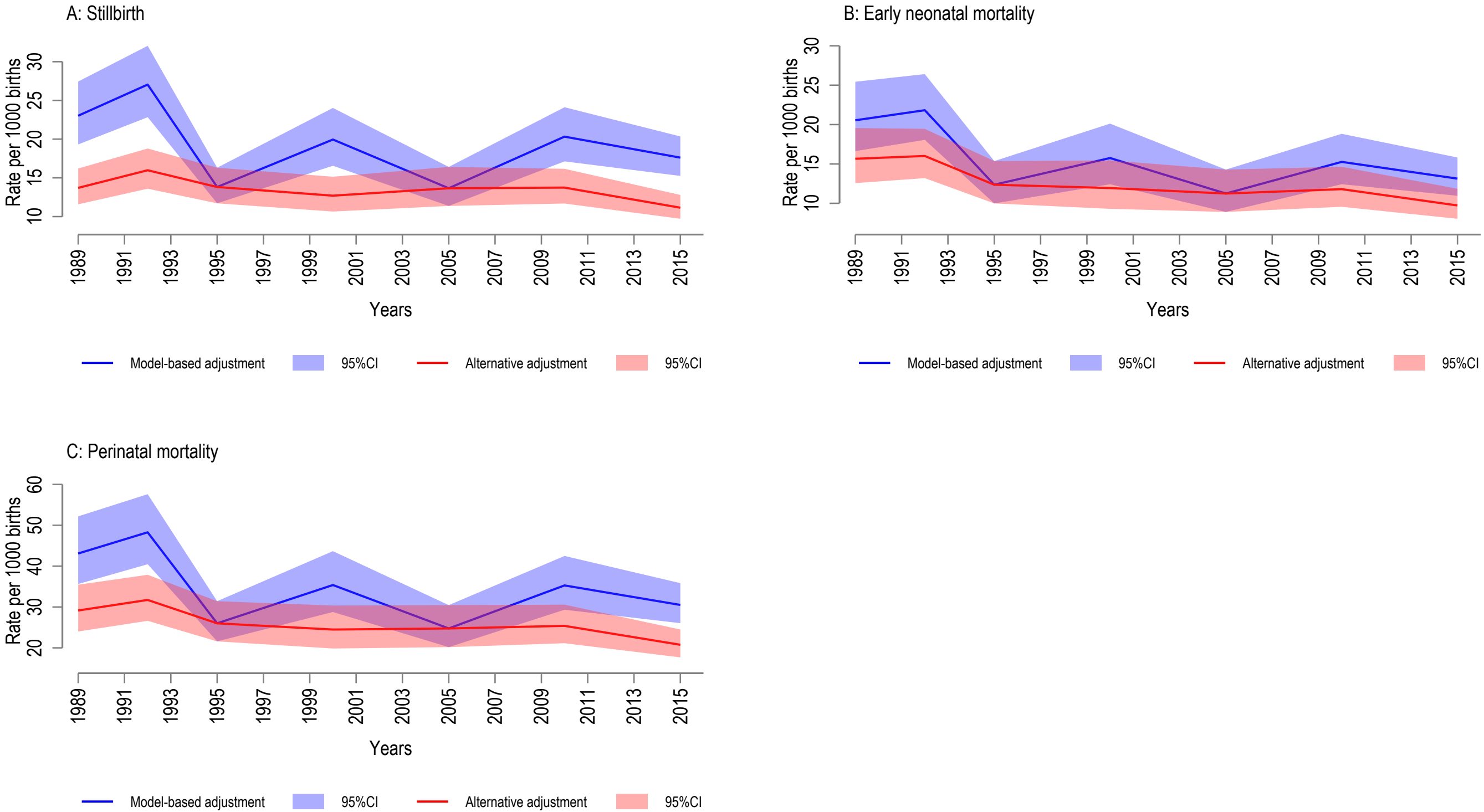

S1.16 Fig: Sensitivity analysis: comapring model-based adjusted against alternative adjustment -- Colombia

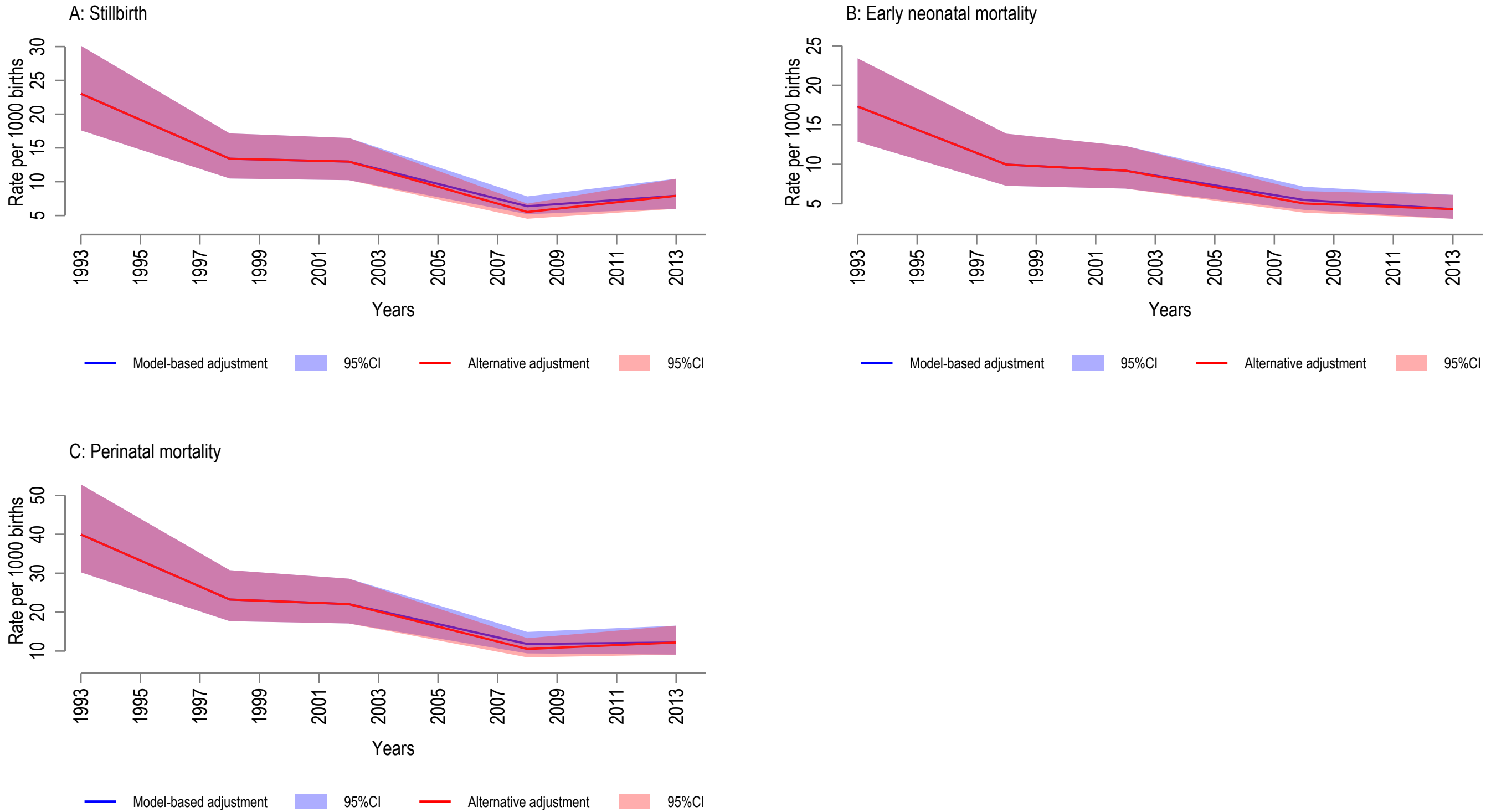

S1.17 Fig: Sensitivity analysis: comapring model-based adjusted against alternative adjustment -- Peru

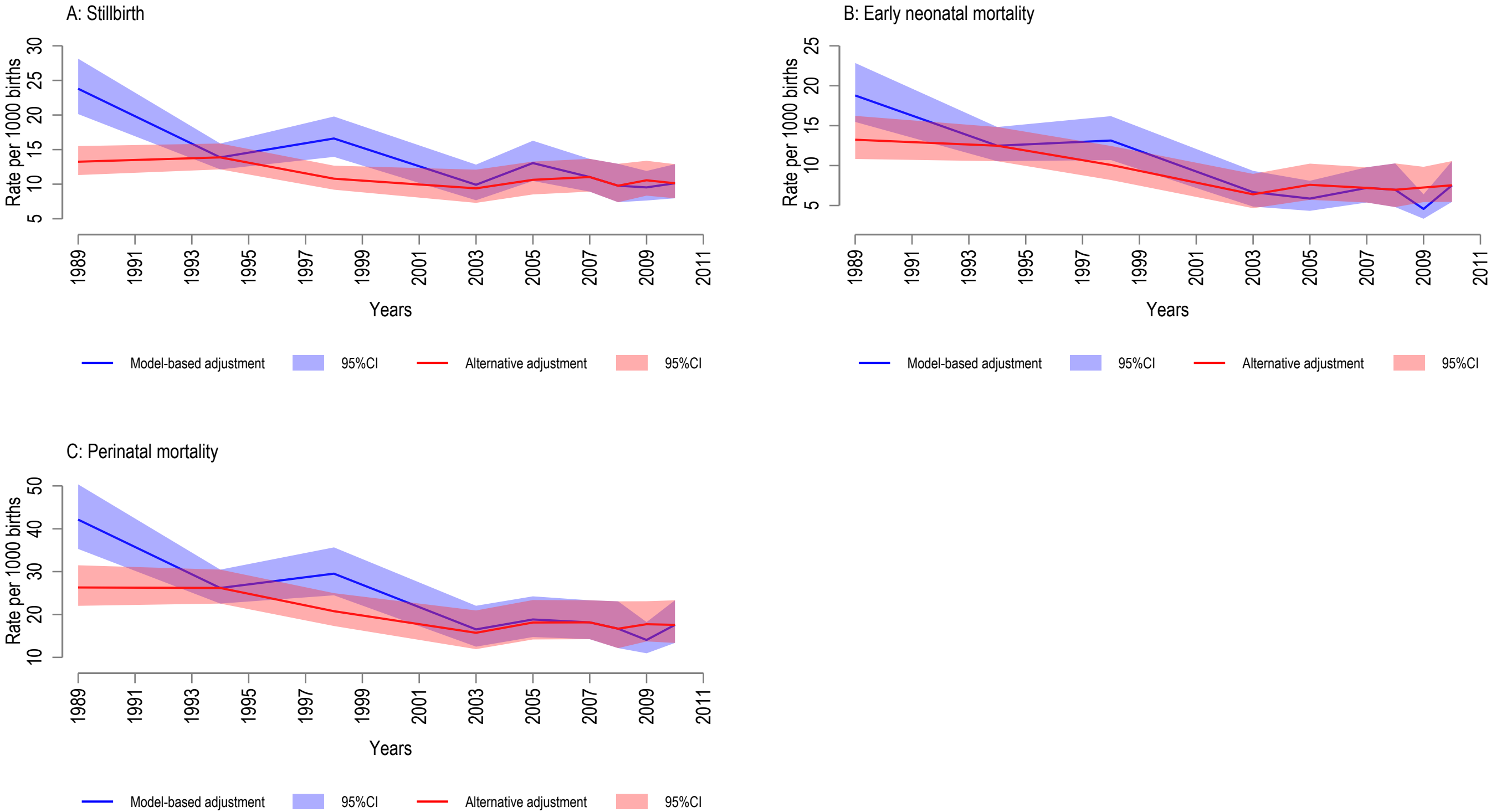

Supplement: Supplementary file 6 [file pmed.1004791.s006.pdf]
