# Supplementary material for: Trends of stillbirth, early neonatal and perinatal mortality rates and sociodemographic differentials of perinatal mortality rates in 17 low- and middle-income countries: A modeling study
Source: PLoS Med. 2026 Aug 24;23(8):e1004791. doi: 10.1371/journal.pmed.1004791 (PMC13502698; doi:10.1371/journal.pmed.1004791)
Supplement: Supplementary file 7 [file pmed.1004791.s007.pdf]

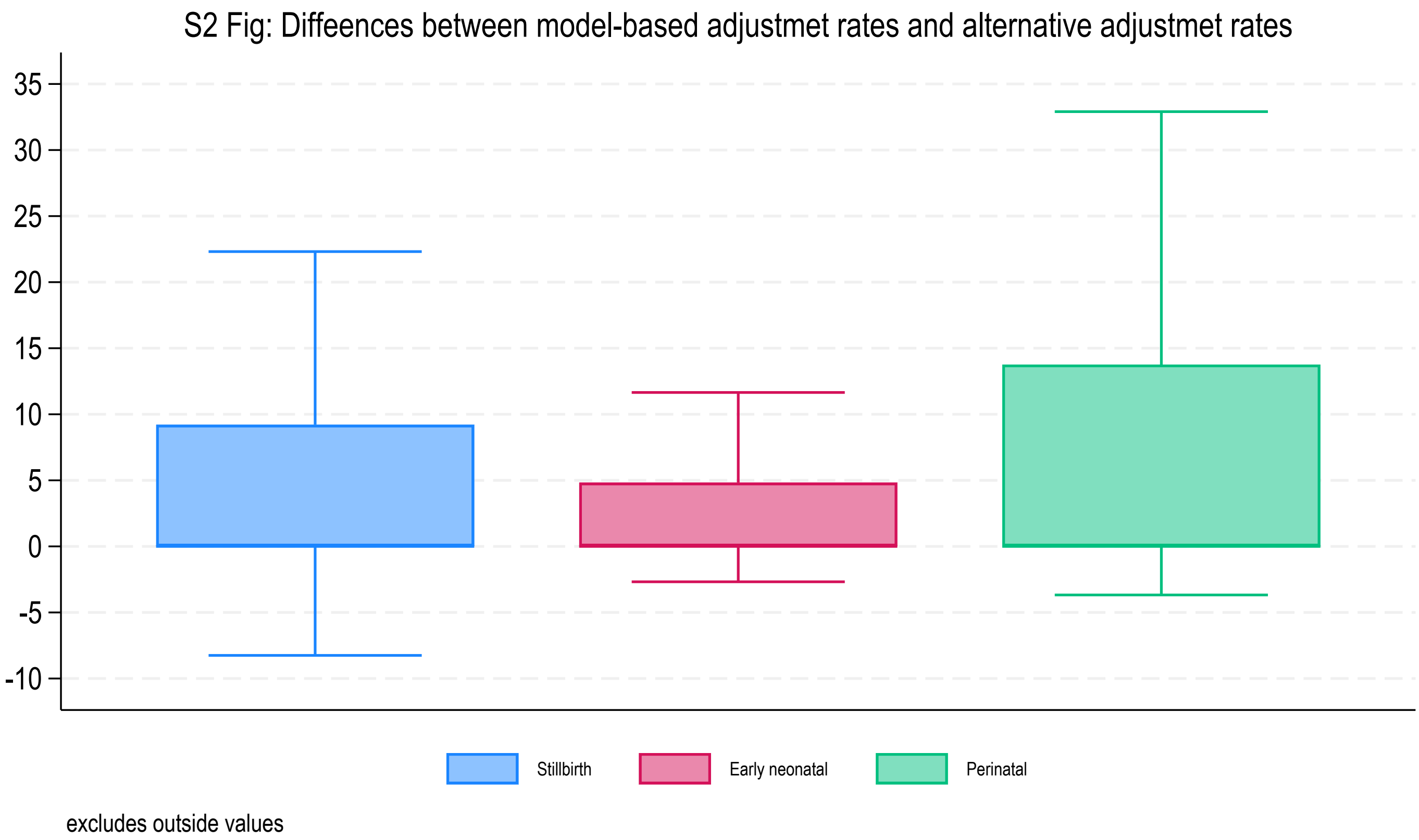
S2 Fig: Diffeences between model-based adjustmet rates and alternative adjustmet rates
35
30
25
20
15
10
5
0
-5
-10
Stillbirth
Early neonatal
Perinatal
excludes outside values
