# Supplementary material for: Trends of stillbirth, early neonatal and perinatal mortality rates and sociodemographic differentials of perinatal mortality rates in 17 low- and middle-income countries: A modeling study
Source: PLoS Med. 2026 Aug 24;23(8):e1004791. doi: 10.1371/journal.pmed.1004791 (PMC13502698; doi:10.1371/journal.pmed.1004791)
Supplement: Supplementary file 8 [file pmed.1004791.s008.pdf]

S3.1 Fig: Trends in Stillbirth, Early neonatal and Perinatal mortality -- Ghana

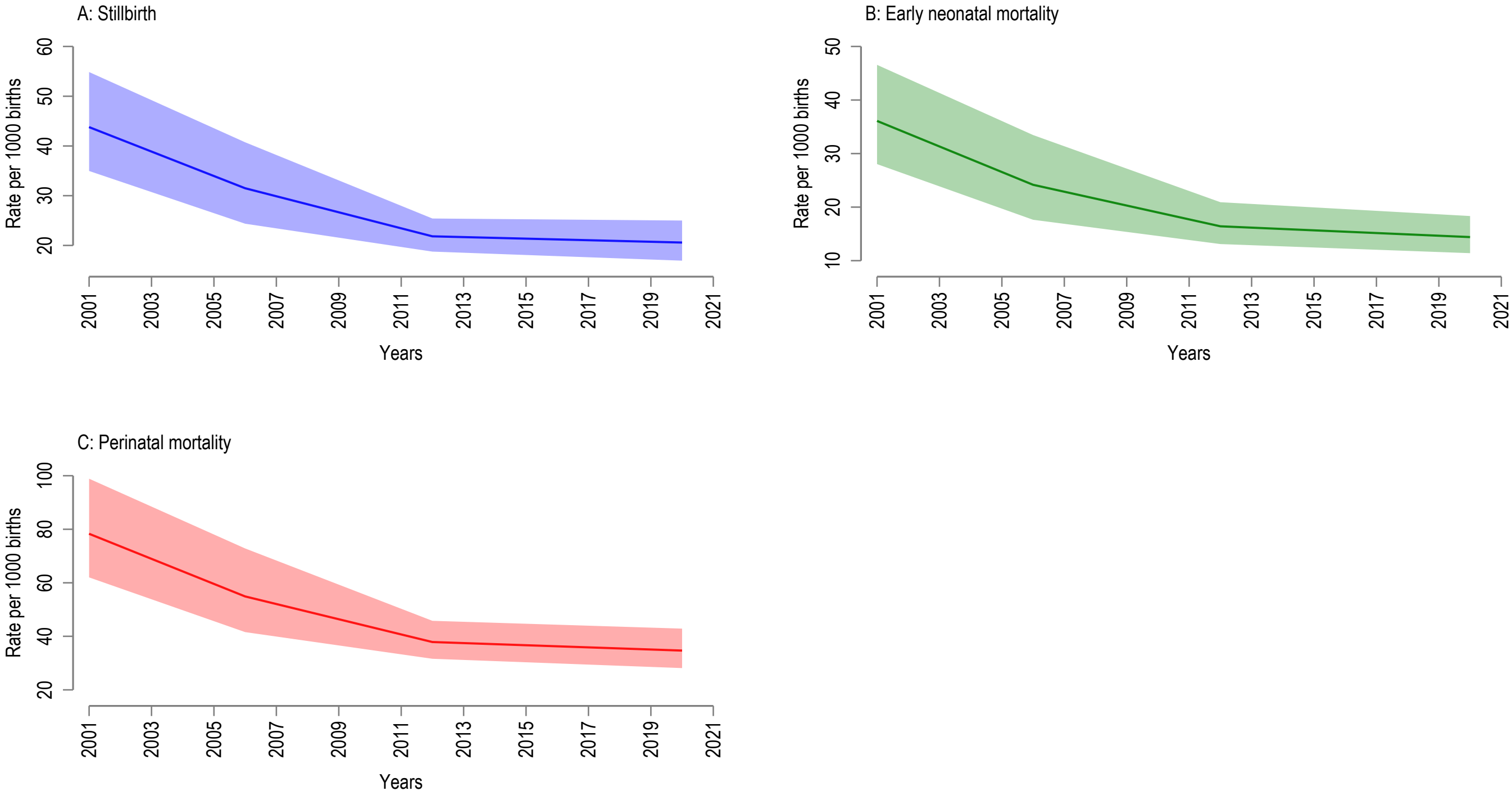

These are adjsted estimates

S3.2 Fig: Trends in Stillbirth, Early neonatal and Perinatal mortality -- Kenya

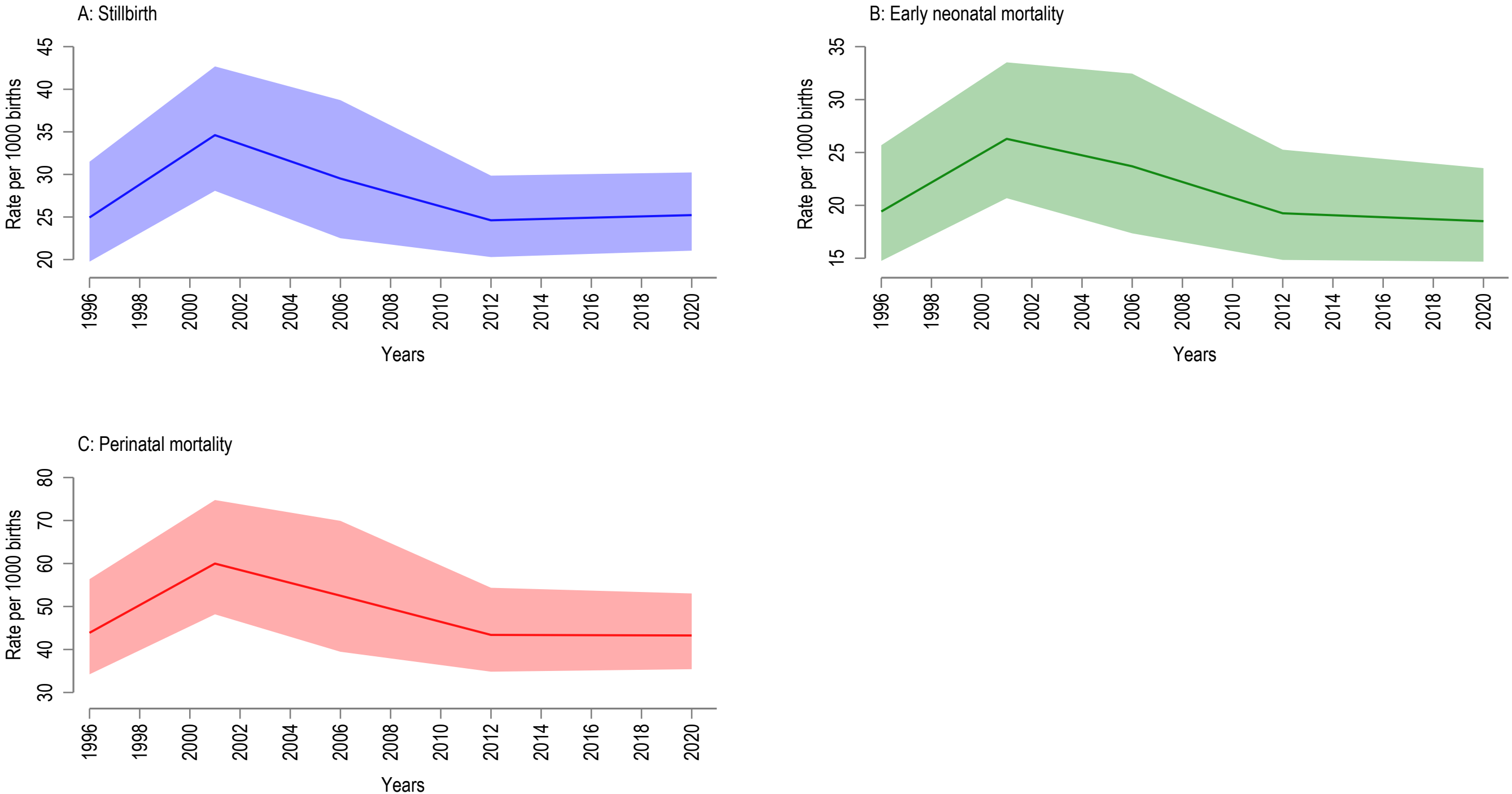

These are adjsted estimates

S3.3 Fig: Trends in Stillbirth, Early neonatal and Perinatal mortality -- Malawi

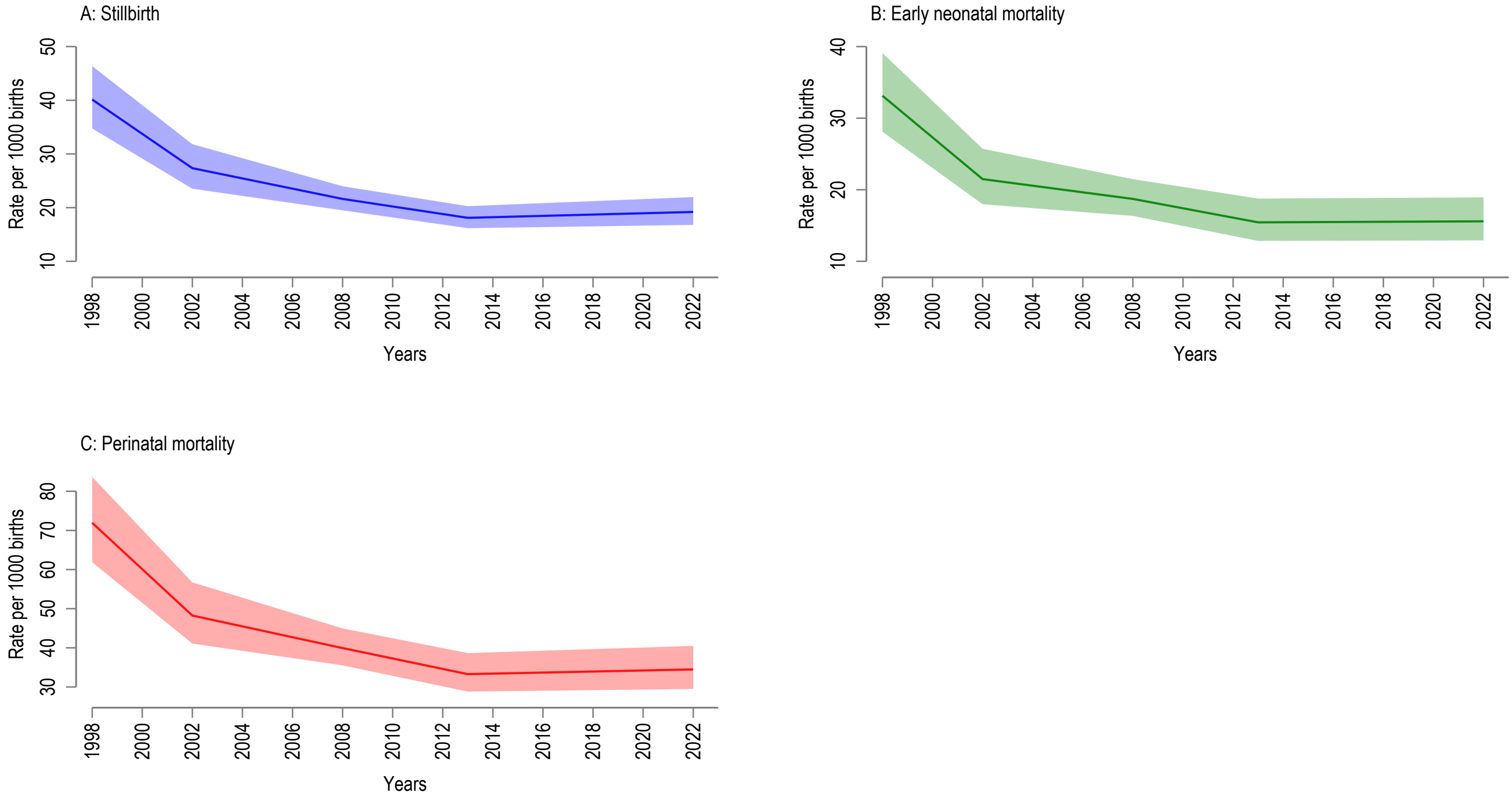

These are adjsted estimates

S3.4 Fig: Trends in Stillbirth, Early neonatal and Perinatal mortality -- Mali

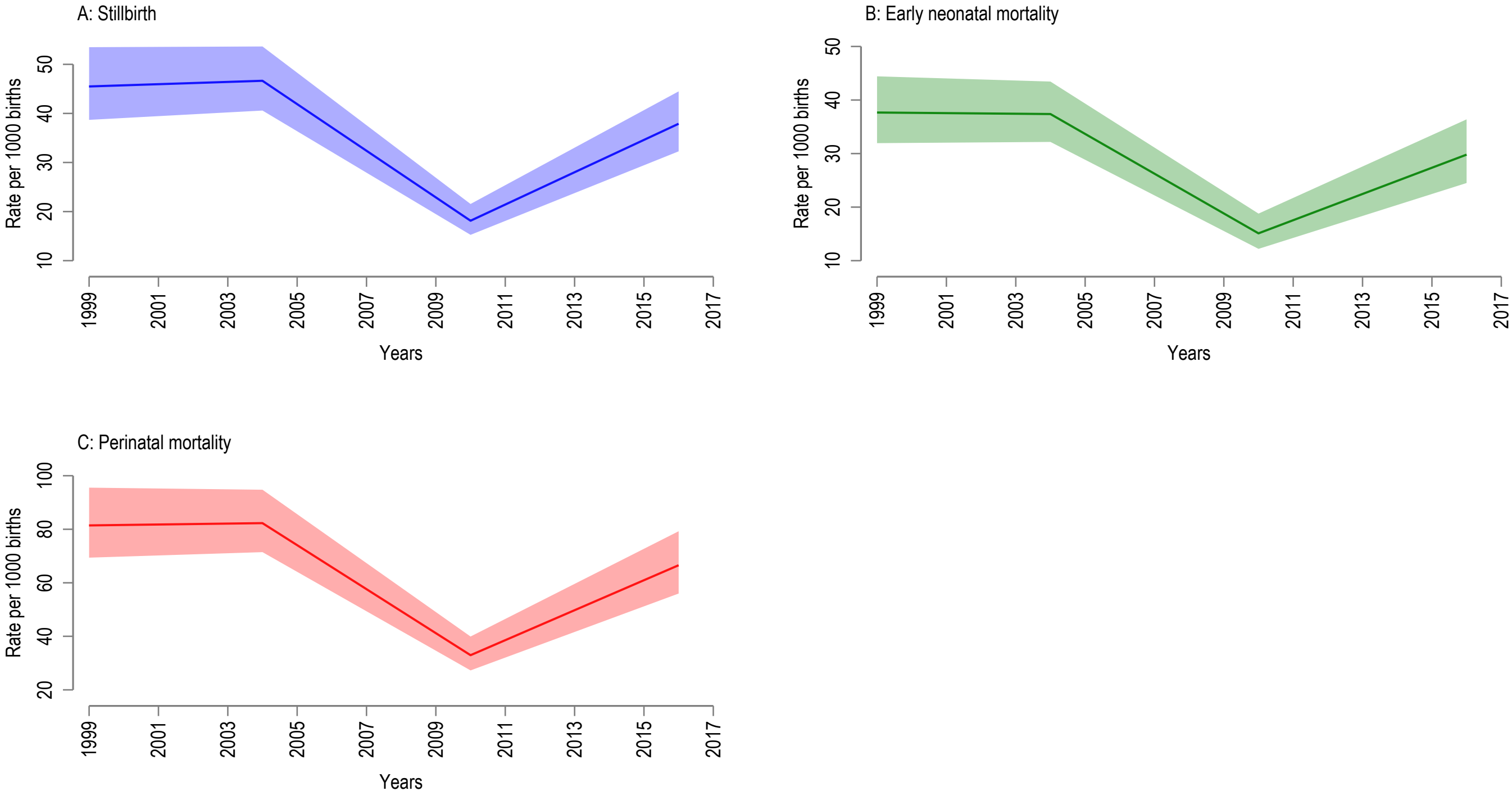

These are adjsted estimates

S3.5 Fig: Trends in Stillbirth, Early neonatal and Perinatal mortality -- Nigeria

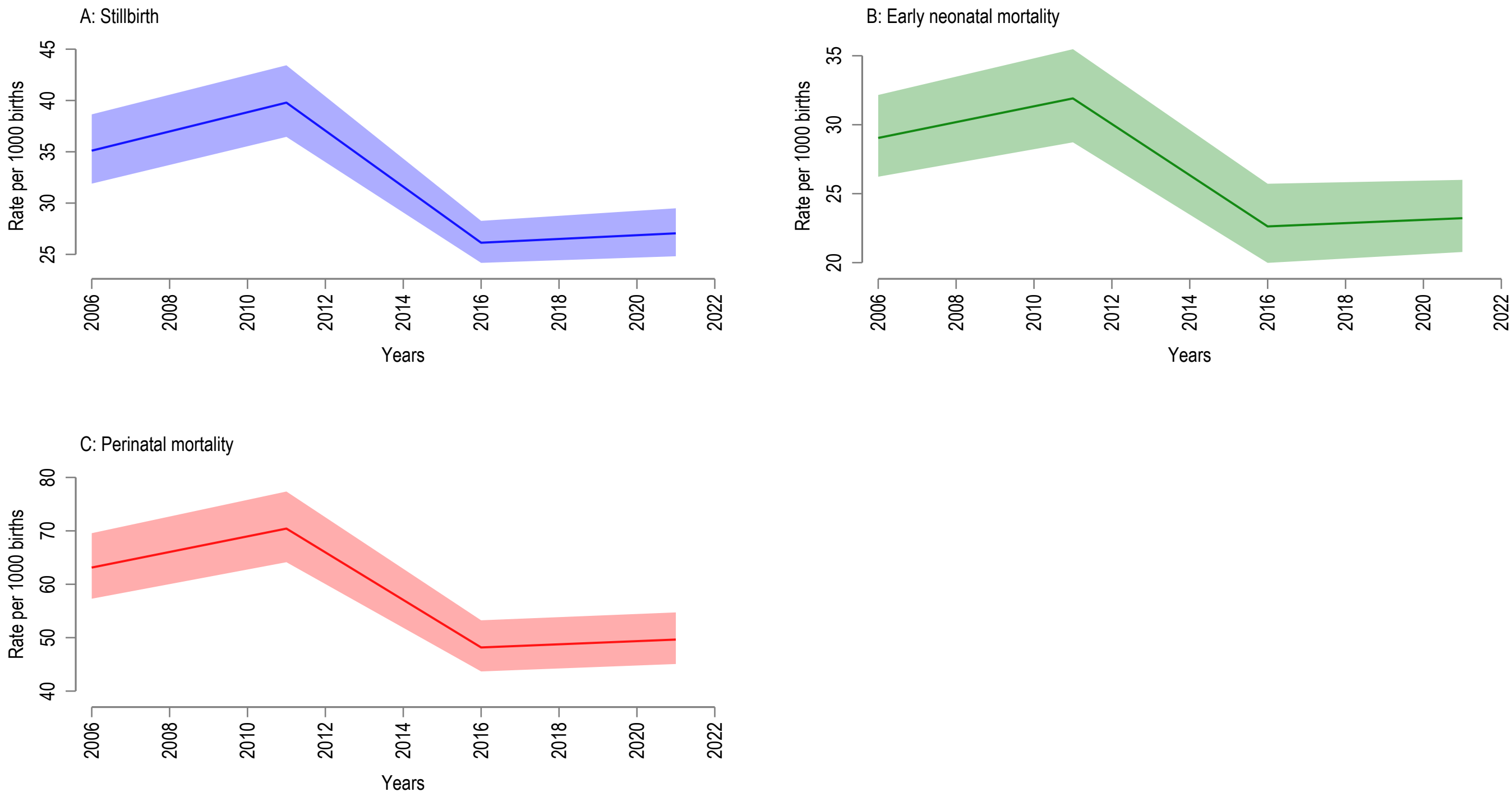

These are adjsted estimates

S3.6 Fig: Trends in Stillbirth, Early neonatal and Perinatal mortality -- Rwanda

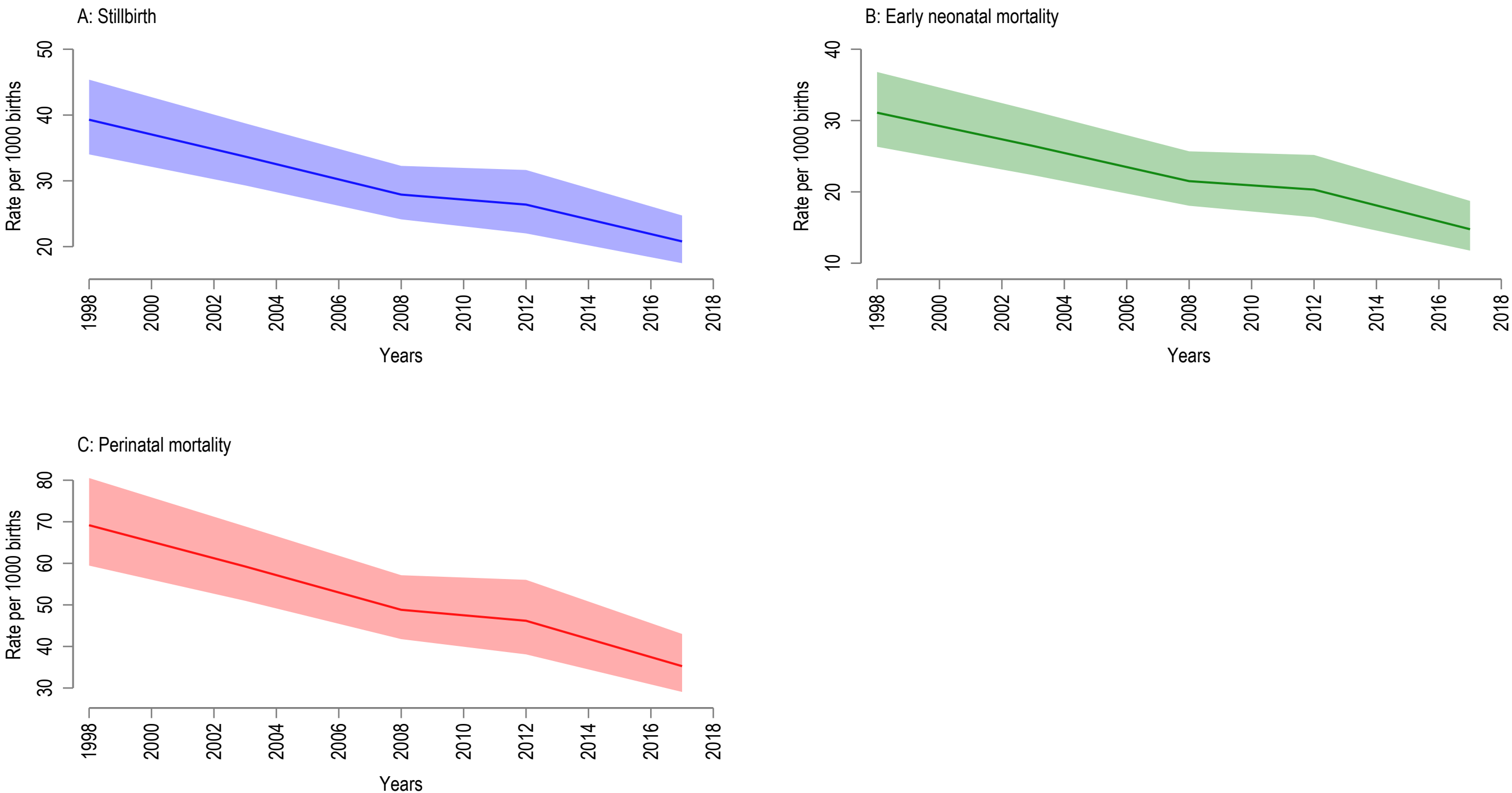

These are adjsted estimates

S3.7 Fig: Trends in Stillbirth, Early neonatal and Perinatal mortality -- Senegal

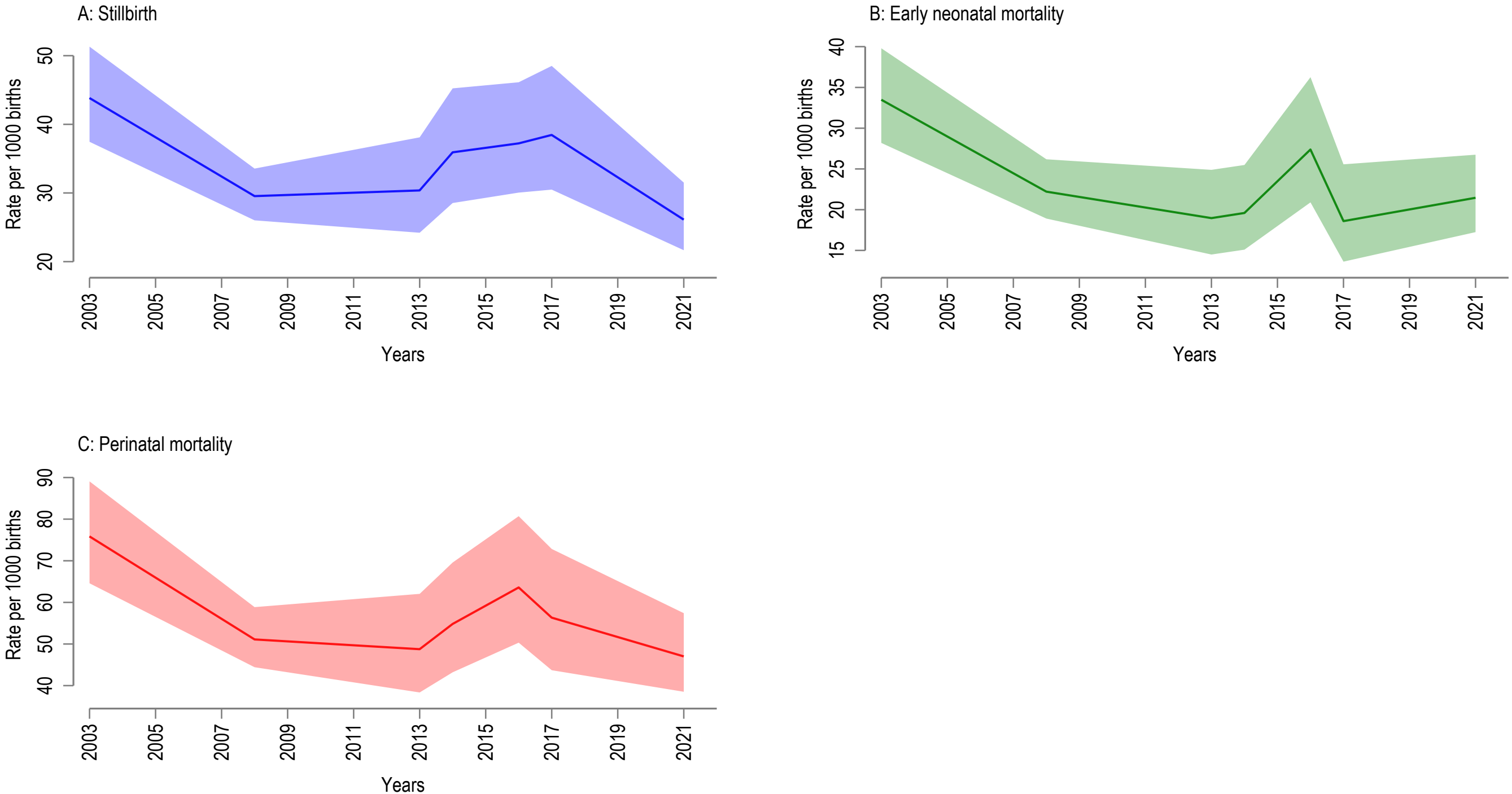

These are adjsted estimates

S3.8 Fig: Trends in Stillbirth, Early neonatal and Perinatal mortality -- Tanzania

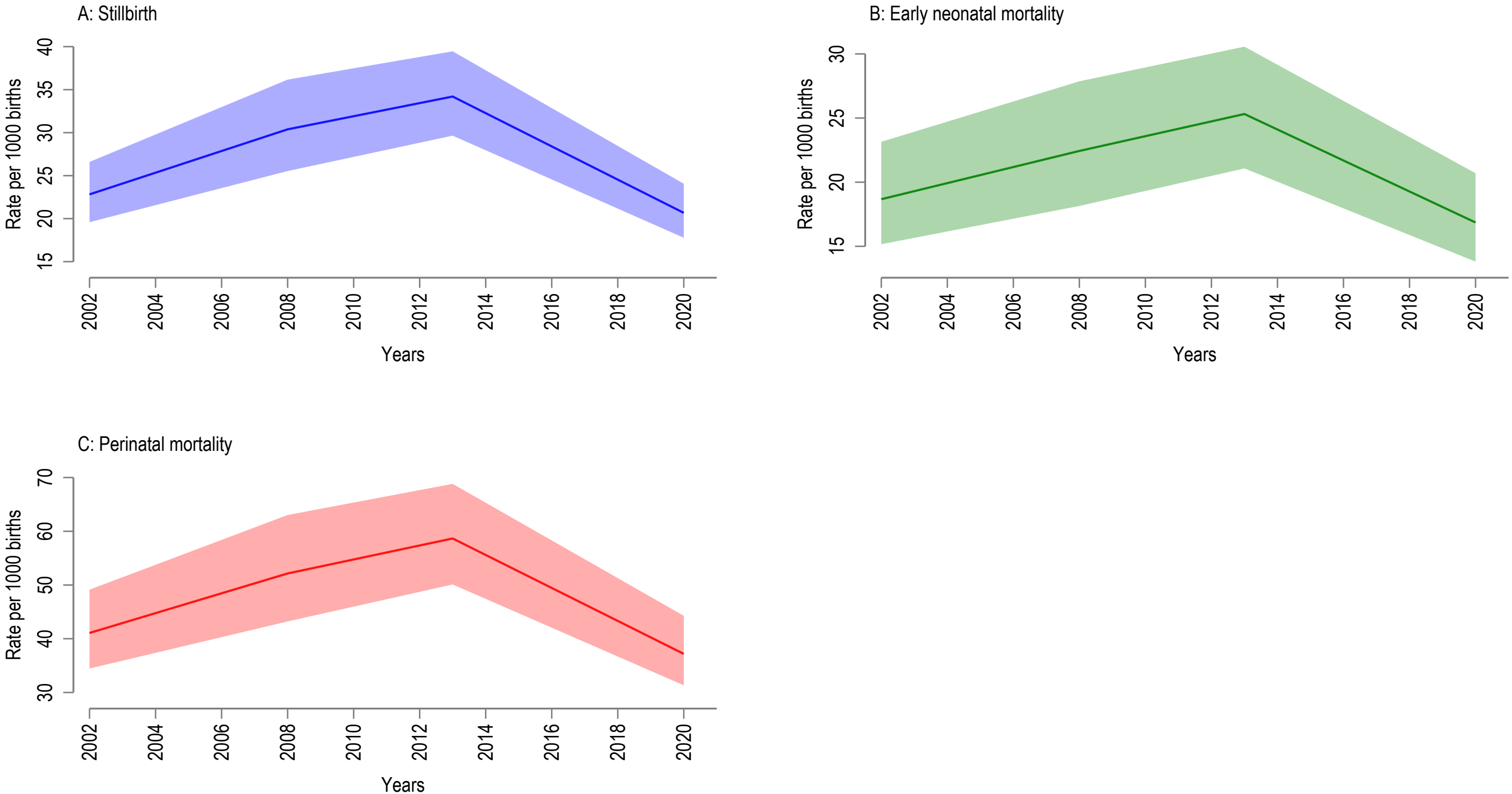

These are adjsted estimates

S3.9 Fig: Trends in Stillbirth, Early neonatal and Perinatal mortality -- Uganda

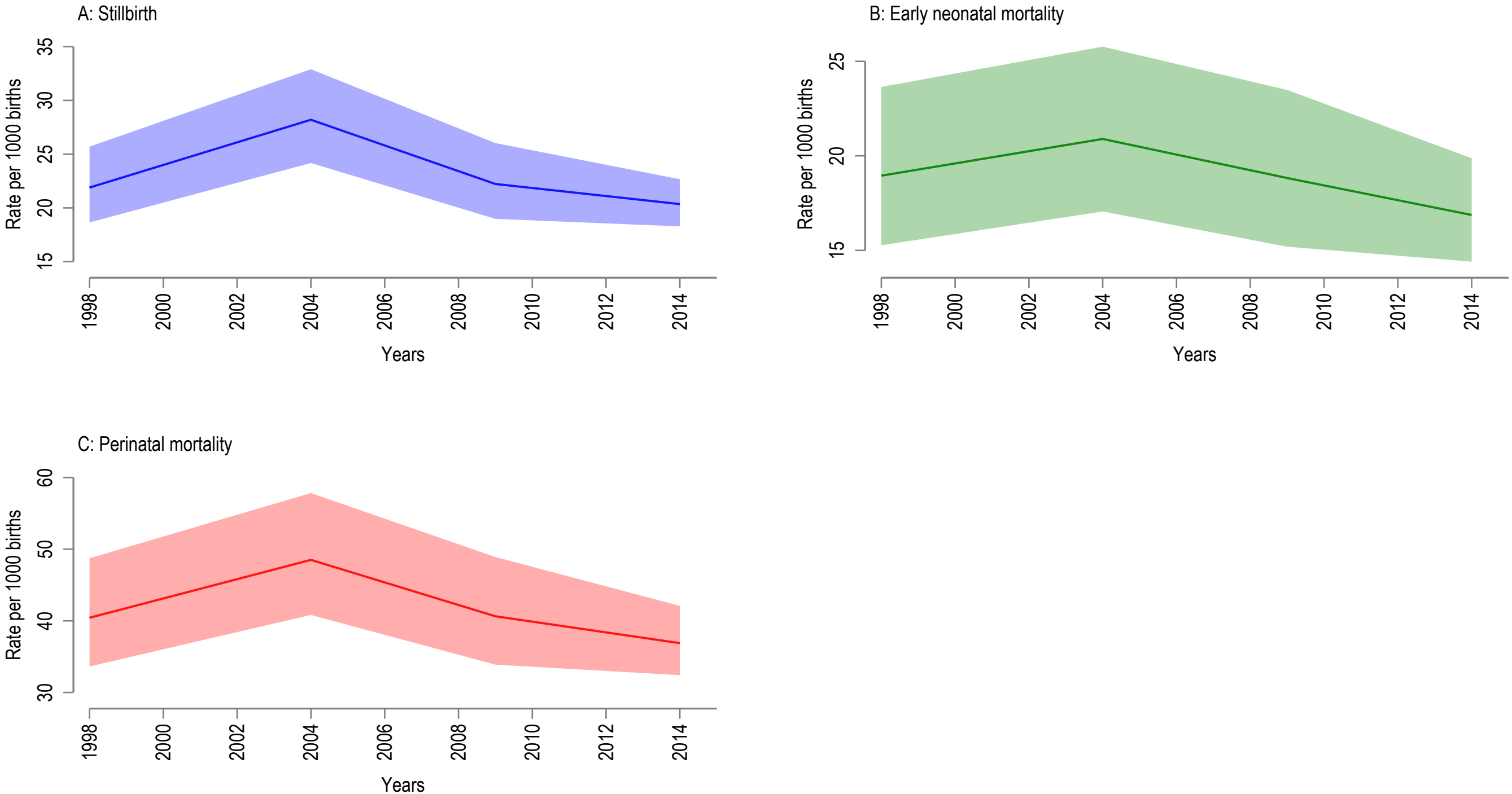

These are adjsted estimates

S3.10 Fig: Trends in Stillbirth, Early neonatal and Perinatal mortality -- Zambia

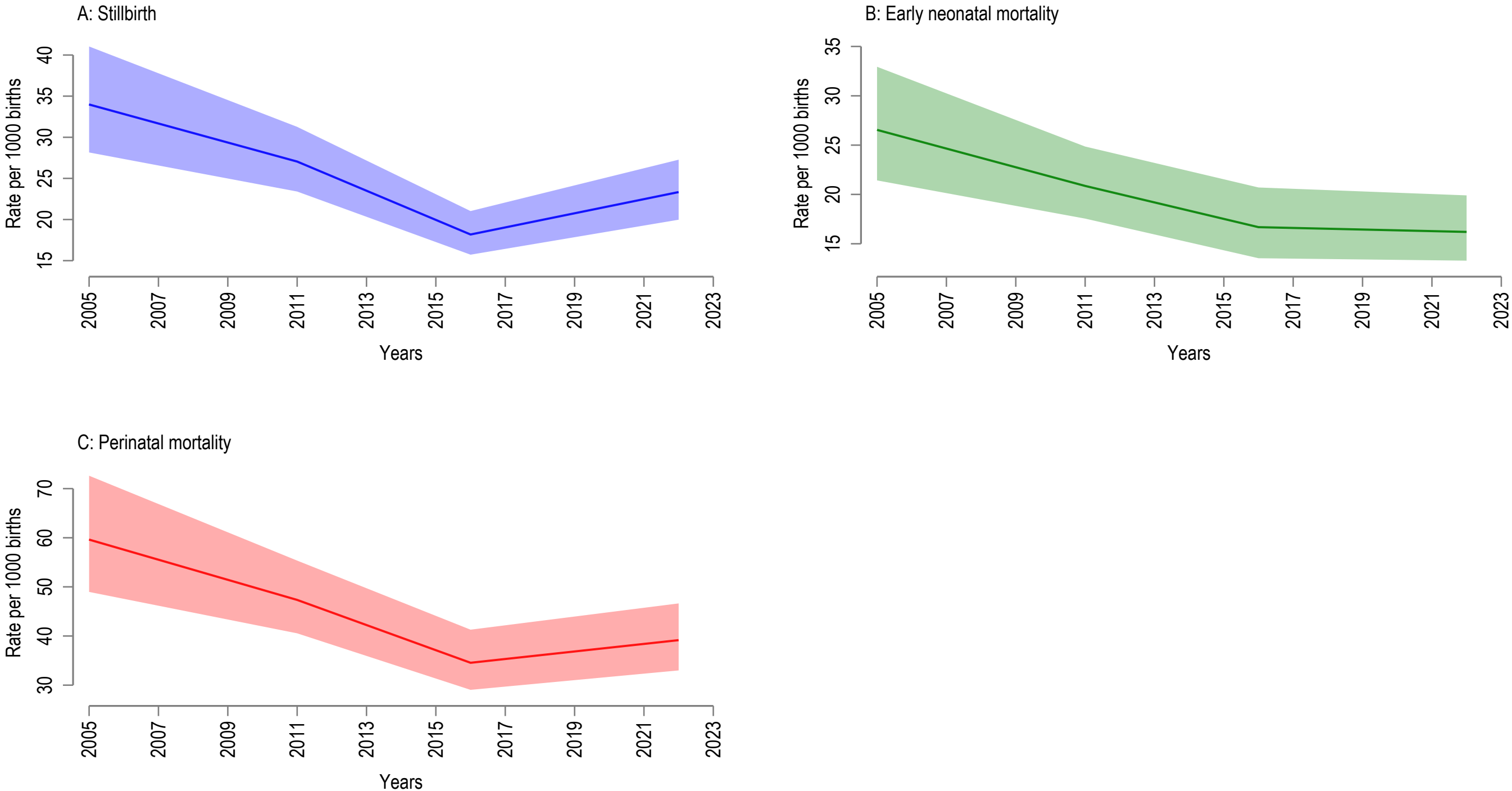

These are adjsted estimates

S3.11 Fig: Trends in Stillbirth, Early neonatal and Perinatal mortality -- Zimbabwe

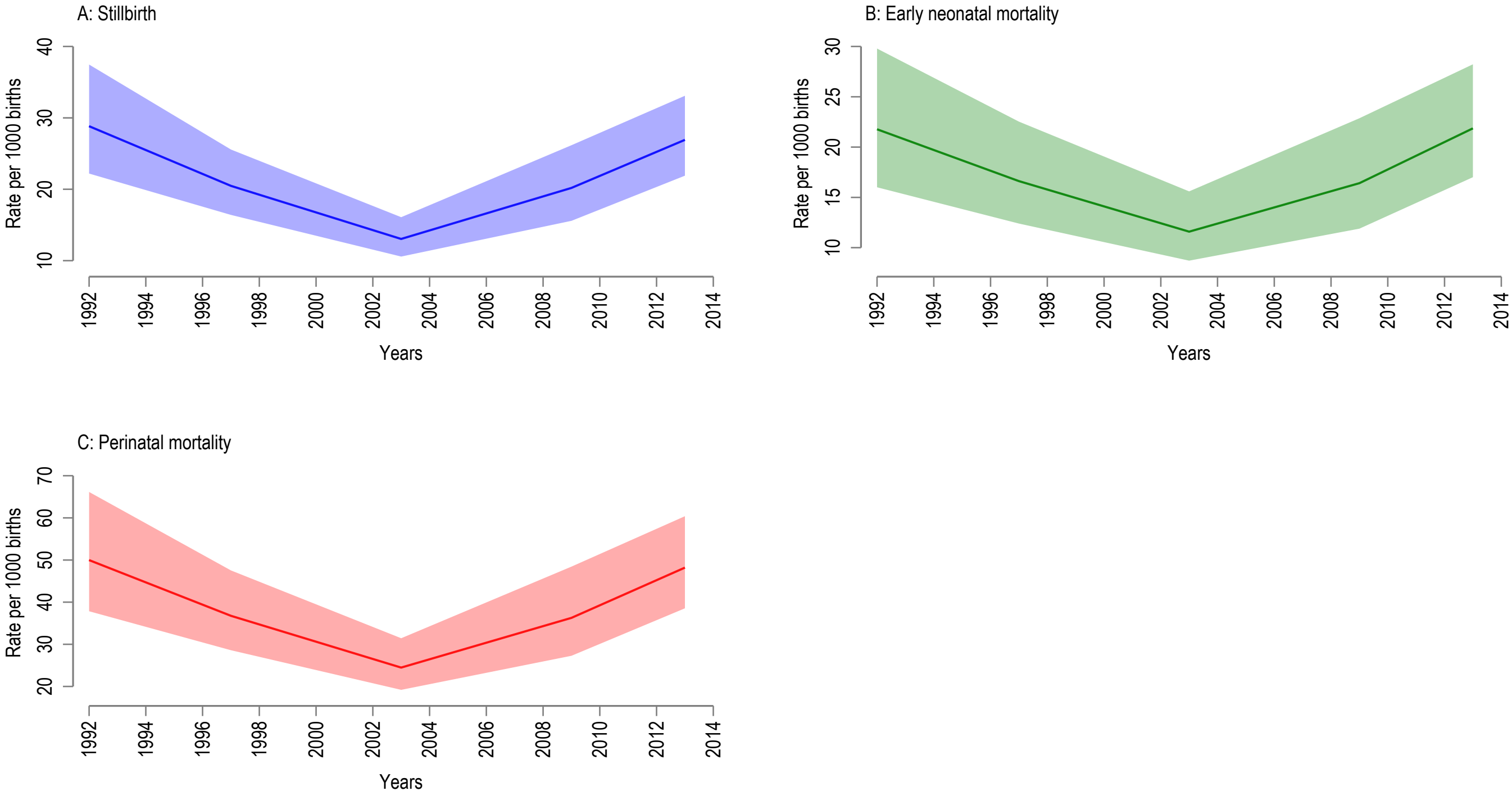

These are adjsted estimates

S3.12 Fig: Trends in Stillbirth, Early neonatal and Perinatal mortality -- Egypt

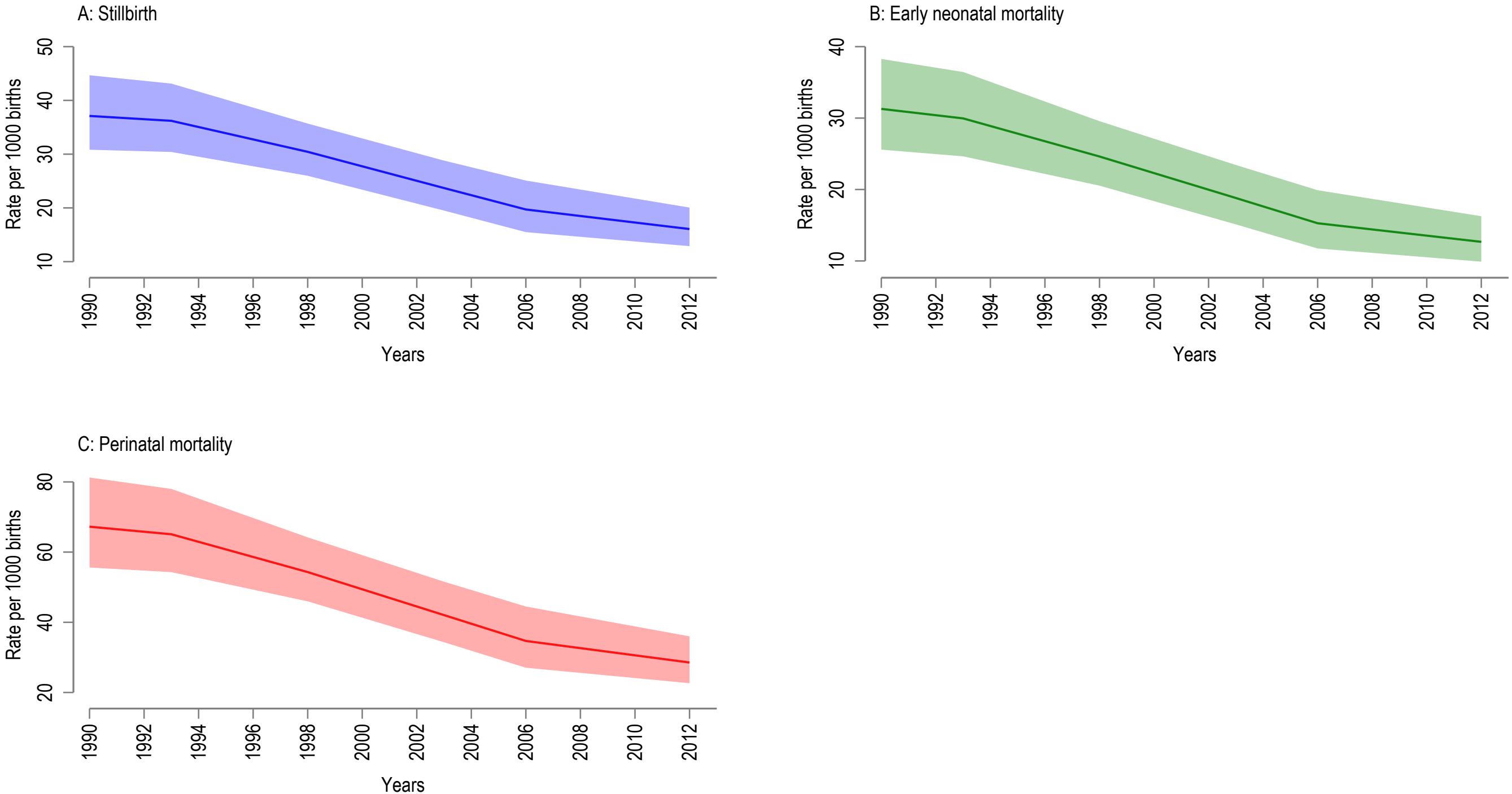

These are adjsted estimates

S3.13 Fig: Trends in Stillbirth, Early neonatal and Perinatal mortality -- Jordan

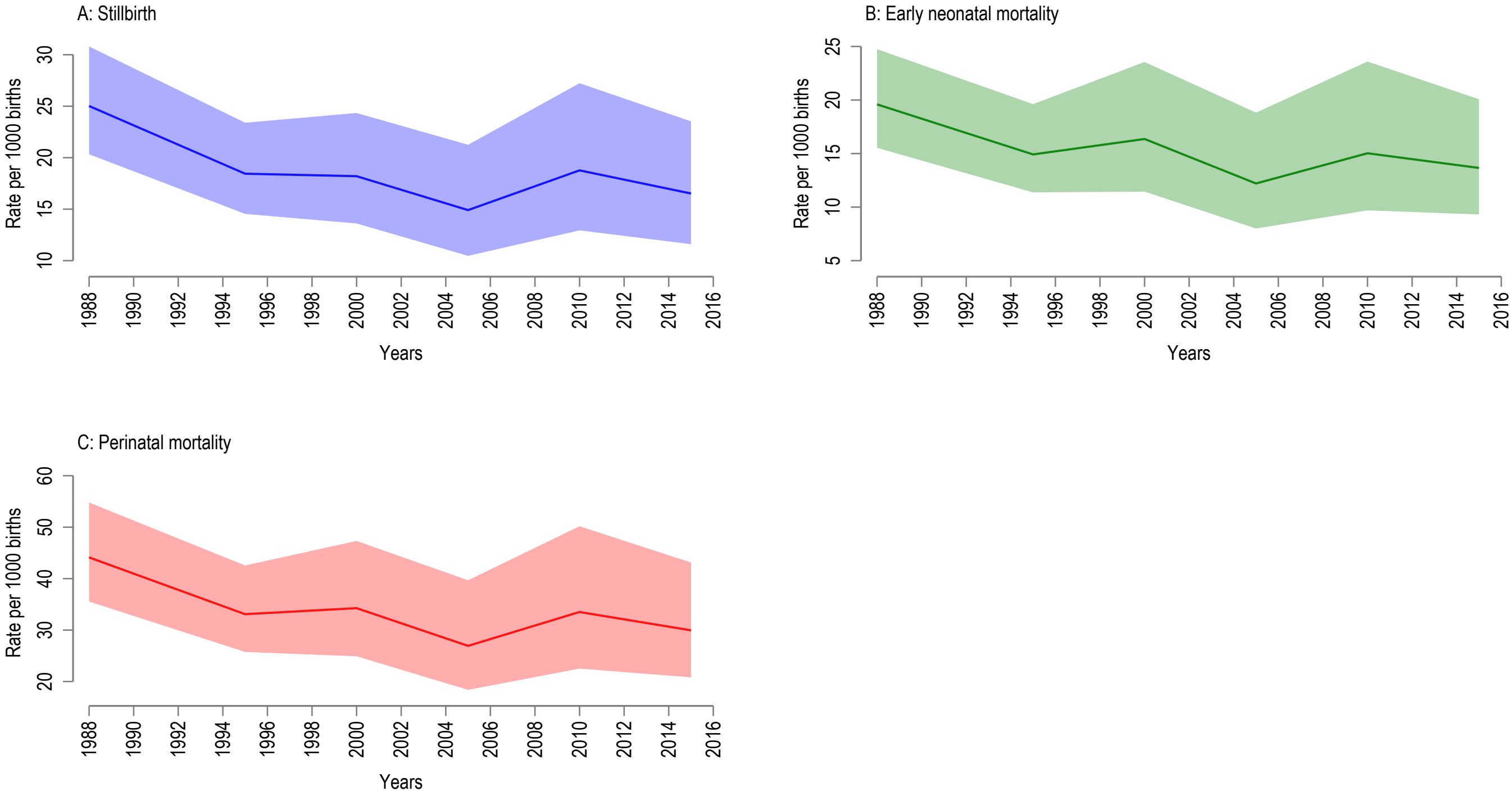

These are adjsted estimates

S3.14 Fig: Trends in Stillbirth, Early neonatal and Perinatal mortality -- Bangladesh

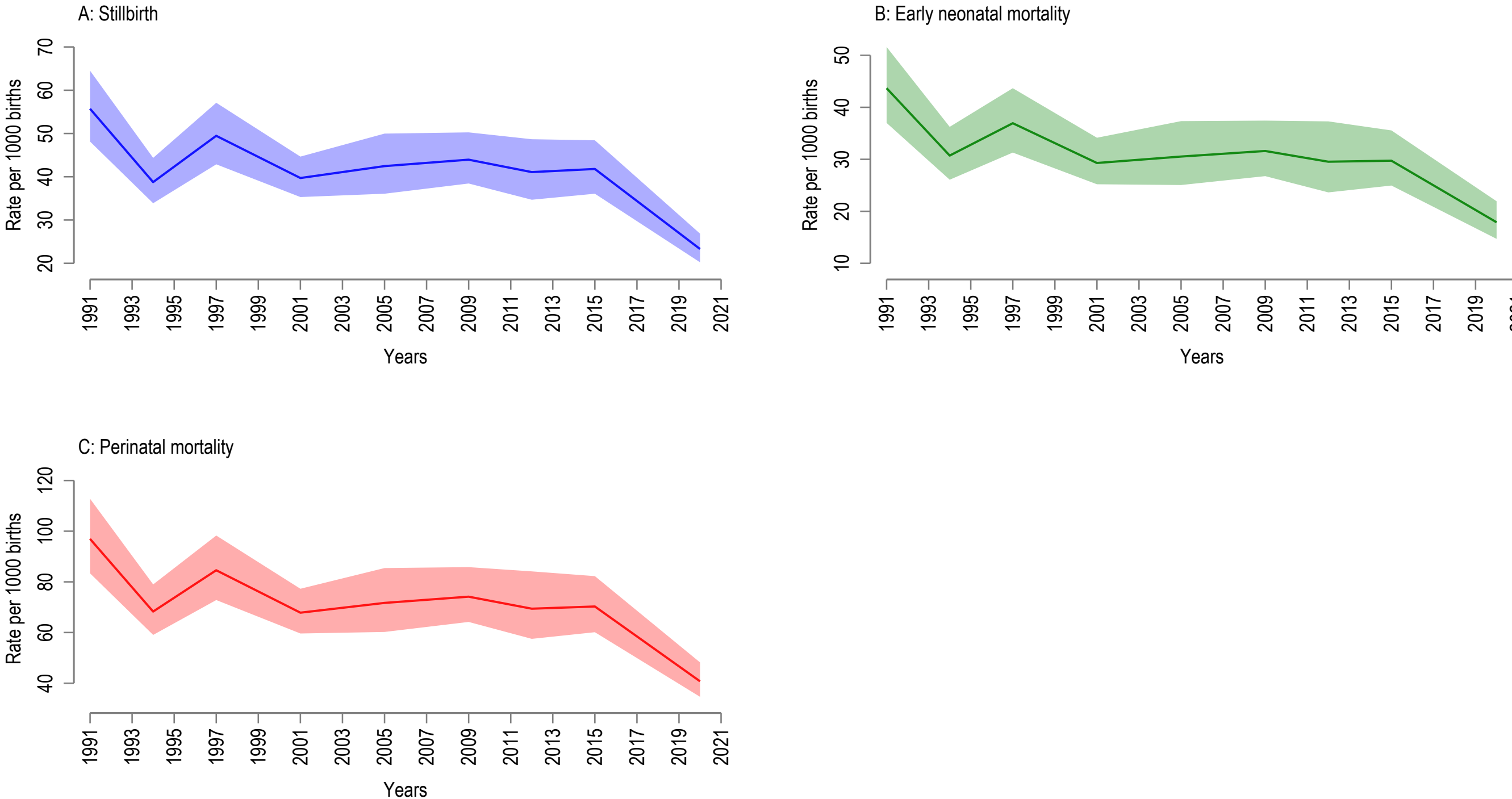

These are adjsted estimates

S3.15 Fig: Trends in Stillbirth, Early neonatal and Perinatal mortality -- Indonesia

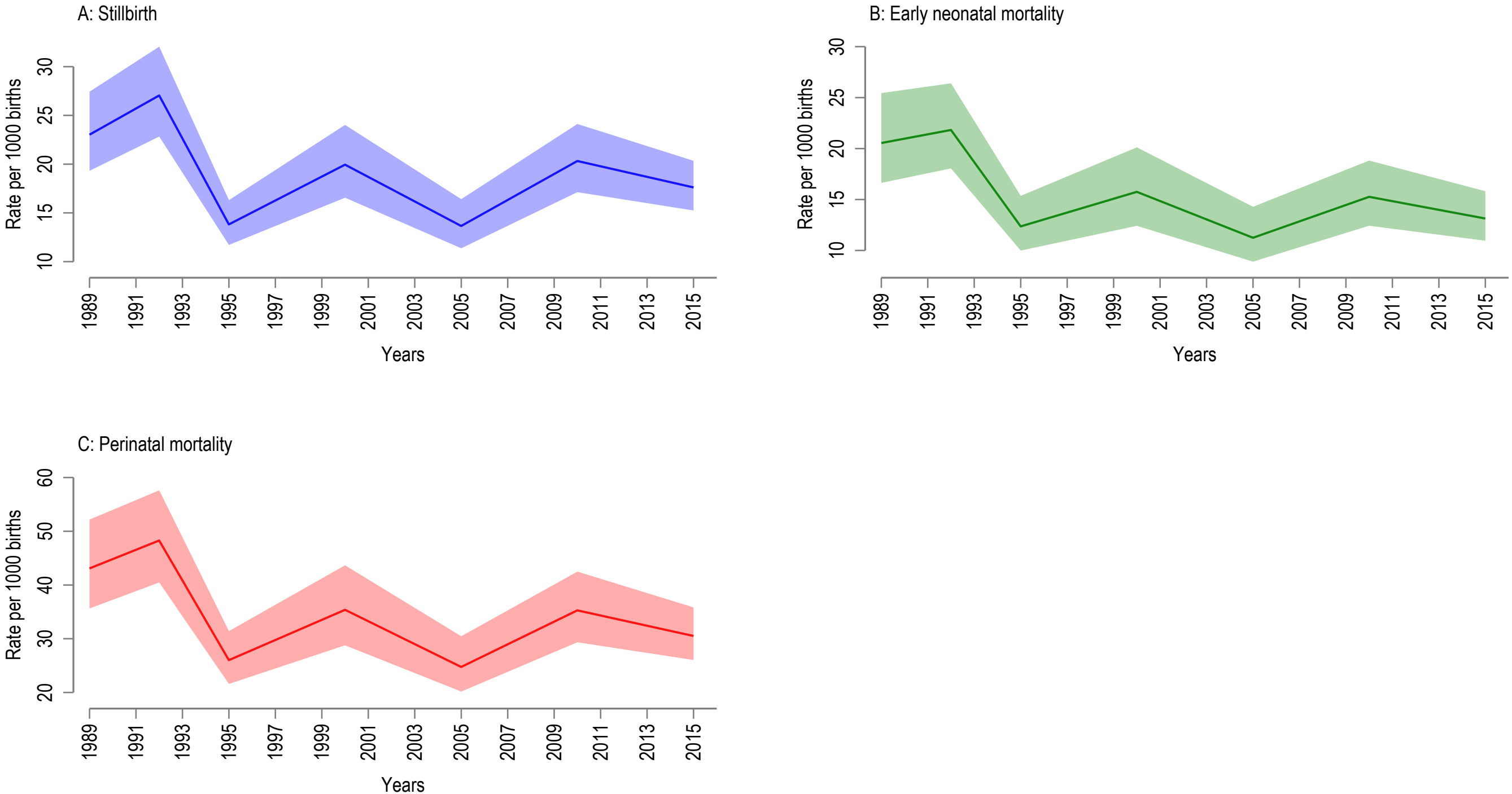

These are adjsted estimates

S3.16 Fig: Trends in Stillbirth, Early neonatal and Perinatal mortality -- Colombia

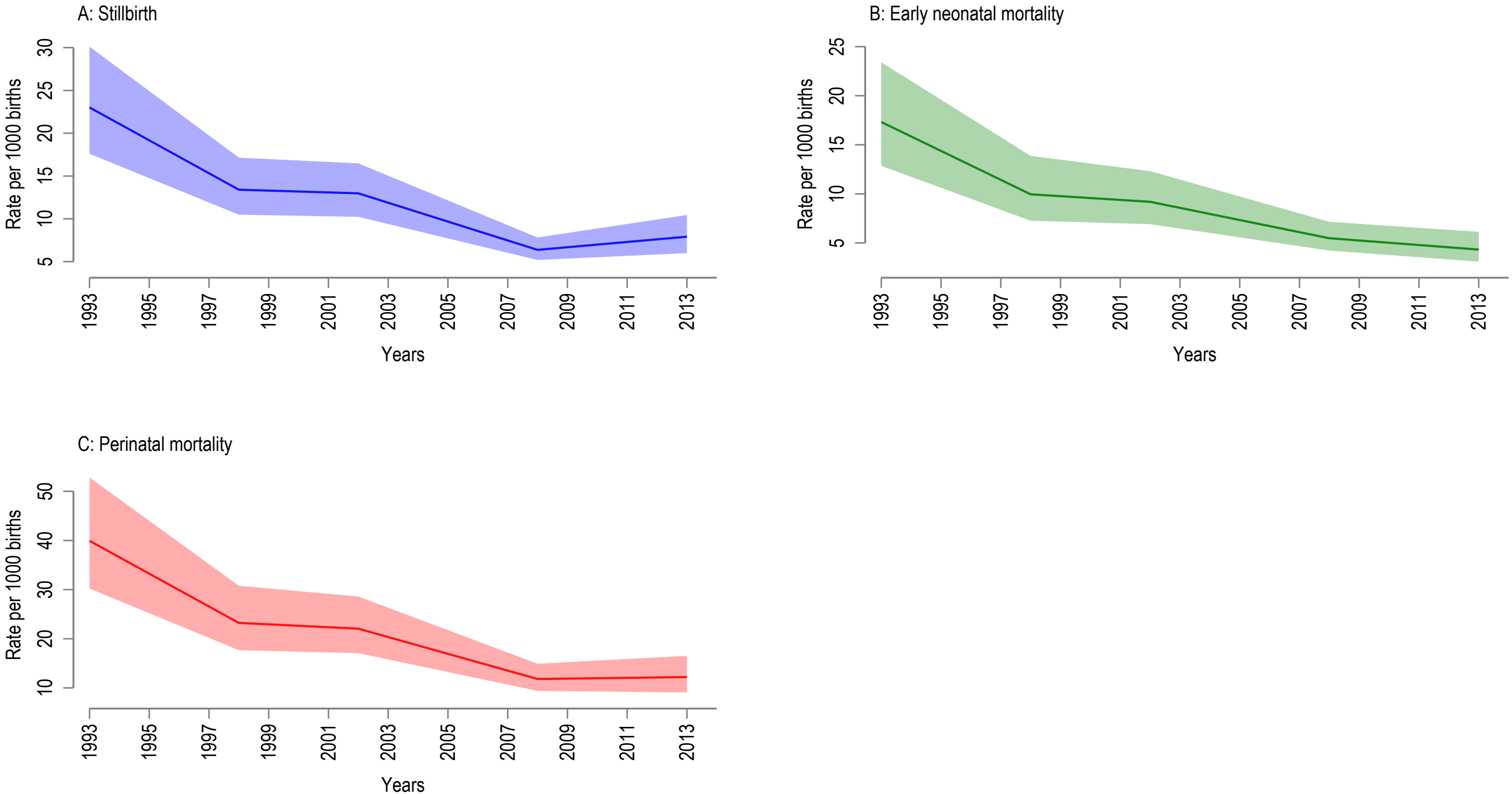

These are adjsted estimates

S3.17 Fig: Trends in Stillbirth, Early neonatal and Perinatal mortality -- Peru

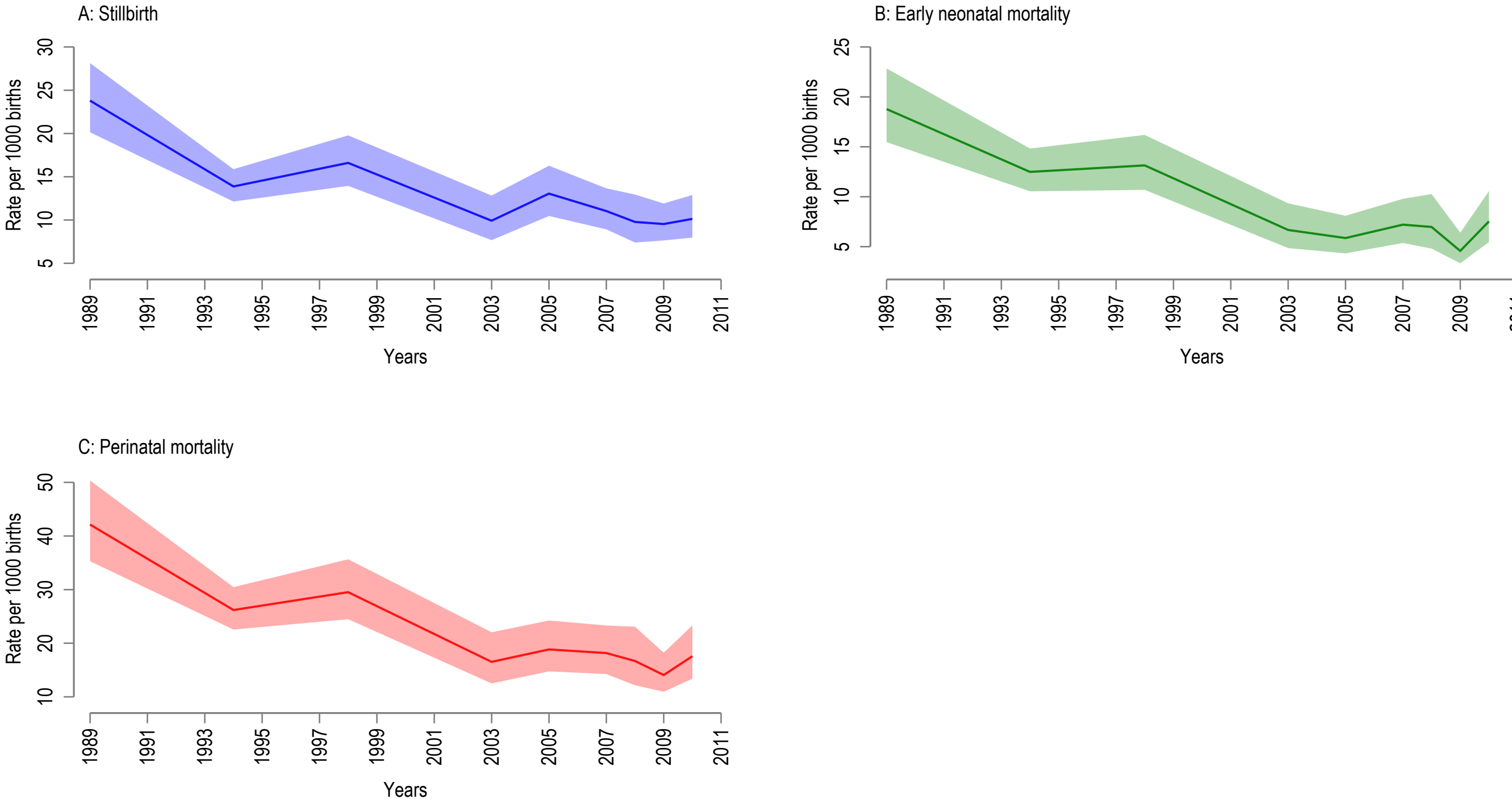

These are adjsted estimates
