# Supplementary material for: Trends of stillbirth, early neonatal and perinatal mortality rates and sociodemographic differentials of perinatal mortality rates in 17 low- and middle-income countries: A modeling study
Source: PLoS Med. 2026 Aug 24;23(8):e1004791. doi: 10.1371/journal.pmed.1004791 (PMC13502698; doi:10.1371/journal.pmed.1004791)
Supplement: Supplementary file 9 [file pmed.1004791.s009.pdf]

S4.1 Fig: Rate ratios of perinatal mortality, by survey

Comapring Urban vs Rural

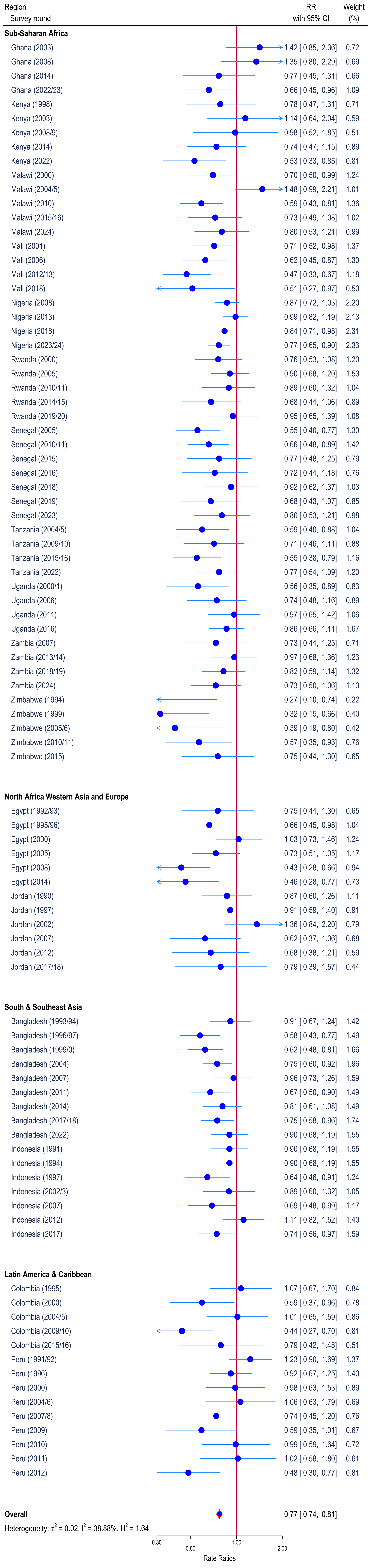

S4.2 Fig: Rate ratios of perinatal mortality, by survey

Comparing Primary vs No education

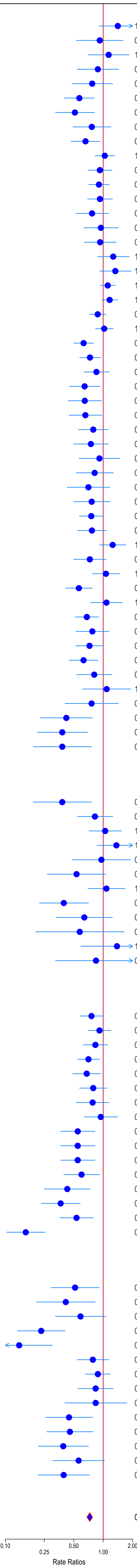

Region / Survey round	RR with 95% CI	Weight (%)
**Sub-Saharan Africa**		
Ghana (2003)	1.41 [0.91, 2.17]	1.06
Ghana (2008)	0.92 [0.54, 1.59]	0.88
Ghana (2014)	1.14 [0.70, 1.84]	0.99
Ghana (2022/23)	0.88 [0.54, 1.43]	0.98
Kenya (1998)	0.77 [0.48, 1.24]	0.99
Kenya (2003)	0.57 [0.40, 0.81]	1.21
Kenya (2008/9)	0.51 [0.33, 0.81]	1.03
Kenya (2014)	0.77 [0.49, 1.19]	1.04
Kenya (2022)	0.66 [0.47, 0.93]	1.23
Malawi (2000)	1.04 [0.83, 1.30]	1.43
Malawi (2004/5)	0.93 [0.70, 1.22]	1.34
Malawi (2010)	0.90 [0.72, 1.14]	1.42
Malawi (2015/16)	0.93 [0.69, 1.24]	1.32
Malawi (2024)	0.77 [0.53, 1.13]	1.16
Mali (2001)	0.95 [0.64, 1.41]	1.12
Mali (2006)	0.93 [0.64, 1.35]	1.17
Mali (2012/13)	1.27 [0.88, 1.83]	1.18
Mali (2018)	1.33 [0.92, 1.92]	1.18
Nigeria (2008)	1.11 [0.94, 1.32]	1.51
Nigeria (2013)	1.16 [0.97, 1.40]	1.49
Nigeria (2018)	0.88 [0.73, 1.06]	1.49
Nigeria (2023/24)	1.03 [0.84, 1.26]	1.47
Rwanda (2000)	0.63 [0.50, 0.79]	1.43
Rwanda (2005)	0.73 [0.58, 0.93]	1.40
Rwanda (2010/11)	0.85 [0.64, 1.14]	1.32
Rwanda (2014/15)	0.65 [0.45, 0.92]	1.20
Rwanda (2019/20)	0.65 [0.44, 0.96]	1.14
Senegal (2005)	0.66 [0.45, 0.96]	1.16
Senegal (2010/11)	0.79 [0.56, 1.12]	1.22
Senegal (2015)	0.75 [0.50, 1.12]	1.11
Senegal (2016)	0.92 [0.57, 1.48]	0.98
Senegal (2018)	0.82 [0.53, 1.26]	1.06
Senegal (2019)	0.71 [0.43, 1.16]	0.95
Senegal (2023)	0.76 [0.50, 1.17]	1.08
Tanzania (2004/5)	0.75 [0.57, 0.99]	1.35
Tanzania (2009/10)	0.77 [0.55, 1.08]	1.23
Tanzania (2015/16)	1.25 [0.92, 1.69]	1.29
Tanzania (2022)	0.73 [0.50, 1.06]	1.16
Uganda (2000/1)	1.07 [0.78, 1.46]	1.27
Uganda (2006)	0.56 [0.41, 0.77]	1.28
Uganda (2011)	1.08 [0.74, 1.57]	1.16
Uganda (2016)	0.68 [0.52, 0.90]	1.35
Zambia (2007)	0.78 [0.53, 1.14]	1.14
Zambia (2013/14)	0.72 [0.53, 1.00]	1.27
Zambia (2018/19)	0.63 [0.45, 0.88]	1.24
Zambia (2024)	0.81 [0.54, 1.22]	1.11
Zimbabwe (1994)	1.08 [0.62, 1.90]	0.86
Zimbabwe (1999)	0.76 [0.41, 1.42]	0.77
Zimbabwe (2005/6)	0.42 [0.23, 0.77]	0.79
Zimbabwe (2010/11)	0.38 [0.21, 0.69]	0.82
Zimbabwe (2015)	0.38 [0.19, 0.76]	0.70
**North Africa Western Asia and Europe**		
Egypt (1992/93)	0.38 [0.19, 0.76]	0.70
Egypt (1995/96)	0.82 [0.55, 1.24]	1.10
Egypt (2000)	1.05 [0.72, 1.53]	1.16
Egypt (2005)	1.37 [0.86, 2.16]	1.02
Egypt (2008)	0.96 [0.48, 1.90]	0.69
Egypt (2014)	0.53 [0.27, 1.06]	0.69
Jordan (1990)	1.08 [0.70, 1.66]	1.06
Jordan (1997)	0.40 [0.22, 0.70]	0.84
Jordan (2002)	0.64 [0.33, 1.23]	0.73
Jordan (2007)	0.58 [0.20, 1.63]	0.40
Jordan (2012)	1.38 [0.59, 3.23]	0.53
Jordan (2017/18)	0.84 [0.33, 2.18]	0.45
**South & Southeast Asia**		
Bangladesh (1993/94)	0.76 [0.58, 0.99]	1.36
Bangladesh (1996/97)	0.92 [0.71, 1.19]	1.37
Bangladesh (1999/0)	0.83 [0.63, 1.10]	1.33
Bangladesh (2004)	0.71 [0.55, 0.91]	1.38
Bangladesh (2007)	0.68 [0.49, 0.94]	1.25
Bangladesh (2011)	0.79 [0.57, 1.09]	1.26
Bangladesh (2014)	0.78 [0.53, 1.14]	1.16
Bangladesh (2017/18)	0.94 [0.64, 1.39]	1.14
Bangladesh (2022)	0.55 [0.37, 0.82]	1.12
Indonesia (1991)	0.55 [0.37, 0.82]	1.12
Indonesia (1994)	0.55 [0.37, 0.82]	1.12
Indonesia (1997)	0.60 [0.40, 0.91]	1.10
Indonesia (2002/3)	0.43 [0.25, 0.74]	0.89
Indonesia (2007)	0.37 [0.23, 0.58]	1.03
Indonesia (2012)	0.53 [0.36, 0.79]	1.14
Indonesia (2017)	0.16 [0.10, 0.25]	1.03
**Latin America & Caribbean**		
Colombia (1995)	0.52 [0.29, 0.91]	0.86
Colombia (2000)	0.41 [0.21, 0.82]	0.69
Colombia (2004/5)	0.59 [0.32, 1.06]	0.82
Colombia (2009/10)	0.23 [0.13, 0.41]	0.86
Colombia (2015/16)	0.14 [0.06, 0.30]	0.60
Peru (1991/92)	0.78 [0.54, 1.13]	1.18
Peru (1996)	0.88 [0.66, 1.18]	1.32
Peru (2000)	0.84 [0.56, 1.26]	1.10
Peru (2004/6)	0.84 [0.41, 1.73]	0.65
Peru (2007/8)	0.45 [0.26, 0.78]	0.87
Peru (2009)	0.46 [0.27, 0.78]	0.89
Peru (2010)	0.39 [0.22, 0.70]	0.82
Peru (2011)	0.56 [0.31, 1.03]	0.79
Peru (2012)	0.39 [0.22, 0.72]	0.80
**Overall**	0.73 [0.68, 0.79]	

Heterogeneity: $\tau^2 = 0.09$, $I^2 = 72.61\%$, $H^2 = 3.65$

S4.3 Fig: Rate ratios of perinatal mortality, by survey

Comparing Secondary education vs No education

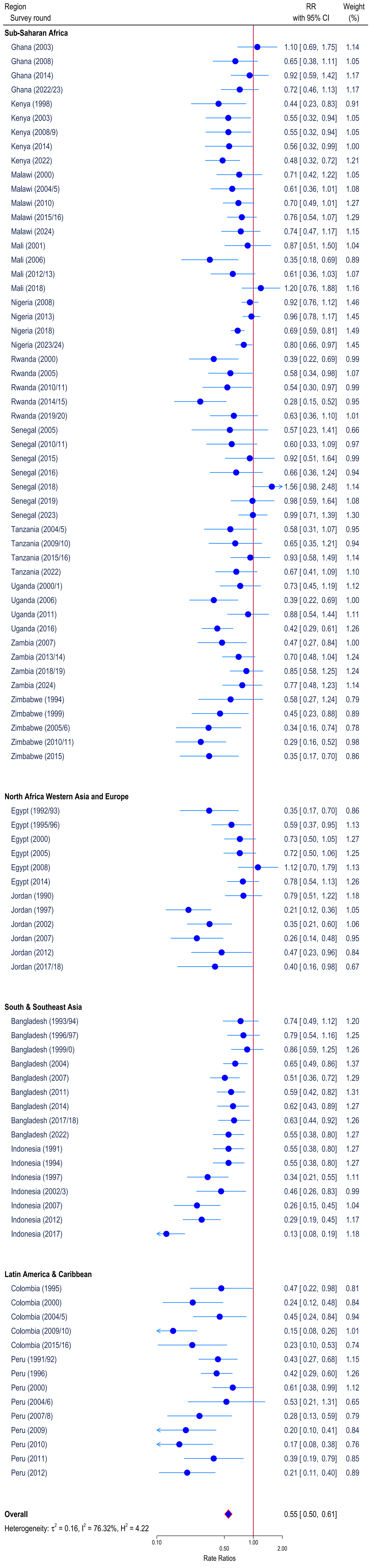

S4.4 Fig: Rate ratios of perinatal mortality, by survey

Comparing Middle HH wealth vs Poor HH

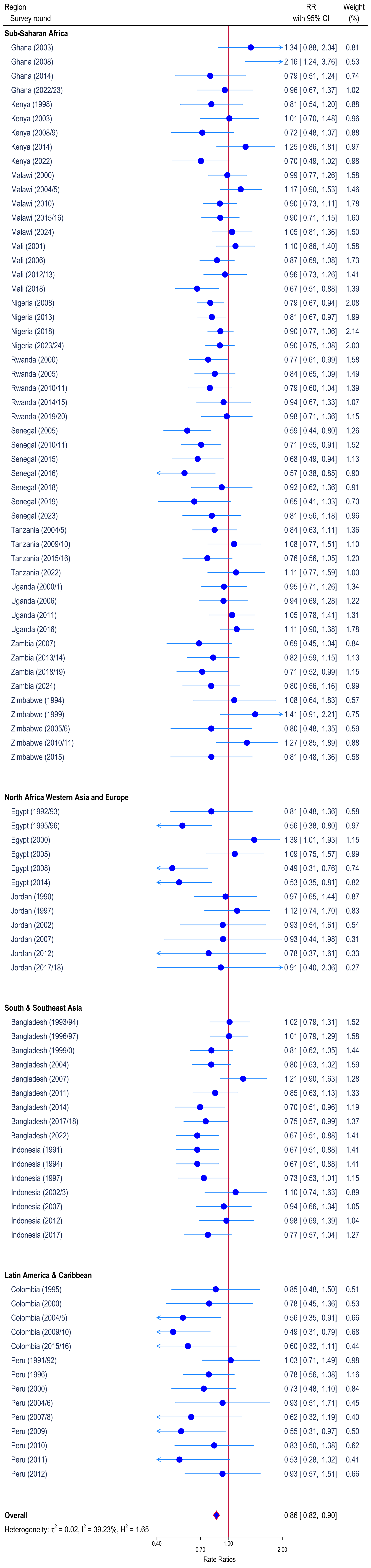

S4.5 Fig: Rate ratios of perinatal mortality, by survey

Comparing Rich HH wealth vs Poor HH

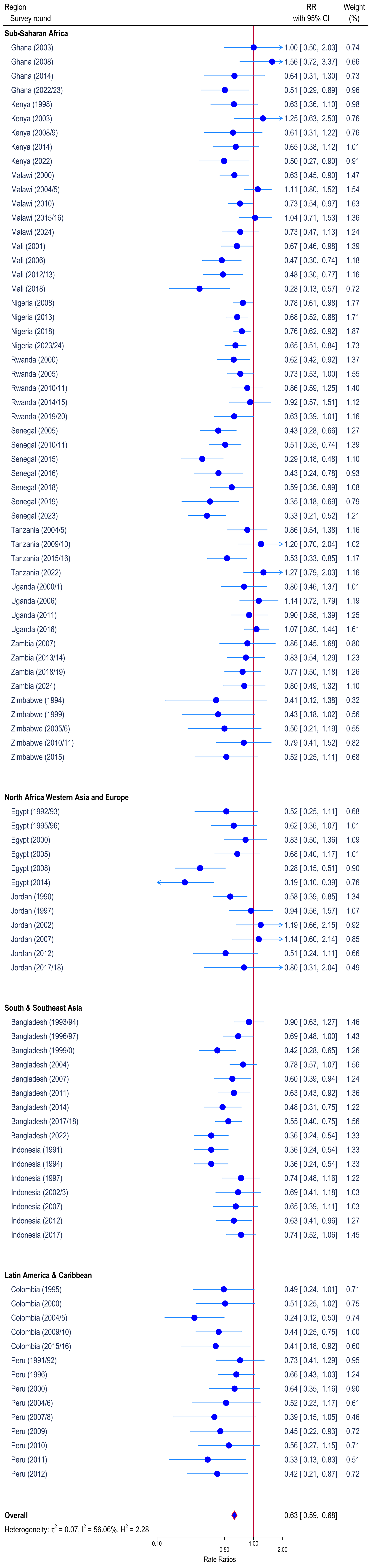

S4.6 Fig: Rate ratios of perinatal mortality, by survey

Comparing Age 25-29 vs Age below 25

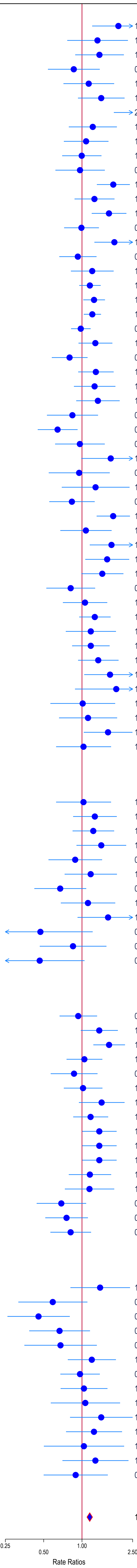

Region / Survey round	RR with 95% CI	Weight (%)
**Sub-Saharan Africa**		
Ghana (2003)	1.93 [1.21, 3.09]	0.91
Ghana (2008)	1.32 [0.77, 2.28]	0.75
Ghana (2014)	1.37 [0.89, 2.12]	1.00
Ghana (2022/23)	0.86 [0.54, 1.37]	0.93
Kenya (1998)	1.13 [0.72, 1.78]	0.95
Kenya (2003)	1.42 [0.94, 2.14]	1.06
Kenya (2008/9)	2.84 [1.79, 4.53]	0.92
Kenya (2014)	1.22 [0.79, 1.86]	1.02
Kenya (2022)	1.08 [0.72, 1.60]	1.11
Malawi (2000)	1.00 [0.70, 1.41]	1.27
Malawi (2004/5)	0.97 [0.62, 1.50]	0.98
Malawi (2010)	1.76 [1.32, 2.36]	1.49
Malawi (2015/16)	1.25 [0.88, 1.79]	1.25
Malawi (2024)	1.63 [1.20, 2.22]	1.43
Mali (2001)	0.99 [0.73, 1.35]	1.42
Mali (2006)	1.80 [1.26, 2.57]	1.24
Mali (2012/13)	0.93 [0.67, 1.29]	1.34
Mali (2018)	1.20 [0.82, 1.76]	1.16
Nigeria (2008)	1.15 [0.96, 1.39]	1.96
Nigeria (2013)	1.24 [1.03, 1.50]	1.95
Nigeria (2018)	1.21 [1.04, 1.40]	2.13
Nigeria (2023/24)	0.98 [0.83, 1.16]	2.04
Rwanda (2000)	1.27 [0.94, 1.72]	1.46
Rwanda (2005)	0.80 [0.58, 1.10]	1.39
Rwanda (2010/11)	1.28 [0.94, 1.76]	1.40
Rwanda (2014/15)	1.26 [0.87, 1.82]	1.20
Rwanda (2019/20)	1.33 [0.91, 1.96]	1.14
Senegal (2005)	0.84 [0.53, 1.33]	0.94
Senegal (2010/11)	0.64 [0.45, 0.92]	1.25
Senegal (2015)	0.96 [0.62, 1.50]	0.98
Senegal (2016)	1.68 [0.99, 2.86]	0.78
Senegal (2018)	0.95 [0.55, 1.64]	0.74
Senegal (2019)	1.28 [0.70, 2.34]	0.64
Senegal (2023)	0.83 [0.56, 1.25]	1.09
Tanzania (2004/5)	1.76 [1.31, 2.36]	1.48
Tanzania (2009/10)	1.07 [0.68, 1.70]	0.94
Tanzania (2015/16)	1.71 [1.16, 2.52]	1.14
Tanzania (2022)	1.58 [1.07, 2.33]	1.13
Uganda (2000/1)	1.44 [0.99, 2.10]	1.18
Uganda (2006)	0.81 [0.53, 1.26]	1.00
Uganda (2011)	1.06 [0.71, 1.57]	1.12
Uganda (2016)	1.26 [0.96, 1.67]	1.55
Zambia (2007)	1.17 [0.75, 1.83]	0.96
Zambia (2013/14)	1.17 [0.84, 1.64]	1.33
Zambia (2018/19)	1.34 [0.94, 1.92]	1.23
Zambia (2024)	1.66 [1.05, 2.64]	0.93
Zimbabwe (1994)	1.86 [0.89, 3.90]	0.47
Zimbabwe (1999)	1.01 [0.57, 1.81]	0.68
Zimbabwe (2005/6)	1.12 [0.66, 1.87]	0.80
Zimbabwe (2010/11)	1.60 [1.04, 2.47]	1.00
Zimbabwe (2015)	1.03 [0.63, 1.68]	0.86
**North Africa Western Asia and Europe**		
Egypt (1992/93)	1.03 [0.63, 1.68]	0.86
Egypt (1995/96)	1.26 [0.86, 1.86]	1.14
Egypt (2000)	1.23 [0.85, 1.78]	1.20
Egypt (2005)	1.42 [0.91, 2.21]	0.97
Egypt (2008)	0.88 [0.55, 1.43]	0.88
Egypt (2014)	1.17 [0.73, 1.87]	0.91
Jordan (1990)	0.67 [0.42, 1.07]	0.92
Jordan (1997)	1.11 [0.69, 1.81]	0.87
Jordan (2002)	1.60 [0.93, 2.77]	0.74
Jordan (2007)	0.47 [0.18, 1.20]	0.31
Jordan (2012)	0.85 [0.47, 1.54]	0.65
Jordan (2017/18)	0.47 [0.21, 1.04]	0.41
**South & Southeast Asia**		
Bangladesh (1993/94)	0.93 [0.67, 1.30]	1.33
Bangladesh (1996/97)	1.37 [0.98, 1.90]	1.34
Bangladesh (1999/0)	1.63 [1.23, 2.16]	1.54
Bangladesh (2004)	1.04 [0.76, 1.43]	1.39
Bangladesh (2007)	0.87 [0.57, 1.31]	1.05
Bangladesh (2011)	1.02 [0.72, 1.44]	1.29
Bangladesh (2014)	1.43 [0.95, 2.14]	1.08
Bangladesh (2017/18)	1.17 [0.86, 1.59]	1.42
Bangladesh (2022)	1.37 [1.01, 1.86]	1.44
Indonesia (1991)	1.37 [1.01, 1.86]	1.44
Indonesia (1994)	1.37 [1.01, 1.86]	1.44
Indonesia (1997)	1.15 [0.79, 1.68]	1.17
Indonesia (2002/3)	1.15 [0.74, 1.78]	0.98
Indonesia (2007)	0.69 [0.44, 1.07]	0.98
Indonesia (2012)	0.76 [0.52, 1.10]	1.17
Indonesia (2017)	0.81 [0.57, 1.17]	1.23
**Latin America & Caribbean**		
Colombia (1995)	1.39 [0.82, 2.36]	0.77
Colombia (2000)	0.59 [0.32, 1.09]	0.62
Colombia (2004/5)	0.46 [0.26, 0.80]	0.72
Colombia (2009/10)	0.67 [0.39, 1.15]	0.75
Colombia (2015/16)	0.68 [0.35, 1.30]	0.57
Peru (1991/92)	1.19 [0.78, 1.83]	1.02
Peru (1996)	0.97 [0.68, 1.37]	1.27
Peru (2000)	1.04 [0.68, 1.57]	1.05
Peru (2004/6)	1.06 [0.57, 1.98]	0.62
Peru (2007/8)	1.42 [0.81, 2.48]	0.72
Peru (2009)	1.24 [0.75, 2.05]	0.84
Peru (2010)	1.04 [0.50, 2.13]	0.49
Peru (2011)	1.27 [0.71, 2.30]	0.66
Peru (2012)	0.89 [0.50, 1.58]	0.69
**Overall**	1.15 [1.09, 1.22]	

Heterogeneity: $\tau^2 = 0.03$, $I^2 = 47.54\%$, $H^2 = 1.91$

S4.7 Fig: Rate ratios of perinatal mortality, by survey

Comparing Age 30+ vs Age below 25

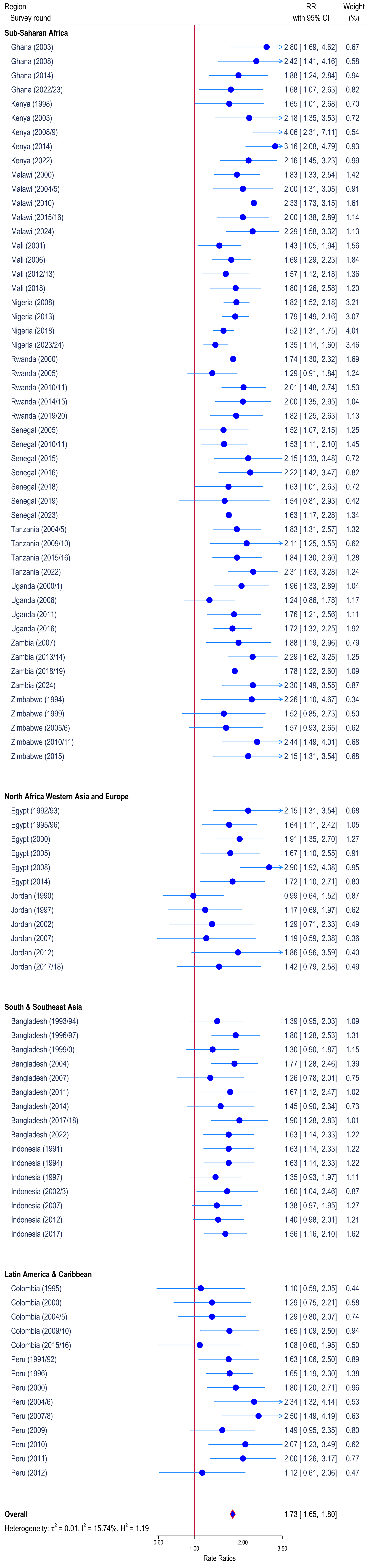

S4.8 Fig: Rate ratios of perinatal mortality, by survey

Comparing Living children 2+ vs Living children below 2

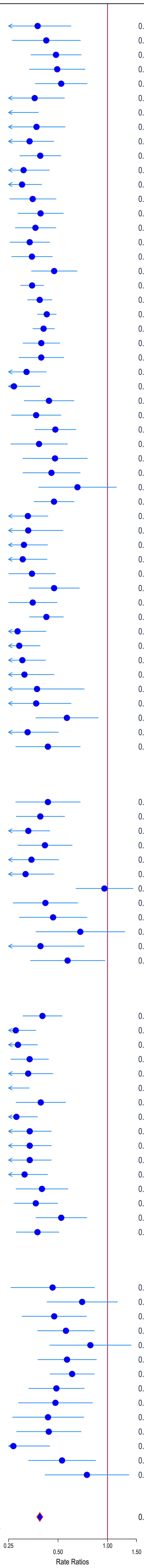

Region / Survey round	RR with 95% CI	Weight (%)
**Sub-Saharan Africa**		
Ghana (2003)	0.38 [0.24, 0.60]	0.74
Ghana (2008)	0.42 [0.26, 0.68]	0.72
Ghana (2014)	0.49 [0.34, 0.69]	1.09
Ghana (2022/23)	0.49 [0.34, 0.73]	0.95
Kenya (1998)	0.52 [0.36, 0.75]	1.04
Kenya (2003)	0.36 [0.24, 0.54]	0.87
Kenya (2008/9)	0.24 [0.15, 0.38]	0.72
Kenya (2014)	0.37 [0.25, 0.55]	0.92
Kenya (2022)	0.34 [0.24, 0.47]	1.14
Malawi (2000)	0.39 [0.29, 0.52]	1.36
Malawi (2004/5)	0.31 [0.22, 0.44]	1.05
Malawi (2010)	0.30 [0.23, 0.40]	1.43
Malawi (2015/16)	0.35 [0.25, 0.48]	1.19
Malawi (2024)	0.39 [0.29, 0.54]	1.22
Mali (2001)	0.36 [0.27, 0.48]	1.37
Mali (2006)	0.34 [0.26, 0.44]	1.41
Mali (2012/13)	0.35 [0.26, 0.46]	1.37
Mali (2018)	0.47 [0.36, 0.62]	1.21
Nigeria (2008)	0.35 [0.30, 0.41]	2.08
Nigeria (2013)	0.39 [0.33, 0.46]	2.02
Nigeria (2018)	0.43 [0.38, 0.49]	2.27
Nigeria (2023/24)	0.41 [0.35, 0.47]	2.15
Rwanda (2000)	0.40 [0.31, 0.51]	1.51
Rwanda (2005)	0.40 [0.29, 0.54]	1.24
Rwanda (2010/11)	0.32 [0.24, 0.42]	1.42
Rwanda (2014/15)	0.27 [0.19, 0.39]	1.04
Rwanda (2019/20)	0.44 [0.31, 0.62]	1.10
Senegal (2005)	0.37 [0.26, 0.52]	1.11
Senegal (2010/11)	0.48 [0.36, 0.64]	1.37
Senegal (2015)	0.38 [0.26, 0.57]	0.93
Senegal (2016)	0.48 [0.31, 0.75]	0.78
Senegal (2018)	0.46 [0.31, 0.68]	0.92
Senegal (2019)	0.66 [0.38, 1.13]	0.58
Senegal (2023)	0.47 [0.36, 0.62]	1.40
Tanzania (2004/5)	0.33 [0.25, 0.43]	1.40
Tanzania (2009/10)	0.33 [0.20, 0.53]	0.70
Tanzania (2015/16)	0.31 [0.22, 0.43]	1.17
Tanzania (2022)	0.30 [0.22, 0.43]	1.13
Uganda (2000/1)	0.35 [0.25, 0.48]	1.18
Uganda (2006)	0.47 [0.33, 0.67]	1.08
Uganda (2011)	0.35 [0.25, 0.49]	1.13
Uganda (2016)	0.42 [0.34, 0.54]	1.63
Zambia (2007)	0.28 [0.19, 0.42]	0.93
Zambia (2013/14)	0.29 [0.22, 0.39]	1.34
Zambia (2018/19)	0.30 [0.22, 0.42]	1.19
Zambia (2024)	0.31 [0.21, 0.47]	0.88
Zimbabwe (1994)	0.37 [0.19, 0.72]	0.43
Zimbabwe (1999)	0.37 [0.23, 0.60]	0.70
Zimbabwe (2005/6)	0.57 [0.37, 0.87]	0.81
Zimbabwe (2010/11)	0.33 [0.21, 0.50]	0.83
Zimbabwe (2015)	0.43 [0.28, 0.68]	0.78
**North Africa Western Asia and Europe**		
Egypt (1992/93)	0.43 [0.28, 0.68]	0.78
Egypt (1995/96)	0.39 [0.28, 0.55]	1.14
Egypt (2000)	0.33 [0.24, 0.44]	1.30
Egypt (2005)	0.42 [0.29, 0.61]	0.99
Egypt (2008)	0.34 [0.24, 0.50]	0.98
Egypt (2014)	0.32 [0.21, 0.47]	0.94
Jordan (1990)	0.96 [0.64, 1.42]	0.92
Jordan (1997)	0.42 [0.27, 0.66]	0.78
Jordan (2002)	0.47 [0.29, 0.75]	0.72
Jordan (2007)	0.68 [0.37, 1.27]	0.47
Jordan (2012)	0.39 [0.21, 0.72]	0.49
Jordan (2017/18)	0.57 [0.34, 0.96]	0.63
**South & Southeast Asia**		
Bangladesh (1993/94)	0.40 [0.31, 0.53]	1.42
Bangladesh (1996/97)	0.28 [0.21, 0.36]	1.41
Bangladesh (1999/0)	0.29 [0.22, 0.37]	1.43
Bangladesh (2004)	0.34 [0.26, 0.44]	1.48
Bangladesh (2007)	0.33 [0.23, 0.46]	1.11
Bangladesh (2011)	0.25 [0.18, 0.33]	1.28
Bangladesh (2014)	0.39 [0.28, 0.55]	1.10
Bangladesh (2017/18)	0.28 [0.21, 0.37]	1.33
Bangladesh (2022)	0.34 [0.25, 0.45]	1.29
Indonesia (1991)	0.34 [0.25, 0.45]	1.29
Indonesia (1994)	0.34 [0.25, 0.45]	1.29
Indonesia (1997)	0.31 [0.23, 0.43]	1.21
Indonesia (2002/3)	0.40 [0.28, 0.57]	1.04
Indonesia (2007)	0.37 [0.27, 0.49]	1.28
Indonesia (2012)	0.52 [0.37, 0.74]	1.07
Indonesia (2017)	0.37 [0.28, 0.50]	1.31
**Latin America & Caribbean**		
Colombia (1995)	0.46 [0.26, 0.83]	0.52
Colombia (2000)	0.70 [0.43, 1.14]	0.68
Colombia (2004/5)	0.47 [0.30, 0.74]	0.78
Colombia (2009/10)	0.56 [0.38, 0.83]	0.93
Colombia (2015/16)	0.79 [0.45, 1.39]	0.54
Peru (1991/92)	0.57 [0.38, 0.85]	0.89
Peru (1996)	0.61 [0.45, 0.83]	1.25
Peru (2000)	0.49 [0.33, 0.72]	0.96
Peru (2004/6)	0.48 [0.29, 0.81]	0.63
Peru (2007/8)	0.43 [0.26, 0.71]	0.67
Peru (2009)	0.44 [0.28, 0.69]	0.78
Peru (2010)	0.27 [0.16, 0.44]	0.65
Peru (2011)	0.53 [0.33, 0.84]	0.73
Peru (2012)	0.75 [0.42, 1.34]	0.52
**Overall**	0.39 [0.37, 0.41]	

Heterogeneity: $\tau^2 = 0.02$, $I^2 = 43.50\%$, $H^2 = 1.77$
