# Supplementary material for: Trends of stillbirth, early neonatal and perinatal mortality rates and sociodemographic differentials of perinatal mortality rates in 17 low- and middle-income countries: A modeling study
Source: PLoS Med. 2026 Aug 24;23(8):e1004791. doi: 10.1371/journal.pmed.1004791 (PMC13502698; doi:10.1371/journal.pmed.1004791)

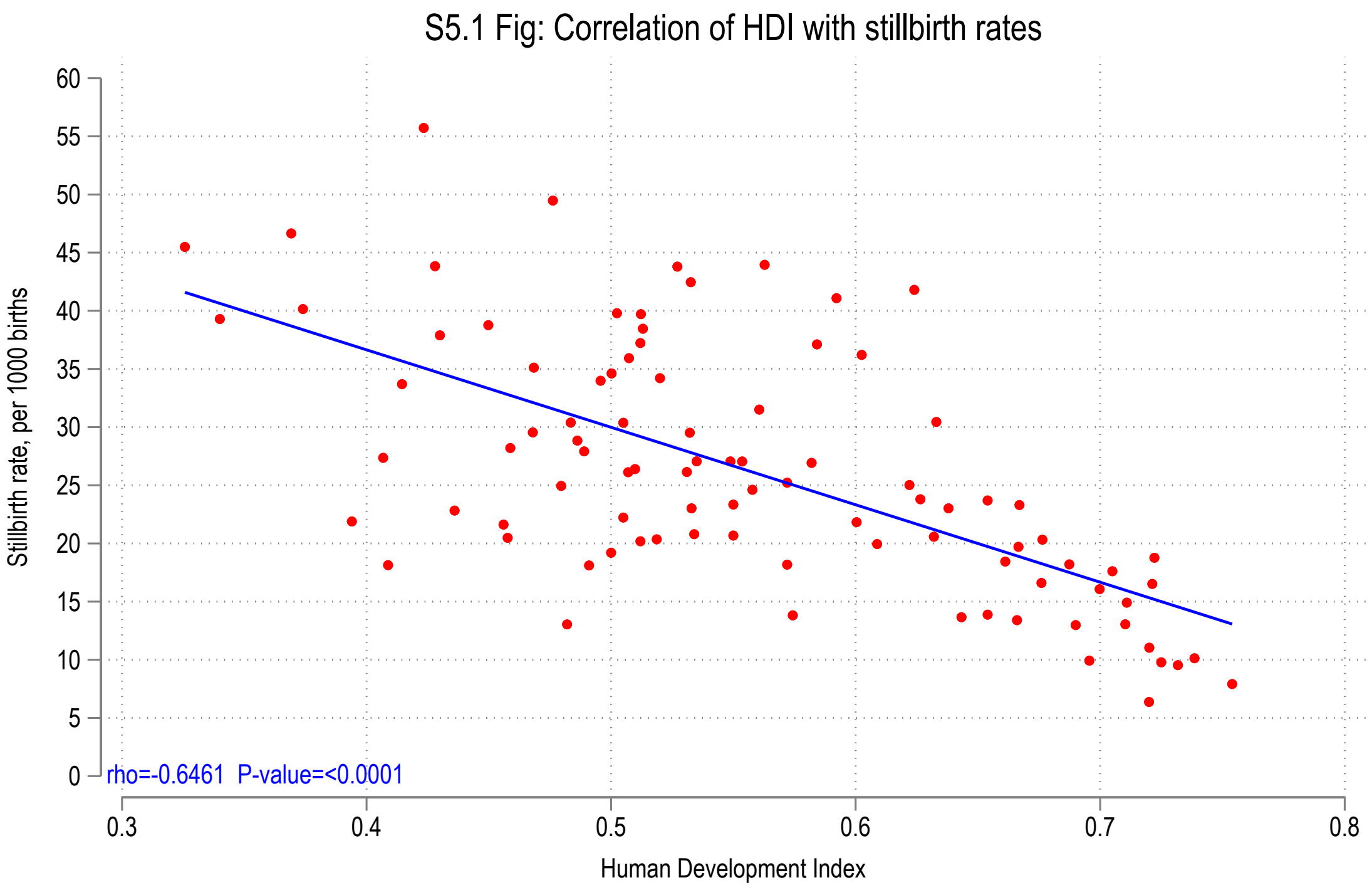
S5.1 Fig: Correlation of HDI with stillbirth rates
Stillbirth rate, per 1000 births
60
55
50
45
40
35
30
25
20
15
10
5
0
rho=-0.6461 P-value=<0.0001
0.3
0.4
0.5
0.6
0.7
0.8
Human Development Index

S5.2 Fig: Correlation of HDI with early neonatal mortalty rates

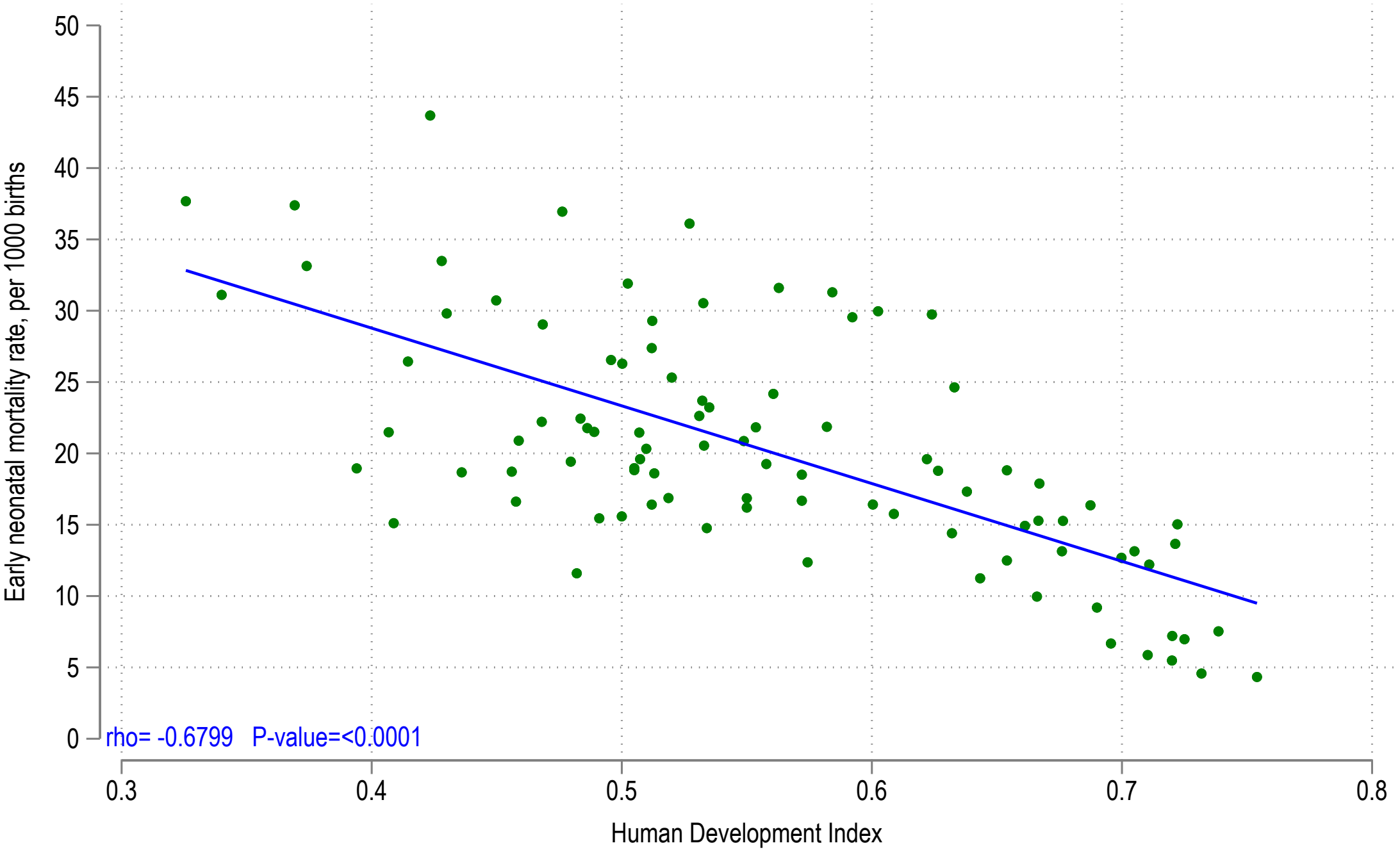

S5.3 Fig: Correlation of HDI with perinatal mortalty rates

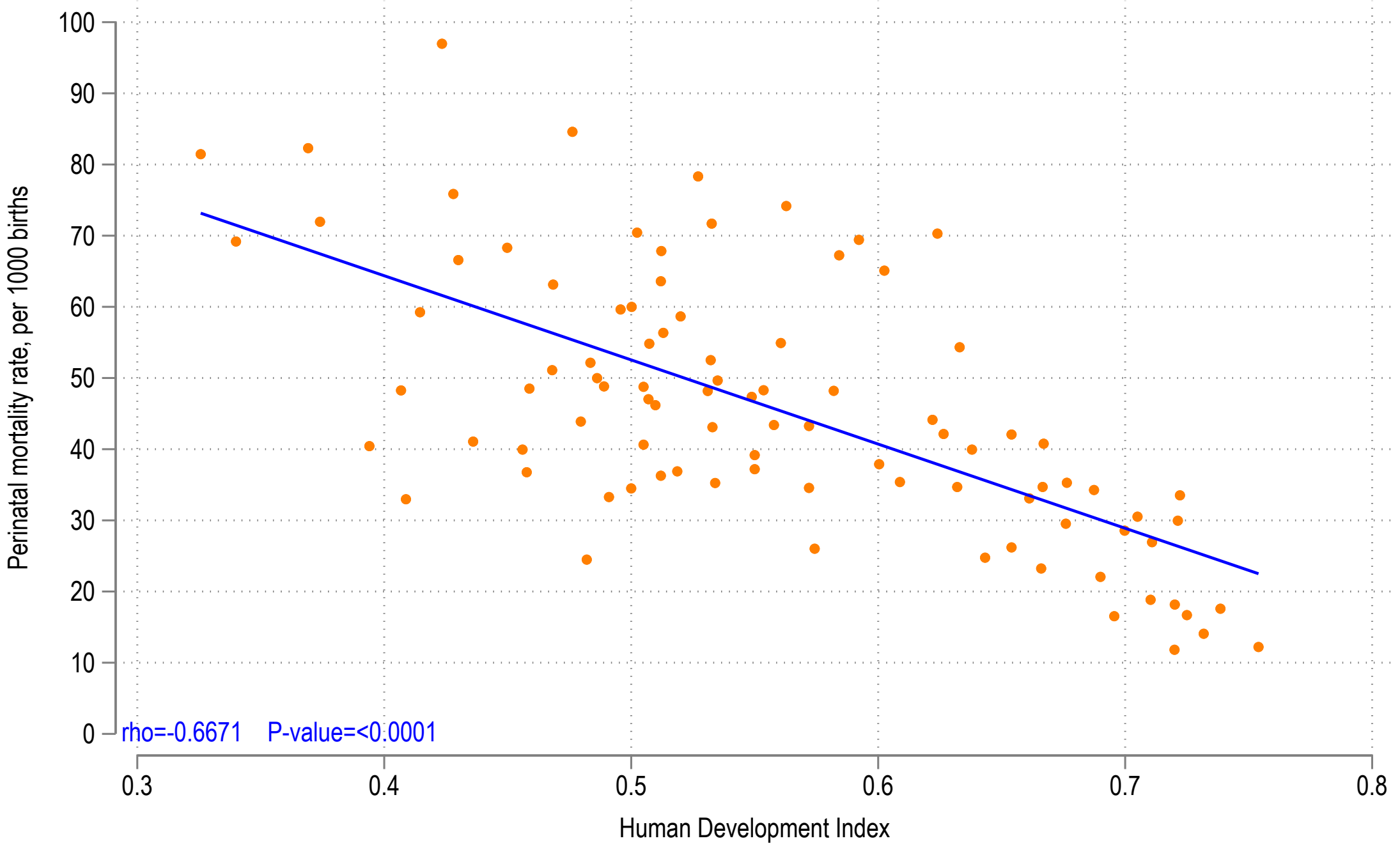

Supplement: Supplementary file 10 [file pmed.1004791.s010.pdf]
