# Supplementary material for: Trends of stillbirth, early neonatal and perinatal mortality rates and sociodemographic differentials of perinatal mortality rates in 17 low- and middle-income countries: A modeling study
Source: PLoS Med. 2026 Aug 24;23(8):e1004791. doi: 10.1371/journal.pmed.1004791 (PMC13502698; doi:10.1371/journal.pmed.1004791)
Supplement: Supplementary file 12 [file pmed.1004791.s013.docx]

Dear Editor,

Many thanks for the rich feedbacks and for giving the opportunity to improve our scientific paper.

As recommended, we merged methods and findings in the abstract, we added the author summary, and we formatted references.

We also expanded our analysis that now includes 16 countries and we reflected the early neonatal mortality (title of the paper amended based on these two important aspects).

Below are our responses to reviewers.

Reviewer #1: This is an interesting paper that draws on a recently described method by the same team to examine trends of stillbirth and neonatal death over time. They have used a Gompertz-Makeham model, which aims to adjust for misreporting/transfer bias. However, as written, the manuscript is difficult to follow, the analytical approach is not sufficiently transparent, and several key methodological and interpretative issues require clarification before the findings can be reliably interpreted. In particular, the description of how trends over time were derived, and how misclassification adjustments influence those trends, requires clearer explanation and sensitivity analyses.

*Many thanks and we addressed all these aspects throughout the manuscript.*

Abstract: Further detail of the Gompertz-Makeham model is needed to give context to the paper The reproductive calendar should be briefly defined, including acknowledgement of known under-reporting of stillbirth and misclassification to neonatal deaths. Abstract results should include point estimates. Define income rather than rich/poor. This sentence is unclear 'however for prenatal mortality all covariates examined were significant associated with perinatal mortality.' The objective of the study and difference to the previous study needs more clarity.

*Many thanks. We re-wrote the abstract, which now is as follows:*

***Abstract***

***Background***

*Stillbirths and early neonatal deaths account for a substantial proportion of global child mortality. Reliable measurement of these outcomes remains challenging in settings where civil registration systems are incomplete and household surveys are the primary data source. However, survey-based estimates are often affected by underreporting and misclassification between stillbirths and early neonatal deaths. We aimed to apply a statistical adjustment approach to improve estimates of stillbirth, early neonatal, and perinatal mortality trends and to assess socioeconomic inequalities in countries with repeated Demographic and Health Surveys (DHS).*

***Methods and findings***

*We analyzed 89 DHS surveys with reproductive calendar data from 16 LMICs conducted between 1990 and 2023, covering 979,579 pregnancies reported by 708,435 women. Pregnancies of at least 7 months’ gestation were included to align with the standard definition of stillbirth. Age-specific risks of stillbirth and early neonatal death were estimated using life-table methods and a modified Gompertz–Makeham (mGM) model that adjusts for common survey data quality issues, including underreporting, misclassification between stillbirths and deaths on days 0–1, and age-at-death heaping. Survey-specific estimates were used to evaluate trends over time and to examine associations with maternal and household characteristics through random-effects meta-regression.*

*Across countries, stillbirth, early neonatal, and perinatal mortality rates generally declined over time, although trends varied substantially between settings. The pooled annual decline was −0.62 deaths per 1,000 births for stillbirths (95% CI −0.81 to −0.43), −0.53 for early neonatal mortality (95% CI −0.69 to −0.38), and −1.13 for perinatal mortality (95% CI −1.46 to −0.80). Lower perinatal mortality was associated with urban residence, higher maternal education, greater household wealth, and higher national Human Development Index levels, whereas maternal age ≥30 years was associated with higher risk. Limitations include potential residual underreporting of pregnancy losses and lack of information on causes of death.*

***Conclusions***

*Adjusting for data quality issues substantially improves the use of household survey data for estimating stillbirth and early neonatal mortality. Although most countries experienced declines in perinatal mortality, progress remains uneven and significant socioeconomic disparities persist. Strengthening maternal and newborn health services and improving data systems will be essential to accelerate progress toward global targets for reducing perinatal mortality.*

Introduction:

-	Definition of neonatal death is incorrect - should be up to 28 days and should state WHO definition of stillbirth.

*We added the WHO reference for the definition of stillbirth in line 92. In addition we clarified that we are examining early neonatal mortality in line 93.*

-	Additional background on DHS surveys is warranted, including their central role in producing global perinatal mortality estimates and their known limitations (recall error, misclassification).

*We added a paragraph on DHS from line 110 to 115, which reads as follows: The Demographic and Health Survey (DHS) program plays a central role in generating nationally representative data on maternal and child health in low- and middle-income countries and is a key source for global perinatal and stillbirth mortality estimates, including those produced by the United Nations Inter-Agency Group for Child Mortality Estimation (UN IGME) [3]. DHS birth histories and reproductive calendars enable standardized cross-country comparisons over time.*

-	More detail describing the prior study introducing the mGM approach would help establish rationale.

*This was reflected in lines 123 to 128: Specifically, a flexible statistical Gompertz-Makeham (mGM) model was developed to adjust for data quality collected by the DHS in measuring the stillbirth and the early neonatal mortality collected within the same survey by adjusting for possible omission, transference, and under-reporting, as well as smoothing; the model was established checking such indicators against figures from more reliable sources and proposing cut-offs for adjustment.*

Methods:

Overall, the objectives, and use of the mGM model are not described with sufficient clarity for reproducibility.

-	The methods do not state the objective of the study and overall are difficult to follow.

*We introduced the objective at the end of the introduction as follows: We aimed to apply the proposed adjustment methodology to estimate trends in stillbirth, early neonatal, and perinatal mortality rates, and to assess inequalities in perinatal mortality in countries with at least four DHS surveys with reproductive calendar, thereby improving the use of survey-based data in settings where death registration systems are inadequate.*

-	The approach used to estimate trends over time must be clarified. It is not clear whether calendar time was modelled as a continuous variable, a categorical survey indicator. The model specification should include the time term.

*The calendar length is around 5 years and the mid-calendar year was used as a continuous variable, the model included a term for the year for each survey (survey calendar mid year) see the supp table 2. We provided additional elements in the data source paragraph of the methods section from line 157 to 168: The reproductive calendar takes the form of a grid, where interviewers are trained to enter on the monthly grid all live births (and current pregnancy if any), ascertained earlier in the interview using the FBH module. Any pregnancy loss detected at this stage of detailed questioning is entered into the calendar, but no attempt is made to distinguish induced from spontaneous abortions. The exact length of the period covered by the calendar varies depending on the defined recall period and duration of the survey data collection. In most surveys, the period covered by the calendar includes the months up to the month of interview in the year of interview, plus the five calendar years preceding the year of interview. The reproductive calendar records the current pregnancy and the pregnancy loss in months and the FBH module records the child death in days, months or years, and current age in months, therefore the exact gestational age at pregnancy loss or age at death or current age are not reported.*

-	Because the mGM model includes misclassification constraints, uncertainty from these adjustments should be assessed in sensitivity analyses

*We understand the request of the reviewer. However, we do not see the need for sensitivity analysis, as we have used thresholds for the two quality indicators incorporated in the model*

-	It's not stated what years of births the surveys represent in methods, this should be included for each and does impact the applicability of the findings, especially for data >10 years old.

*As explained din the method section, the calendar only covers 5-6 years and we have retained pregnancies conceived during the 7-66 months before the survey*

-	Using gestational age coded in months should be acknowledged as a limitation due to possible misclassification of stillbirth eligibility.

*We addressed an important series of limitations from lines 91 to 402: Indeed, our study had several limitations. Fetal sex was not recorded, and ethnicity data were inconsistent. Another limitation is that we do not have data on whether the stillbirth was antepartum or intrapartum, or cause of neonatal death. Severe under-reporting of stillbirths in surveys remained a key data quality issue, and although model-based adjustments improved mortality estimates, some surveys still showed lower-than-expected stillbirth rates with wide uncertainty. This seems for instance the case for Tanzania and Kenya, where probably trends are not completely reliable due to earlier survey value being too low. Furthermore, as per the original study by Ali et al., we assumed quality metrics were constant to simplify analyses, which is line also with the observation of Perin et al. on assuming a constant cause of mortality distribution across the neonatal period [30]; in addition, we applied the DHS 7-month gestational age cutoff (based on the WHO 28-week definition) whereas a more precise gestational age reporting could strengthen future models.*

-	The assumption that births occurring in the month of interview are >1 week old may underestimate deaths on day 0-1; please report whether a sensitivity analysis was conducted assuming alternative ages.

This >1 week older assumption is based on the fact that If a respondent has just given birth, interviewers are encouraged to be flexible. If assessments or interviews are planned, they can often be scheduled or delayed until the mother has recovered. Accommodation for Post-Birth Recovery: If the respondent is recovering from childbirth, especially a C-section, they can request that home visits be delayed or scheduled after they have recovered. Assessment of the Newborn: The child is considered the primary client and must be directly seen and assessed (including checking on the baby's health) during the initial visit, even if this is delayed to accommodate recovery. In the surveys used in this analysis the number of livebirth with zero month ranged from 0.5% to 1.4%, and those maybe younger than one week are 0.1% to 0.3%, which is a negligible number, which will not have impact on death on day 0-1. Therefore, not sensitivity analysis I warranted.

-	Please describe how multiple births were handled in both numerators and denominators.

*Pregnancies that ended in multiple live births were retained, and the survival status of each live birth was ascertained*

-	Further detail is needed in terms of the 'calendar'

*As per above response, more details were provided in the data source part.*

-	The statement linking livebirths to birth history requires elaboration—clarify how neonatal death age was derived.

*As described in the data section, the CMC month of birth in calendar corresponds to CMC month of birth in the birth history. The DHS reports age of death in days, months and years*

-	Further explanation of the three data quality indicators and their role in model assessment is needed.

The role of each of the three data quality indicator in the model is provided in the method section (lines 200-259).

-	The description of inclusion/exclusion criteria should be clarified and summarised in a flow diagram.

*We added a supplementary table*

-	Replace wealth categories with explicit DHS wealth index tertiles/quintiles.

*We used wealth index tertiles, but label them as poor (first tertile), middle and rich (third tertile)*

-	The rationale for using Pearson correlations prior to model fitting should be explained

*We have used the Pearson correlation to examine whether these three data quality indicators vary by crude mortality rates and the covariates included in the models*

Results:

-The main results should more clearly emphasize the magnitude and direction of change over time, including specific time periods and annual percent change estimates with 95% CIs.

*From line 272 to 282, we amended as follows: Table 1 shows the stillbirth, early neonatal and perinatal mortality rates per 1 000 births for the first survey round and the last survey round in each country, and the country-specific trends over time were depicted on S1.1-S1.16 Fig. There is noticeable variation in the declines of all rates over time. The pooled annual rates of decline in stillbirth, early neonatal and perinatal mortality rates are depicted in Figures 1.1-1.3. The pooled annual decline rate in stillbirth is -0.62 (95%CI: -0.81,-0.43), and the decline was significant in eight out of 16 countries, the pooled annual decline of early neonatal mortality rate is -0.53 (95%CI: -0.69, -0.38), and the decline was significant in nine countries, the pooled annual decline of perinatal mortality rate is -1.13 (95%CI:-1.46, -0.80), and the decline was significant in nine countries. For the three rates, the largest declines were observed in Ghana and the smallest declines in Zimbabwe.*

-Please provide point estimates and reporting of results over statements such as 'There is a huge variation in the declines of both rates overtime, the largest decline was in Ghana and the smallest decline was in Kenya.'

*We revised as per above.*

-Results discuss change overtime but the time periods are not mentioned.

*We used survey specific time period that are referred to in the graphs*

-Two decimal places should be used for results. Please avoid using 3 decimal places to suggest significance (ie 0.995).

*We amended accordingly*

-Given the authors hold data on stillbirth and neonatal death (to create the perinatal mortality variable), why was neonatal death also not examined? The results of risk factors differ between stillbirth alone and perinatal mortality, this is an intriguing finding and suggest that some neonatal death risk factors differ to stillbirth. Thus, these results should be presented.

*We completely agree with the reviewer and now we added the trends in early neonatal mortality adjusting for transference and misclassification*

- it is unclear from the Figure 1 that this is average change in stillbirth per year, figure legends are needed for all figures and inclusion of whether these are adjusted or not. The figure would benefit from adding text to clarify that RR < 1 indicates a decline, while RR > 1 indicates an increase in stillbirth rates over time. Without this, the direction and meaning of the estimates is not intuitive.

*We amended accordingly*

Discussion

-The authors have not included any limitations within their study and have largely focused on comparison with previous studies.

*As per above, we have added a substantial paragraph on limitations.*

Reviewer #2: General Comments to authors This manuscript applies a modified Gompertz-Makeham model and the adjustment approach recently proposed by Ali et al. to 78 DHS reproductive-calendar surveys from 14 countries to estimate trends and sociodemographic differentials in stillbirth and perinatal mortality. The study uses a very large pooled dataset (785,695 pregnancies from 575,522 women) and provides useful country-level and pooled estimates, emphasizing the role of data-quality adjustment when household surveys are the main data source.

Major comments

1. Describe precisely how the Ali et al. adjustment was implemented: The manuscript refers to applying the approach of Ali et al., and uses the modified Gompertz-Makeham (mGM) model, but key implementation details are thin: how were cut-offs/thresholds chosen; which parameters were fixed vs estimated; how was model convergence assessed; what software / packages and code were used? Add a short algorithm/step list (survey-level fitting, adjustment, pooled meta-regression). Cite and explain any departures from Ali et al.

*Many thanks and as per above responses, the method section is now expanded to explain how the model was implemented, the cutoff points were based on Ali et al.*

2. Validation / model diagnostics and uncertainty: Present diagnostics for the mGM fits (e.g., goodness-of-fit plots, residuals, sensitivity to starting values), and for the meta-regression (heterogeneity measures I², τ²). Provide confidence intervals for country-specific trends and for pooled estimates throughout (you report pooled annual declines but the methods section must explain how CIs were computed). Figures 1.1/1.2 suggest CIs but please ensure numerical CIs appear in tables too.

*We provided confidence intervals and not uncertainty range since we are not fitting a Bayesian model. Heterogeneity measure are added to the forest plots.*

3. Survey design and weighting in meta-regression: The text states survey clustering and weights were considered; clarify precisely how DHS sampling weights were used in the survey-level life tables and in estimation of mGM parameters, and how uncertainty from complex survey design propagated into meta-analysis (were standard errors design-based?). This affects CIs and pooled inference.

*We used Stata survey commands for both the life-table and the models, the design-based standard error were used in the meta regression*

4. Missing data and exclusions: The manuscript mentions excluding pregnancies <7 months and some surveys (e.g., Colombia 1990) — provide a table listing included surveys with sample sizes, numbers of stillbirths and early neonatal deaths, and explicitly list excluded surveys with reasons (thresholds, missing calendar). This will help readers judge power and representativeness (E-Table 1 is referenced — ensure it contains this).

*A new table is provided*

5. Interpretation of covariate effects: Authors report pooled RRs by wealth, age, education and parity. Clarify whether these RRs are adjusted for other covariates (i.e., are model coefficients from the mGM model), and explain how pooling across countries with differing covariate distributions was handled. Consider presenting country-specific estimates in supplement and describing heterogeneity.

*All reported RRs are adjusted for other covariates in the mGM model. Surveys specific RRs are depicted in covariate specific forest plots*

6. Clarify HDI analyses: You report correlations between HDI and the outcomes; clarify whether HDI was treated as time-varying per survey and whether associations are ecological (survey-level) or individual-level. Explain the implication and risk of ecological fallacy.

*Country-specific HDI values correspond to the survey mid-year calendar, the association is ecological (at the survey-level)*

7. Provide code or reproducibility statement: The paper would benefit from a short reproducibility statement and (preferably) release of analysis scripts (or a DOI to a repo) so others can reproduce the adjustments and meta-analyses. This is increasingly expected for methods papers using public DHS data and complex adjustments.

*We are planning to provide all Stata codes for general use*

8. Tone down causal language: Many interpretations imply causality (e.g., link decline to specific policies). Rephrase to emphasize associations and plausible explanations unless formal causal inference is provided.

*Thanks for the suggestion, this now toned down*

Reviewer #3: This is an interesting paper, which brings a new statistical model to a crucial issue in reproductive health, i.e., stillbirths and perinatal mortality. The paper's main contribution is to apply a new statistical model t existing survey data to investigate trends in stillbirths in selected low and middle income countries. This new statistical model includes adjustments for data quality, to better reflect the underlying stillbirth rate, net of misclassifications and other reporting errors.

There are several issues that limit my enthusiasm for this paper:

1. The paper constantly refers to the Ali et al. model, without providing the required detail to understand the specific features of this model in the paper under review. We are only told "in brief" what this model does, without a discussion of how it improves on other models (e.g., IGME, GBD) and what might be its limitations and assumptions. Without reiterating the Ali et al paper in the Lancet GH, we should have more details in this paper.

*We believe sufficient details are not provided in the method section*

2. The paper argues that one of its key innovations is to study trends in stillbirths, which is otherwise "rarely undertaken". However, there are investigations of trends in stillbirths, for example GBD 2021. This work is not cited, and the authors should discuss how their approach improves on other attempts to examine trends in stillbirths.

*Many thanks for this important comment and have now expanded the discussion including additional papers. An additional paragraph with new references reads as follows: In terms of education, our analysis did not reveal a significant relationship between maternal education and stillbirth rates, which contrasts with findings from a multi-country cross-sectional study indicating that low maternal education (OR: 1.50, 95% CI: 1.01–2.24) is significantly associated with increased stillbirth risk [24]. However, we found stronger alignment with wealth status, where higher financial security correlated with reduced stillbirth and early neonatal mortality. Previous studies have consistently shown that infants born to mothers with lower educational attainment or living in rural settings experience higher risks of early neonatal mortality, largely due to disparities in health literacy, care-seeking behavior, and access to skilled obstetric and neonatal services [25,26]. In contrast, stillbirths are often more strongly linked to biological and obstetric conditions, including placental dysfunction, hypertensive disorders of pregnancy, infections, fetal growth restriction, and congenital anomalies [27]. Global analyses highlight that many stillbirths occur due to medical complications during pregnancy or labor rather than postnatal care practices [27].*

3. In examining trends using their new model, a key assumption is whether the adjustments detailed in the Ali et al. Lancet GH paper are fixed over time, or are allowed to vary. At first sight, it seems the authors consider the relations between data quality indicators and adjustments as fixed over time, but I could not really find detail about this. Yet, the trends in stillbirths could be more or less steep, depending on this. The authors should discuss this issue in their data and methods, and/or in the discussion.

*The adjustment is not fixed over time, the thresholds are assessed against the data quality in each survey and adjustment accordingly*

4. In fact, the paper does not include any mention of limitations. It would be important to mention some of those in the discussion, including reiterating some of the limitations of the model discussed in the Lancet GH piece.

*As per above we added a substantial limitation part.*

5. Both the current submission and the Lancet GH paper ignore important literature in demography on the measurement of neonatal mortality, including early neonatal mortality. For example, the work of M. Guillot's group, for example:

*As per above response, we added most of the following references*

<https://journals.plos.org/plosone/article?id=10.1371/journal.pone.0304841>

<https://read.dukeupress.edu/demography/article/59/1/321/293847/Modeling-Age-Patterns-of-Under-5-Mortality-Results>

or another study that validated data produced by the DHS questionnaire in Guinea-Bissau: <https://read.dukeupress.edu/demography/article/57/5/1705/168374/Biases-in-Survey-Estimates-of-Neonatal-Mortality>

Taken together, these studies present important challenges to the measurement of early neonatal mortality, which should be discussed in this paper.

6. For the general reader, it might be good to define D0-D1, and other ratios.

*Good point, it is now done.*
