# Supplementary material for: Trends of stillbirth, early neonatal and perinatal mortality rates and sociodemographic differentials of perinatal mortality rates in 17 low- and middle-income countries: A modeling study
Source: PLoS Med. 2026 Aug 24;23(8):e1004791. doi: 10.1371/journal.pmed.1004791 (PMC13502698; doi:10.1371/journal.pmed.1004791)
Supplement: Supplementary file 13 [file pmed.1004791.s014.docx]

Reviewer #1: I thank the authors for their considered update of the manuscript and detail of methods, this has strengthen the manuscript. A few additional points:

* Importantly, the validity of the estimates relies on assumptions regarding the relationship between stillbirth and early neonatal mortality (e.g. Stillbirth:D0-1 ratio = 1.89; threshold = 2.4), which may not hold across settings. Accordingly, the results should be interpreted as model-based, calibrated estimates rather than direct measures.

*Many thanks and we added this aspect at the end of the limitations paragraph in the discussion.*

* Although the authors do not see the need for sensitivity analysis this should be considered given that key components of the model rely on imposed parameters (as stated above), sensitivity analyses exploring alternative specifications would be important to assess the robustness of the findings. At a minimum the reliance on assumptions should be acknowledged as a limitation.

*Many thanks and we included a sensitivity analysis. Specifically, we included the following paragraph to the end of methods sections: “As an alternative to the original assumption used to address omission and under-reporting, we applied a less restrictive approach (sensitivity analysis). Instead of assigning a fixed value of 1.89 to the STB/D0–1 ratio when the observed value fell below this threshold, we used a plausible range derived from Table 1 in Ali et al., we assigned 1.89 if the Observed STB/D0–1 ratios below 1.7 or above 3.0; values within this range were left unchanged.  A similar approach was used for the D0–1/D2–6 ratio. Rather than applying a fixed cutoff of 2.4, we adopted the bounds reported in E-Table A2.4 of the supplementary materials in Ali et al. Ratios below 1.6 or above 3.7 were set to 2.4, while values within the range were retained. The country-specific stillbirth, early neonatal and perinatal mortality rates with their 95%CIs that are based on the original and the alternative assumptions are depicted in (S4 Fig). The median and the lower quartile differences are zero (S5 Fig).”*

* The authors refer to the importance of using this approach to correct for inaccurate reporting throughout the manuscript but do not show the trends using raw/uncorrected data. This is a missing piece of critical information to substantiate their claims.

*Many thanks for this comment. The survey-specific observed and adjusted rates, and age-specific risk of death and cumulative probabilities were in the* [*appendix (pp 37, 41*](https://www.thelancet.com/journals/langlo/article/PIIS2214-109X(23)00125-0/fulltext#supplementary-material)*) of the LANCET paper by Ali et al (*[*Measuring stillbirth and perinatal mortality rates through household surveys: a population-based analysis using an integrated approach to data quality assessment and adjustment with 157 surveys from 53 countries - The Lancet Global Health*](https://www.thelancet.com/journals/langlo/article/PIIS2214-109X(23)00125-0/fulltext#supplementary-material)).

*For this paper, we developed country charts plotting the model trends and the proposed trends using the sensitivity analysis.*

* The presentation of trends are clear (supplemental figure 1) but does show smooth trends due to modelling assumptions. Please add in the figure legend that states these are model based estimates.

*Many thanks and we added as recommended: we explicitly added “this are adjusted estimates”.*

* Abstract conclusion state that adjusting for data quality substantially improves estimates but this is not shown in abstract results.

*We provided more emphasis on the robustness of the findings considering the sensitivity analysis. Therefore, we amended the abstract as follows: “….The pooled annual decline was −0.60 deaths per 1,000 births for stillbirths (95% CI −0.78 to −0.41), −0.50 for early neonatal mortality (95% CI −0.66 to −0.35), and −1.08 for perinatal mortality (95% CI −1.40 to −0.76). A sensitivity analysis using alternate assumptions confirmed the robustness of the findings at country level…”*

***Conclusions***

*Adjusting for data quality issues is a robust modality to improve the use of household survey data for estimating stillbirth and early neonatal mortality…”*

**We have also created the below repository to upload the Stata codes for public use**

[**https://github.com/MohamedMahmoud2512/Perinatal-mortality.git**](https://github.com/MohamedMahmoud2512/Perinatal-mortality.git)
