# Supplementary material for: Trends of stillbirth, early neonatal and perinatal mortality rates and sociodemographic differentials of perinatal mortality rates in 17 low- and middle-income countries: A modeling study
Source: PLoS Med. 2026 Aug 24;23(8):e1004791. doi: 10.1371/journal.pmed.1004791 (PMC13502698; doi:10.1371/journal.pmed.1004791)
Supplement: Supplementary file 14 [file pmed.1004791.s015.docx]

# Response to Editors – PLOS Medicine

Dear Editors,

We thank you for your careful assessment of our manuscript and for the constructive suggestions provided. We have revised the manuscript extensively and addressed all editorial comments. Below we provide a point-by-point response indicating how each issue was addressed in the revised manuscript.

EDITORIAL LINE EDITS

1. Heaping clarified for general readers: we added a plain-language definition explaining age-at-death heaping as the tendency to misreport neonatal deaths at specific ages (often 7 days).

2. Claim of robust modality tempered: revised to state that adjusting for data quality issues may potentially improve the use of household survey data.

3. Comma inserted after “neonatal mortality data”.

4. First Results paragraph revised for accessibility and readability.

5. Active voice used throughout the manuscript wherever appropriate.

6. Additional explanations added describing how statistical adjustment affects mortality estimates and trends.

7. Technical wording regarding the heaping index and D0–1/D2–6 ratio rewritten in plain language.

8. Repeated phrase “of the” corrected.

9. “Strong correlations” replaced with the more objective term “statistically significant correlations”.

10. “Politically” replaced with “political”.

11. Author statement revised to: “Mohamed M Ali is a former WHO staff member and currently a freelance data scientist.”

12. S2 Table title changed to “Excluded surveys”.

13. Figure numbering revised and corresponding callouts updated.

14. Justification for inclusion of the 17 countries added to the Data Source section.

15. Sociodemographic differentials are now discussed more extensively in the Results and Discussion, while the title was streamlined.


ABSTRACT

The abstract has been revised to follow the required structure (Background, Methods and Findings, Conclusions) and includes study design, key findings and limitations.

INTRODUCTION

The Introduction now ends with a clear statement of the study objectives.

ABBREVIATIONS

All abbreviations are defined at first use.

ABSTRACT–MANUSCRIPT CONSISTENCY

All numerical values reported in the abstract were checked against the main manuscript and verified for consistency.

AUTHOR SUMMARY

The author summary now includes the sociodemographic analyses and a plain-language description of study limitations.

FUNDING

The study did not receive specific funding. The financial disclosure statement was revised accordingly.

COMPETING INTERESTS

Competing interest disclosures were reviewed and updated in line with PLOS policy.

DATA AVAILABILITY

The Data Availability Statement was revised to clarify data access through the DHS Program. Supporting data underlying figures and tables will be provided.

CODE AVAILABILITY

The statistical code will be deposited in a public repository and cited through a persistent identifier in the revised submission.

FIGURES AND TABLES

Titles, legends and acronym definitions have been reviewed and completed. Covariates included in adjusted models are clearly specified.

INCLUSIVITY IN GLOBAL RESEARCH

The required PLOS inclusivity questionnaire will be submitted with the revised manuscript.

MODELING STUDY REQUIREMENTS

The Methods section was strengthened with additional detail regarding model structure, assumptions, fitting procedures, uncertainty assessment, sensitivity analyses, and model rationale. These elements are now described more clearly in the manuscript.

We are grateful to the Editors for these insightful comments, which have significantly improved the clarity, accessibility, and reporting of the manuscript.

Sincerely,

Mohamed M. Ali
Luca Cegolon
Saverio Bellizzi
