# Supplementary material for: Trends of stillbirth, early neonatal and perinatal mortality rates and sociodemographic differentials of perinatal mortality rates in 17 low- and middle-income countries: A modeling study
Source: PLoS Med. 2026 Aug 24;23(8):e1004791. doi: 10.1371/journal.pmed.1004791 (PMC13502698; doi:10.1371/journal.pmed.1004791)
Supplement: Supplementary file 15 [file pmed.1004791.s016.docx]

# Response to Editors – PLOS Medicine

Dear Editor,

We thank you for your careful assessment of our manuscript and for the constructive suggestions provided. We have revised the manuscript extensively and addressed all editorial comments. Below we provide a point-by-point response indicating how each issue was addressed in the revised manuscript.

DATA AVAILABILITY STATEMENT
1) thank you for including the data underlying the figures. You need to include the following statement (or similar) in your Data availability statement in the meta-data:
"data underlying the graphs and the supplementary figures can be found in ‘supplementary data for Figures".

- Thanks and it is now done

2) Thank you for providing the Github link. However.
 -We also need a DOI, e.g. via Zenodo.
  -Please include the following statement in you data availability statement in the metadata: "The code files underlying the results of the study are available from the Github code repository (<https://github.com/SindisoNyathi/California-Vaccine-Policy>) and [include information on the DOI here].

- Thanks and it is now done

3) Thank you for amending the sensitivity analysis section of the methods. However, the included reference is not in the PLOS style. Please amend this.

- Many thanks and it now reads: *Instead of applying the fixed thresholds used in the primary model, we adopted plausible ranges reported by Ali et al. for the stillbirth-to-D0–1 and D0–1-to-D2–6 ratios [7].*

4) One of our comments in the discussion was not addressed:
Line 412 (now line 433): ‘survey value’ what do you mean here, please clarify. Also consider whether ‘value’ needs to be plural.  You explained this in the rebuttal, but not in the paper. this explanation needs to be included in the manuscript.

- Many thanks and we replaces “value” with “figures”

5) Figure 1-3:
Thank you for amending these figures. However, currently ‘changes’ and "[95% confidence interval]" are overlapping in these figures.
Please change to ‘CI’ in the figure, and provide the abbreviation in the legend/footnote to the figures. *(decline) overtime.*

- We amended accordingly and uploaded new figures
