# Supplementary material for: Trends of stillbirth, early neonatal and perinatal mortality rates and sociodemographic differentials of perinatal mortality rates in 17 low- and middle-income countries: A modeling study
Source: PLoS Med. 2026 Aug 24;23(8):e1004791. doi: 10.1371/journal.pmed.1004791 (PMC13502698; doi:10.1371/journal.pmed.1004791)
Supplement: Peer review history

# Editor report: Decision Letter 0

**DOI:** 10.1371/journal.pmed.1004791.r001

**Authors:** Heather Van Epps

12 Oct 2025

Dear Dr Bellizzi,

Thank you for submitting your manuscript entitled "Trends and sociodemographic differentials of stillbirth and perinatal mortality rates in 14 low- and middle-income countries: an application of modified Gompertz-Makeham model" for consideration by PLOS Medicine.

Your manuscript has now been evaluated by the PLOS Medicine editorial staff as well as by an academic editor with relevant expertise and I am writing to let you know that we would like to send your submission out for external peer review.

However, before we can send your manuscript to reviewers, we need you to complete your submission by providing the metadata that is required for full assessment. To this end, please login to Editorial Manager where you will find the paper in the 'Submissions Needing Revisions' folder on your homepage. Please click 'Revise Submission' from the Action Links and complete all additional questions in the submission questionnaire.

For clinical studies, please upload a copy of your trial study protocol as a supporting information file. The study protocol should be the version submitted for approval to the institutional review board or ethics committee, should include any amendments to the study protocol, as well as the date of their approval by the institutional review or ethics committee. Please also detail any deviations from the study protocol in the Methods section of your manuscript. The editors will consider the protocol and study conduct prior to a final decision for external review.

Please re-submit your manuscript within two working days, i.e. by Oct 14 2025 11:59PM.

Login to Editorial Manager here: https://www.editorialmanager.com/pmedicine

Once your full submission is complete, your paper will undergo a series of checks in preparation for peer review. Once your manuscript has passed all checks it will be sent out for review.

Feel free to email us at plosmedicine@plos.org if you have any queries relating to your submission.

Kind regards,

Heather Van Epps, PhD

Consulting Editor

PLOS Medicine

# Peer review: Decision Letter 1

**DOI:** 10.1371/journal.pmed.1004791.r002

**Authors:** Suzanne De Bruijn

3 Dec 2025

Dear Dr Bellizzi,

Many thanks for submitting your manuscript "Trends and sociodemographic differentials of stillbirth and perinatal mortality rates in 14 low- and middle-income countries: an application of modified Gompertz-Makeham model" (PMEDICINE-D-25-03514R1) to PLOS Medicine. The paper has been reviewed by subject experts and a statistician; their comments are included below and can also be accessed here: [LINK]

As you will see, the reviewers showed interest in your manuscript, but also voiced several concerns. The concerns included not enough detail provided on the methodology and existing literature, as well as a lack of discussion of limitations. additional analyses were also requested. After discussing the paper with the editorial team and an academic editor with relevant expertise, I'm pleased to invite you to revise the paper in response to the reviewers' comments. We plan to send the revised paper to some or all of the original reviewers, and we cannot provide any guarantees at this stage regarding publication.

In addition to these revisions, you may need to complete some formatting changes, which you will receive in a follow up email. A member of our team will be in touch with a set of requests shortly. If you do not receive a separate email within a few days, please assume that checks have been completed, and no additional changes are required.

When you upload your revision, please include a point-by-point response that addresses all of the reviewer and editorial points, indicating the changes made in the manuscript and either an excerpt of the revised text or the location (eg: page and line number) where each change can be found. Please also be sure to check the general editorial comments at the end of this letter and include these in your point-by-point response. When you resubmit your paper, please include a clean version of the paper as the main article file and a version with changes tracked as a marked-up manuscript. It may also be helpful to check the guidelines for revised papers at http://journals.plos.org/plosmedicine/s/revising-your-manuscript for any that apply to your paper.

We ask that you submit your revision by Dec 24 2025 11:59PM. However, if this deadline is not feasible, please contact me by email, and we can discuss a suitable alternative.

Don't hesitate to contact me directly with any questions (sbruijn@plos.org).

Best regards,

Suzanne

Suzanne De Bruijn, PhD

Associate Editor

PLOS Medicine

sbruijn@plos.org

-----------------------------------------------------------

Comments from the reviewers:

Reviewer #1: This is an interesting paper that draws on a recently described method by the same team to examine trends of stillbirth and neonatal death over time. They have used a Gompertz-Makeham model, which aims to adjust for misreporting/transfer bias. However, as written, the manuscript is difficult to follow, the analytical approach is not sufficiently transparent, and several key methodological and interpretative issues require clarification before the findings can be reliably interpreted. In particular, the description of how trends over time were derived, and how misclassification adjustments influence those trends, requires clearer explanation and sensitivity analyses.

Abstract: Further detail of the Gompertz-Makeham model is needed to give context to the paper The reproductive calendar should be briefly defined, including acknowledgement of known under-reporting of stillbirth and misclassification to neonatal deaths. Abstract results should include point estimates. Define income rather than rich/poor. This sentence is unclear 'however for prenatal mortality all covariates examined were significant associated with perinatal mortality.' The objective of the study and difference to the previous study needs more clarity.

Introduction:

-Definition of neonatal death is incorrect - should be up to 28 days and should state WHO definition of stillbirth.

-Additional background on DHS surveys is warranted, including their central role in producing global perinatal mortality estimates and their known limitations (recall error, misclassification).

-More detail describing the prior study introducing the mGM approach would help establish rationale.

Methods:

Overall, the objectives, and use of the mGM model are not described with sufficient clarity for reproducibility.

-The methods do not state the objective of the study and overall are difficult to follow.

-The approach used to estimate trends over time must be clarified. It is not clear whether calendar time was modelled as a continuous variable, a categorical survey indicator. The model specification should include the time term.

-Because the mGM model includes misclassification constraints, uncertainty from these adjustments should be assessed in sensitivity analyses

-It's not stated what years of births the surveys represent in methods, this should be included for each and does impact the applicability of the findings, especially for data >10 years old.

-Using gestational age coded in months should be acknowledged as a limitation due to possible misclassification of stillbirth eligibility.

-The assumption that births occurring in the month of interview are >1 week old may underestimate deaths on day 0-1; please report whether a sensitivity analysis was conducted assuming alternative ages.

-Please describe how multiple births were handled in both numerators and denominators.

-Further detail is needed in terms of the 'calendar'

-The statement linking livebirths to birth history requires elaboration—clarify how neonatal death age was derived.

- Further explanation of the three data quality indicators and their role in model assessment is needed.

- The description of inclusion/exclusion criteria should be clarified and summarised in a flow diagram.

- Replace wealth categories with explicit DHS wealth index tertiles/quintiles.

- The rationale for using Pearson correlations prior to model fitting should be explained

Results:

-The main results should more clearly emphasize the magnitude and direction of change over time, including specific time periods and annual percent change estimates with 95% CIs.

-Please provide point estimates and reporting of results over statements such as 'There is a huge variation in the declines of both rates overtime, the largest decline was in Ghana and the smallest decline was in Kenya.'

-Results discuss change overtime but the time periods are not mentioned.

-Two decimal places should be used for results. Please avoid using 3 decimal places to suggest significance (ie 0.995).

-Given the authors hold data on stillbirth and neonatal death (to create the perinatal mortality variable), why was neonatal death also not examined? The results of risk factors differ between stillbirth alone and perinatal mortality, this is an intriguing finding and suggest that some neonatal death risk factors differ to stillbirth. Thus, these results should be presented.

- it is unclear from the Figure 1 that this is average change in stillbirth per year, figure legends are needed for all figures and inclusion of whether these are adjusted or not. The figure would benefit from adding text to clarify that RR < 1 indicates a decline, while RR > 1 indicates an increase in stillbirth rates over time. Without this, the direction and meaning of the estimates is not intuitive.

-Figures should include the n/N for each country

Discussion

-The authors have not included any limitations within their study and have largely focused on comparison with previous studies.

Reviewer #2: General Comments to authors

This manuscript applies a modified Gompertz-Makeham model and the adjustment approach recently proposed by Ali et al. to 78 DHS reproductive-calendar surveys from 14 countries to estimate trends and sociodemographic differentials in stillbirth and perinatal mortality. The study uses a very large pooled dataset (785,695 pregnancies from 575,522 women) and provides useful country-level and pooled estimates, emphasizing the role of data-quality adjustment when household surveys are the main data source.

Major comments

1.Describe precisely how the Ali et al. adjustment was implemented: The manuscript refers to applying the approach of Ali et al., and uses the modified Gompertz-Makeham (mGM) model, but key implementation details are thin: how were cut-offs/thresholds chosen; which parameters were fixed vs estimated; how was model convergence assessed; what software / packages and code were used? Add a short algorithm/step list (survey-level fitting, adjustment, pooled meta-regression). Cite and explain any departures from Ali et al.

2.Validation / model diagnostics and uncertainty: Present diagnostics for the mGM fits (e.g., goodness-of-fit plots, residuals, sensitivity to starting values), and for the meta-regression (heterogeneity measures I², τ²). Provide confidence intervals for country-specific trends and for pooled estimates throughout (you report pooled annual declines but the methods section must explain how CIs were computed). Figures 1.1/1.2 suggest CIs but please ensure numerical CIs appear in tables too.

1.Survey design and weighting in meta-regression: The text states survey clustering and weights were considered; clarify precisely how DHS sampling weights were used in the survey-level life tables and in estimation of mGM parameters, and how uncertainty from complex survey design propagated into meta-analysis (were standard errors design-based?). This affects CIs and pooled inference. .

2.Handling of interval censoring and linkage rules: More detail on data restructuring: how were month-reported stillbirths and day-reported neonatal deaths aligned; how were ambiguous month/day records treated; how were current pregnancies censored exactly? These choices can materially affect results—please expand and, if possible, provide a short flowchart or pseudocode. .

3.Missing data and exclusions: The manuscript mentions excluding pregnancies <7 months and some surveys (e.g., Colombia 1990) — provide a table listing included surveys with sample sizes, numbers of stillbirths and early neonatal deaths, and explicitly list excluded surveys with reasons (thresholds, missing calendar). This will help readers judge power and representativeness (E-Table 1 is referenced — ensure it contains this). .

4.Potential recall bias and residual misclassification: Although the model adjusts for underreporting and misclassification, recall bias in long reproductive calendars (especially for older births) and differential reporting by socioeconomic group may remain. Discuss potential direction and magnitude of biases and whether sensitivity analyses by recall window (e.g., last 5 years vs earlier) were performed or possible. This is important for claims about sociodemographic differentials.

5.Interpretation of covariate effects: Authors report pooled RRs by wealth, age, education and parity. Clarify whether these RRs are adjusted for other covariates (i.e., are model coefficients from the mGM model), and explain how pooling across countries with differing covariate distributions was handled. Consider presenting country-specific estimates in supplement and describing heterogeneity. .

6.Clarify HDI analyses: You report correlations between HDI and the outcomes; clarify whether HDI was treated as time-varying per survey and whether associations are ecological (survey-level) or individual-level. Explain the implication and risk of ecological fallacy.

7.Provide code or reproducibility statement: The paper would benefit from a short reproducibility statement and (preferably) release of analysis scripts (or a DOI to a repo) so others can reproduce the adjustments and meta-analyses. This is increasingly expected for methods papers using public DHS data and complex adjustments.

8.Tone down causal language: Many interpretations imply causality (e.g., link decline to specific policies). Rephrase to emphasize associations and plausible explanations unless formal causal inference is provided.

Reviewer #3: This is an interesting paper, which brings a new statistical model to a crucial issue in reproductive health, i.e., stillbirths and perinatal mortality. The paper's main contribution is to apply a new statistical model t existing survey data to investigate trends in stillbirths in selected low and middle income countries. This new statistical model includes adjustments for data quality, to better reflect the underlying stillbirth rate, net of misclassifications and other reporting errors.

There are several issues that limit my enthusiasm for this paper:

1. The paper constantly refers to the Ali et al. model, without providing the required detail to understand the specific features of this model in the paper under review. We are only told "in brief" what this model does, without a discussion of how it improves on other models (e.g., IGME, GBD) and what might be its limitations and assumptions. Without reiterating the Ali et al paper in the Lancet GH, we should have more details in this paper.

2. The paper argues that one of its key innovations is to study trends in stillbirths, which is otherwise "rarely undertaken". However, there are investigations of trends in stillbirths, for example GBD 2021. This work is not cited, and the authors should discuss how their approach improves on other attempts to examine trends in stillbirths.

3. In examining trends using their new model, a key assumption is whether the adjustments detailed in the Ali et al. Lancet GH paper are fixed over time, or are allowed to vary. At first sight, it seems the authors consider the relations between data quality indicators and adjustments as fixed over time, but I could not really find detail about this. Yet, the trends in stillbirths could be more or less steep, depending on this. The authors should discuss this issue in their data and methods, and/or in the discussion.

4. In fact, the paper does not include any mention of limitations. It would be important to mention some of those in the discussion, including reiterating some of the limitations of the model discussed in the Lancet GH piece.

5. Both the current submission and the Lancet GH paper ignore important literature in demography on the measurement of neonatal mortality, including early neonatal mortality. For example, the work of M. Guillot's group, for example:

https://journals.plos.org/plosone/article?id=10.1371/journal.pone.0304841

https://read.dukeupress.edu/demography/article/59/1/321/293847/Modeling-Age-Patterns-of-Under-5-Mortality-Results

or another study that validated data produced by the DHS questionnaire in Guinea-Bissau: https://read.dukeupress.edu/demography/article/57/5/1705/168374/Biases-in-Survey-Estimates-of-Neonatal-Mortality

Taken together, these studies present important challenges to the measurement of early neonatal mortality, which should be discussed in this paper.

6. For the general reader, it might be good to define D0-D1, and other ratios.

Any attachments provided with reviews can be seen via the following link: [LINK]

--------------------------------------------------------- ---

General editorial requests:

*We ask every co-author listed on the manuscript to fill in a contributing author statement, making sure to declare all competing interests. If any of the co-authors have not filled in the statement, we will remind them to do so when the paper is revised. If all statements are not completed in a timely fashion this could hold up the re-review process. If new competing interests are declared later in the revision process, this may also hold up the submission. Should there be a problem getting one of your co-authors to fill in a statement we will be in contact. Please do not add or remove authors without first discussing this with the handling editor. You can see our competing interests policy here: http://journals.plos.org/plosmedicine/s/competing-interests.

*Please upload any figures associated with your paper as individual TIF or EPS files with 300dpi resolution at resubmission; please read our figure guidelines for more information on our requirements: http://journals.plos.org/plosmedicine/s/figures. While revising your submission, we strongly recommend that you use PLOS's NAAS tool (https://ngplosjournals.pagemajik.ai/artanalysis) to test your figure files. NAAS can convert your figure files to the TIFF file type and meet basic requirements (such as print size, resolution), or provide you with a report on issues that do not meet our requirements and that NAAS cannot fix.

After uploading your figures to PLOS's NAAS tool - https://ngplosjournals.pagemajik.ai/artanalysis, NAAS will process the files provided and display the results in the "Uploaded Files" section of the page as the processing is complete.

If the uploaded figures meet our requirements (or NAAS is able to fix the files to meet our requirements), the figure will be marked as "fixed" above. If NAAS is unable to fix the files, a red "failed" label will appear above.

When NAAS has confirmed that the figure files meet our requirements, please download the file via the download option, and include these NAAS processed figure files when submitting your revised manuscript.

*Please ensure that the paper adheres to the PLOS Data Availability Policy (see http://journals.plos.org/plosmedicine/s/data-availability), which requires that all data underlying the study's findings be provided in a repository or as Supporting Information. For data residing with a third party, authors are required to provide instructions with contact information (web or email address) for obtaining the data. Please note that a study author cannot be the contact person for the data. PLOS journals do not allow statements supported by "data not shown" or "unpublished results." For such statements, authors must provide supporting data or cite public sources that include it.

* Please amend your Data availability statement in the metadata, to clearly state how researchers can obtain the used data. Please also provide the analysis files as supplementary files, rather than stating they can be obtained upon request.

FORMATTING - GENERAL

*Abstract: Please structure your abstract using the PLOS Medicine headings (Background, Methods and Findings, Conclusions). Please combine the Methods and Findings sections into one section.

*At this stage, we ask that you include a short, non-technical Author Summary of your research to make findings accessible to a wide audience that includes both scientists and non-scientists. The Author Summary should immediately follow the Abstract in your revised manuscript. This text is subject to editorial change and should be distinct from the scientific abstract. Ideally each sub-heading should contain 2-3 single sentence, concise bullet points containing the most salient points from your study. In the final bullet point of 'What Do These Findings Mean?', please include the main limitations of the study in non-technical language. Please see our author guidelines for more information: https://journals.plos.org/plosmedicine/s/revising-your-manuscript#loc-author-summary.

*Please express the main results with 95% CIs as well as p values. When reporting p values please report as p<0.001 and where higher as the exact p value p=0.002, for example. Throughout, suggest reporting statistical information as follows to improve clarity for the reader "22% (95% CI [13%,28%]; p</=)". Please be sure to define all numerical values at first use.

*Please include page numbers and line numbers in the manuscript file. Use continuous line numbers (do not restart the numbering on each page).

*Please cite the reference numbers in square brackets. Citations should precede punctuation.

FIGURES AND TABLES

*Please provide titles and legends for all figures and tables (including those in Supporting Information files).

*Please define all abbreviations used in each figure/table (including those in Supporting Information files).

SUPPLEMENTARY MATERIAL

*Please note that supplementary material will be posted as supplied by the authors. Therefore, please amend it according to the relevant comments outlined here.

*Please cite your Supporting Information as outlined here: https://journals.plos.org/plosmedicine/s/supporting-information

REFERENCES

*PLOS uses the numbered citation (citation-sequence) method and first six authors, et al.

*Please ensure that journal name abbreviations match those found in the National Center for Biotechnology Information (NCBI) databases (http://www.ncbi.nlm.nih.gov/nlmcatalog/journals), and are appropriately formatted and capitalised.

*Where website addresses are cited, please include the complete URL and specify the date of access (e.g. [accessed: 12/06/2023]).

*Please also see https://journals.plos.org/plosmedicine/s/submission-guidelines#loc-references for further details on reference formatting.

OBSERVATIONAL STUDIES

*Abstract: Please include the study design, population and setting, number of participants, years during which the study took place (enrollment and follow up), length of follow up, and main outcome measures.

*Please ensure that the study is reported according to the GATHER statement (available from https://www.equator-network.org/reporting-guidelines/gather-statement) and include the completed checklist as Supporting Information. Please add the following statement, or similar, to the Methods: "This study is reported as per the Guidelines for Accurate and Transparent Health Estimates Reporting (GATHER) statement (S1 Checklist)." When completing the checklist, please use section and paragraph numbers, rather than page numbers.

*For all observational studies, in the manuscript text, please indicate: (1) the specific hypotheses you intended to test, (2) the analytical methods by which you planned to test them, (3) the analyses you actually performed, and (4) when reported analyses differ from those that were planned, transparent explanations for differences that affect the reliability of the study's results. If a reported analysis was performed based on an interesting but unanticipated pattern in the data, please be clear that the analysis was data driven.

*Please state in the Methods section whether the study had a prospective protocol or analysis plan. If a prospective analysis plan (from your funding proposal, IRB or other ethics committee submission, study protocol, or other planning document written before analyzing the data) was used in designing the study, please include the relevant document(s) with your revised manuscript as a Supporting Information file to be published alongside your study and cite it in the Methods section. A legend for this file should be included at the end of your manuscript. If no such document exists, please make sure that the Methods section transparently describes when analyses were planned, and when/why any data-driven changes to analyses took place. Changes in the analysis, including those made in response to peer review comments, should be identified as such in the Methods section of the paper, with rationale.

# Author comment: Author response to Decision Letter 2

**DOI:** 10.1371/journal.pmed.1004791.r003

17 Mar 2026

Attachment Submitted filename: point by point PLOS.docx

# Peer review: Decision Letter 2

**DOI:** 10.1371/journal.pmed.1004791.r004

**Authors:** Suzanne De Bruijn

22 Apr 2026

Dear Dr Bellizzi,

Many thanks for submitting a revised version of your manuscript "Trends of stillbirth, early neonatal and perinatal mortality rates and sociodemographic differentials of perinatal mortality rates in 16 low- and middle-income countries: an application of modified Gompertz-Makeham model" (PMEDICINE-D-25-03514R2) to PLOS Medicine. The paper has been seen again by the original reviewers; their comments are included below and can also be accessed here: [LINK]

As you will see, two of the reviewers are satisfied with the responses, whereas reviewer #1 has some remaining concerns. After discussing the paper with the editorial team and an academic editor with relevant expertise, I'm pleased to invite you to revise the paper in response to the reviewers' comments. Please note that we will require the sensitivity analyses that are requested during review, and acknowledging the reliance on assumptions will not be sufficient. We plan to send the revised paper to reviewer #1, and we cannot provide any guarantees at this stage regarding publication.

In addition to these revisions, you may need to complete some formatting changes, which you will receive in a follow up email. A member of our team will be in touch with a set of requests shortly. If you do not receive a separate email within a few days, please assume that checks have been completed, and no additional changes are required.

When you upload your revision, please include a point-by-point response that addresses all of the reviewer and editorial points, indicating the changes made in the manuscript and either an excerpt of the revised text or the location (eg: page and line number) where each change can be found. Please also be sure to check the general editorial comments at the end of this letter and include these in your point-by-point response. When you resubmit your paper, please include a clean version of the paper as the main article file and a version with changes tracked as a marked-up manuscript. It may also be helpful to check the guidelines for revised papers at http://journals.plos.org/plosmedicine/s/revising-your-manuscript for any that apply to your paper.

We ask that you submit your revision by May 13 2026 11:59PM. However, if this deadline is not feasible, please contact me by email, and we can discuss a suitable alternative.

Don't hesitate to contact me directly with any questions (sbruijn@plos.org).

Best regards,

Suzanne

Suzanne De Bruijn, PhD

Associate Editor

PLOS Medicine

sbruijn@plos.org

-----------------------------------------------------------

Comments from the reviewers:

Reviewer #1: I thank the authors for their considered update of the manuscript and detail of methods, this has strengthen the manuscript. A few additional points:

*Importantly, the validity of the estimates relies on assumptions regarding the relationship between stillbirth and early neonatal mortality (e.g. Stillbirth:D0-1 ratio = 1.89; threshold = 2.4), which may not hold across settings. Accordingly, the results should be interpreted as model-based, calibrated estimates rather than direct measures.

*Although the authors do not see the need for sensitivity analysis this should be considered given that key components of the model rely on imposed parameters (as stated above), sensitivity analyses exploring alternative specifications would be important to assess the robustness of the findings. At a minimum the reliance on assumptions should be acknowledged as a limitation.

*The authors refer to the importance of using this approach to correct for inaccurate reporting throughout the manuscript but do not show the trends using raw/uncorrected data. This is a missing piece of critical information to substantiate their claims.

*The presentation of trends are clear (supplemental figure 1) but does show smooth trends due to modelling assumptions. Please add in the figure legend that states these are model based estimates.

*Abstract conclusion state that adjusting for data quality substantially improves estimates but this is not shown in abstract results.

Reviewer #2: Thank you for addressing all my comments.

Reviewer #3: Comments have been well considered and addressed.

Any attachments provided with reviews can be seen via the following link: [LINK]

--------------------------------------------------------- ---

General editorial requests:

* Please ensure that the paper adheres to the PLOS Data Availability Policy (see http://journals.plos.org/plosmedicine/s/data-availability), which requires that all data underlying the study's findings be provided in a repository or as Supporting Information. For data residing with a third party, authors are required to provide instructions with contact information (web or email address) for obtaining the data. Please note that a study author cannot be the contact person for the data. PLOS journals do not allow statements supported by "data not shown" or "unpublished results." For such statements, authors must provide supporting data or cite public sources that include it.

* Please amend your Data availability statement in the metadata, to clearly state how researchers can obtain the used data. stating 'upon request' is not sufficient, please state what researchers need to do in order to get access. Please also provide the analysis files as supplementary files, rather than stating they can be obtained upon request.

* AUTHOR SUMMARY: Thank you for including an author summary. Please include the main limitations in the final bullet point of 'what do these findings mean'.

* Statistical reporting: Please revise throughout the manuscript, including tables and figures.

- Please report statistical information as follows to improve clarity for the reader ""22% (95% CI [13,28]; p</=)"".

- Please separate upper and lower bounds with commas instead of hyphens as the latter can be confused with reporting of negative values.

- Please repeat statistical definitions (HR, CI etc.) for each set of parentheses.

* For studies in which a novel model is central to the manuscript's findings, as is the case here, authors are responsible for providing the source code needed to replicate the study's findings in a repository (such as GitHub, SourceForge or Bitbucket) or a cloud computing service (such as Code Ocean). Protection of authors’ intellectual property will not be cause for exception. Please explain in the manuscript’s Data Availability Statement how readers can access the shared code.

FIGURES AND TABLES

* Please provide titles and legends for all figures and tables (including those in Supporting Information files).

* Please define all abbreviations used in each figure/table (including those in Supporting Information files).

SUPPLEMENTARY MATERIAL

* Please note that supplementary material will be posted as supplied by the authors. Therefore, please amend it according to the relevant comments outlined here.

* Please cite your Supporting Information as outlined here: https://journals.plos.org/plosmedicine/s/supporting-information

OBSERVATIONAL STUDIES

* Please ensure that the study is reported according to the GATHER statement (available from https://www.equator-network.org/reporting-guidelines/gather-statement) and include the completed checklist as Supporting Information. Please add the following statement, or similar, to the Methods: "This study is reported as per the Guidelines for Accurate and Transparent Health Estimates Reporting (GATHER) statement (S1 Checklist)." When completing the checklist, please use section and paragraph numbers, rather than page numbers.

* For all observational studies, in the manuscript text, please indicate: (1) the specific hypotheses you intended to test, (2) the analytical methods by which you planned to test them, (3) the analyses you actually performed, and (4) when reported analyses differ from those that were planned, transparent explanations for differences that affect the reliability of the study's results. If a reported analysis was performed based on an interesting but unanticipated pattern in the data, please be clear that the analysis was data driven.

* Please state in the Methods section whether the study had a prospective protocol or analysis plan. If a prospective analysis plan (from your funding proposal, IRB or other ethics committee submission, study protocol, or other planning document written before analyzing the data) was used in designing the study, please include the relevant document(s) with your revised manuscript as a Supporting Information file to be published alongside your study and cite it in the Methods section. A legend for this file should be included at the end of your manuscript. If no such document exists, please make sure that the Methods section transparently describes when analyses were planned, and when/why any data-driven changes to analyses took place. Changes in the analysis, including those made in response to peer review comments, should be identified as such in the Methods section of the paper, with rationale.

# Author comment: Author response to Decision Letter 3

**DOI:** 10.1371/journal.pmed.1004791.r005

27 May 2026

Attachment Submitted filename: Reviewer PLOS.docx

# Peer review: Decision Letter 3

**DOI:** 10.1371/journal.pmed.1004791.r006

**Authors:** Suzanne De Bruijn

18 Jun 2026

Dear Dr. Bellizzi,

Thank you very much for re-submitting your manuscript "Trends of stillbirth, early neonatal and perinatal mortality rates and sociodemographic differentials of perinatal mortality rates in 17 low- and middle-income countries: an application of modified Gompertz-Makeham model" (PMEDICINE-D-25-03514R3) for review by PLOS Medicine.

I have discussed the paper with my colleagues and the academic editor and it was also seen again by one reviewer. I am pleased to say that provided the remaining editorial and production issues are dealt with we are planning to accept the paper for publication in the journal.

The remaining issues that need to be addressed are listed at the end of this email. Any accompanying reviewer attachments can be seen via the link below. Please take these into account before resubmitting your manuscript:

[LINK]

***Please note while forming your response, if your article is accepted, you may have the opportunity to make the peer review history publicly available. The record will include editor decision letters (with reviews) and your responses to reviewer comments. If eligible, we will contact you to opt in or out.***

In revising the manuscript for further consideration here, please ensure you address the specific points made by each reviewer and the editors. In your rebuttal letter you should indicate your response to the reviewers' and editors' comments and the changes you have made in the manuscript. Please submit a clean version of the paper as the main article file. A version with changes marked must also be uploaded as a marked up manuscript file.

Please also check the guidelines for revised papers at http://journals.plos.org/plosmedicine/s/revising-your-manuscript for any that apply to your paper. If you haven't already, we ask that you provide a short, non-technical Author Summary of your research to make findings accessible to a wide audience that includes both scientists and non-scientists. The Author Summary should immediately follow the Abstract in your revised manuscript. This text is subject to editorial change and should be distinct from the scientific abstract.

In addition to these revisions, you may need to complete some formatting changes, which you will receive in a follow up email. A member of our team will be in touch with a set of requests shortly. If you do not receive a separate email within a few days, please assume that checks have been completed, and no additional changes are required.

We expect to receive your revised manuscript within 1 week. Please email us (plosmedicine@plos.org) if you have any questions or concerns.

We ask every co-author listed on the manuscript to fill in a contributing author statement. If any of the co-authors have not filled in the statement, we will remind them to do so when the paper is revised. If all statements are not completed in a timely fashion this could hold up the re-review process. Should there be a problem getting one of your co-authors to fill in a statement we will be in contact. YOU MUST NOT ADD OR REMOVE AUTHORS UNLESS YOU HAVE ALERTED THE EDITOR HANDLING THE MANUSCRIPT TO THE CHANGE AND THEY SPECIFICALLY HAVE AGREED TO IT.

Please ensure that the paper adheres to the PLOS Data Availability Policy (see http://journals.plos.org/plosmedicine/s/data-availability), which requires that all data underlying the study's findings be provided in a repository or as Supporting Information. For data residing with a third party, authors are required to provide instructions with contact information for obtaining the data. PLOS journals do not allow statements supported by "data not shown" or "unpublished results." For such statements, authors must provide supporting data or cite public sources that include it.

To enhance the reproducibility of your results, we recommend that you deposit your laboratory protocols in protocols.io, where a protocol can be assigned its own identifier (DOI) such that it can be cited independently in the future. Additionally, PLOS ONE offers an option to publish peer-reviewed clinical study protocols. Read more information on sharing protocols at https://plos.org/protocols?utm_medium=editorial-email&utm_source=authorletters&utm_campaign=protocols

Please review your reference list to ensure that it is complete and correct. If you have cited papers that have been retracted, please include the rationale for doing so in the manuscript text, or remove these references and replace them with relevant current references. Any changes to the reference list should be mentioned in the rebuttal letter that accompanies your revised manuscript.

Please note, when your manuscript is accepted, an uncorrected proof of your manuscript will be published online ahead of the final version, unless you've already opted out via the online submission form. If, for any reason, you do not want an earlier version of your manuscript published online or are unsure if you have already indicated as such, please let the journal staff know immediately at plosmedicine@plos.org.

If you have any questions in the meantime, please contact me or the journal staff on plosmedicine@plos.org.

We look forward to receiving the revised manuscript by Jun 25 2026 11:59PM.

Sincerely,

Suzanne De Bruijn, PhD

Senior Editor

PLOS Medicine

plosmedicine.org

------------------------------------------------------------

Requests from Editors:

Line edits:

Line 38: unclear what is meant by ‘heaping’. Please clarify for a general reader.

Line 51: Please temper claim of ‘robust modality’ and reframe along the lines of “Adjusting for data quality issues can potentially improve the use of….”

line 121: insert comma after 'neonatal mortality data'

The first paragraph of the Results section is not broadly accessible to a general reader. Please revise.

Please use the active voice.

Here and elsewhere, please explain how the statistical adjustment altered estimates or trends.

line 283-284: Please clarify for general reader "The heaping index and the D0-1/D2-6

disaggregated by covariates "

line 287: 'of the' is repeated twice

Line 306: please replace 'strong' with a more objective descriptor

line 426: replace 'politically' with 'political'

Line 472: revise to “Mohamed M Ali is a former WHO staff member and currently a freelance…”

Title of S2 Table: should this be Excluded surveys (plural)?

Figure 1: please revise to Figure 1a, 1b and 1c or to Figures 1, 2, 3. Please change the figure callouts accordingly.

Please justify the choice of the 17 countries assessed.

Please provide greater discussion of the 'sociodemographic differentials' in the Results and Discussion or remove from the title.

* Please confirm that your title complies with PLOS Medicine's style. Your title must be nondeclarative and not a question. It should begin with main concept if possible. "Effect of" should be used only if causality can be inferred, i.e., for an RCT. Please place the study design ("A randomized controlled trial," "A retrospective study," "A modelling study," etc.) in the subtitle (ie, after a colon). . Consider: “Trends of stillbirth, early neonatal and perinatal mortality rates and socioeconomic inequalities in 17 low- and middle-income countries: a modeling study” or revise to better reflect the novel aspects of the study.

* Please confirm that your abstract complies with our requirements, including format (three sections: Background, Methods and Findings, and Conclusions) and providing all the information relevant to this study type https://journals.plos.org/plosmedicine/s/submission-guidelines#loc-abstract

* Please ensure that the Introduction ends with a clear description of the study question or hypothesis.

* Please ensure that all abbreviations are defined at first use throughout the text.

* Please confirm that all numbers presented in the abstract are present and identical to numbers presented in the main manuscript text.

GENERAL

* Please disclose any use of AI tools and technologies to the study or article content at the end of the Methods. Please specify the name(s) and version(s) of any tool(s) used and a description of their use, including how the AI-assisted or generated content was evaluated by authors, and a clear statement of which aspects of the study, article contents, data, or supporting files were affected/generated by AI tool usage. Please also include a statement specifying who retains responsibility for the final manuscript. See our guidelines for more information: https://journals.plos.org/plosmedicine/s/ethical-publishing-practice#loc-artificial-intelligence-tools-and-technologies.

* Statistical reporting: Please revise throughout the manuscript, including tables and figures.

- Please report statistical information as follows to improve clarity for the reader "22% (95% CI [13,28]; p</=)".

- Please separate upper and lower bounds with commas instead of hyphens as the latter can be confused with reporting of negative values.

- Please repeat statistical definitions (HR, CI etc.) for each set of parentheses.

Author summary

* In the author summary, in the final bullet point of 'What Do These Findings Mean?', please include the main limitations of the study in non-technical language.

* Please mention the sociodemographic analyses in the section “What did the researchers do and find?’

FUNDING STATEMENT

Please explain to editors why there is no funder acknowledged. And if there was no specific funding for the study, please change author(s) to authors in the financial disclosure.

COMPETING INTERESTS STATEMENT

* All authors must declare their relevant competing interests per the PLOS policy, which can be seen here: https://journals.plos.org/plosmedicine/s/competing-interests For authors with ties to industry, please indicate whether any of the interests has a financial stake in the results of the current study.

DATA AVAILABILITY

* PLOS defines the “minimal data set” to consist of the data set used to reach the conclusions drawn in the manuscript with related metadata and methods, and any additional data required to replicate the reported study findings in their entirety. Authors do not need to submit their entire data set, or the raw data collected during an investigation. Please submit the following data:

The values behind the means, standard deviations and other measures reported;

The values used to build graphs;

The points extracted from images for analysis.

* The Data Availability Statement (DAS) requires revision. For each data source used in your study:

a) If the data are freely or publicly available, note this and state the location of the data: within the paper, in Supporting Information files, or in a public repository (include the DOI or accession number).

b) If the data are owned by a third party but freely available upon request, please note this and state the owner of the data set and contact information for data requests (web or email address). Note that a study author cannot be the contact person for the data.

c) If the data are not freely available, please describe briefly the ethical, legal, or contractual restriction that prevents you from sharing it. Please also include an appropriate contact (web or email address) for inquiries (again, this cannot be a study author).

* For studies in which a novel model is central to the manuscript's findings, authors are responsible for providing the source code needed to replicate the study's findings in a repository (such as GitHub, SourceForge or Bitbucket) or a cloud computing service (such as Code Ocean). Please include a citable digital object identifier (DOI) or other persistent identifier of the code (such as through linkage with Zenodo). Protection of authors’ intellectual property will not be cause for exception. Please explain in the manuscript’s Data Availability Statement how readers can access the shared code. Please provide both the Github link and a DOI.

FIGURES

* Please provide titles and legends for all figures and tables (including those in Supporting Information files). Please define all acronyms used in each figure or table in its corresponding legends

* Please provide the unadjusted comparisons as well as the adjusted comparisons in all relevant Tables

* Please specify the variables controlled for in all relevant Tables

INCLUSIVITY IN GLOBAL RESEARCH

PLOS has a 'Inclusivity in Global Research' policy which aims to promote collaboration and inclusivity in global health research. You are required to complete PLOS’ questionnaire on inclusivity in global research and submit it with your revised paper. The policy and questionnaire can be found at https://journals.plos.org/plosone/s/best-practices-in-research-reporting.

MODELING STUDIES

For more details on the requests below please see Geoffrey P Garnett, Simon Cousens, Timothy B Hallett, Richard Steketee, Neff Walker. Mathematical models in the evaluation of health programmes. (2011) Lancet DOI:10.1016/S0140-6736(10)61505-X.

* Please provide a diagram that shows the model structure, including how the disease natural history is represented, the process and determinants of disease acquisition, and how the putative intervention could affect the system.

* Please provide a complete list of model parameters, including clear and precise descriptions of the meaning of each parameter, together with the values or ranges for each, with justification or the primary source cited, and important caveats about the use of these values noted.

* Please provide a clear statement about how the model was fitted to the data including where relevant goodness-of-fit measure, the numerical algorithm used, which parameter varied, constraints imposed on parameter values, and starting conditions.

* For uncertainty analyses, please state the sources of uncertainties quantified and not quantified this can include parameter, data, and model structure.

* Please provide sensitivity analyses to identify which parameter values are most important in the model. Uncertainty estimates seek to derive a range of credible results on the basis of an exploration of the range of reasonable parameter values. The choice of method should be presented and justified.

* Please discuss the scientific rationale for this choice of model structure and identify points where this choice could influence conclusions drawn. Please also describe the strength of the scientific basis underlying the key model assumptions.

Comments from Reviewers:

Reviewer #1: No further queries

Any attachments provided with reviews can be seen via the following link:

[LINK]

# Author comment: Author response to Decision Letter 4

**DOI:** 10.1371/journal.pmed.1004791.r007

3 Jul 2026

Attachment Submitted filename: PLOS_Medicine_Response_to_Editors.docx

# Editor report: Decision Letter 4

**DOI:** 10.1371/journal.pmed.1004791.r008

**Authors:** Suzanne De Bruijn

6 Jul 2026

Dear Dr. Bellizzi,

Thank you very much for re-submitting your manuscript "Trends of stillbirth, early neonatal and perinatal mortality rates and sociodemographic differentials of perinatal mortality rates in 17 low- and middle-income countries: an application of modified Gompertz-Makeham model" (PMEDICINE-D-25-03514R4) for review by PLOS Medicine.

Thank you for addressing our editorial requests. However, there are several remaining issues, which I have listed at the bottom of this email. Can you please address these issues, and provide a point-by-point response to each of these requests? Please do this in the form of 'our request' then 'your reply'.

In general, we would ask you to expand your results section to provide more clarity for the readers. I appreciate you provide a wealth of data, which can all be found in the tables and figures, but we would appreciate more descriptions and more numerical results in the results section for clarity.

***Please note while forming your response, if your article is accepted, you may have the opportunity to make the peer review history publicly available. The record will include editor decision letters (with reviews) and your responses to reviewer comments. If eligible, we will contact you to opt in or out.***

In addition to these revisions, you may need to complete some formatting changes, which you will receive in a follow up email. A member of our team will be in touch with a set of requests shortly. If you do not receive a separate email within a few days, please assume that checks have been completed, and no additional changes are required.

We expect to receive your revised manuscript within 1 week. Please email us (plosmedicine@plos.org) if you have any questions or concerns.

We ask every co-author listed on the manuscript to fill in a contributing author statement. If any of the co-authors have not filled in the statement, we will remind them to do so when the paper is revised. If all statements are not completed in a timely fashion this could hold up the re-review process. Should there be a problem getting one of your co-authors to fill in a statement we will be in contact. YOU MUST NOT ADD OR REMOVE AUTHORS UNLESS YOU HAVE ALERTED THE EDITOR HANDLING THE MANUSCRIPT TO THE CHANGE AND THEY SPECIFICALLY HAVE AGREED TO IT.

Please note, when your manuscript is accepted, an uncorrected proof of your manuscript will be published online ahead of the final version, unless you've already opted out via the online submission form. If, for any reason, you do not want an earlier version of your manuscript published online or are unsure if you have already indicated as such, please let the journal staff know immediately at plosmedicine@plos.org.

If you have any questions in the meantime, please contact me or the journal staff on plosmedicine@plos.org.

We look forward to receiving the revised manuscript by Jul 13 2026 11:59PM.

Sincerely,

Suzanne De Bruijn, PhD

Associate Editor

PLOS Medicine

plosmedicine.org

------------------------------------------------------------

Requests from Editors:

GENERAL EDITORIAL REQUESTS

1) Please confirm that your title complies with PLOS Medicine's style. Your title must be nondeclarative and not a question. It should begin with main concept if possible. "Effect of" should be used only if causality can be inferred, i.e., for an RCT. Please place the study design ("A randomized controlled trial," "A retrospective study," "A modelling study," etc.) in the subtitle (ie, after a colon).

* Please modify your title to the following: "Trends of stillbirth, early neonatal and perinatal mortality rates and sociodemographic differentials of perinatal mortality rates in 17 low- and middle-income countries: A modeling study"

2) Please disclose any use of AI tools and technologies to the study or article content at the end of the Methods. Please specify the name(s) and version(s) of any tool(s) used and a description of their use, including how the AI-assisted or generated content was evaluated by authors, and a clear statement of which aspects of the study, article contents, data, or supporting files were affected/generated by AI tool usage. Please also include a statement specifying who retains responsibility for the final manuscript. See our guidelines for more information: https://journals.plos.org/plosmedicine/s/ethical-publishing-practice#loc-artificial-intelligence-tools-and-technologies.

* Please either provide a statement in your methods section, or provide a statement in the point-by-point response letter.

3) Statistical reporting: Please revise throughout the manuscript, including tables and figures.

- Please report statistical information as follows to improve clarity for the reader "22% (95% CI [13,28]; p</=)".

- Please separate upper and lower bounds with commas instead of hyphens as the latter can be confused with reporting of negative values.

- Please repeat statistical definitions (HR, CI etc.) for each set of parentheses.

* I appreciate you have reformatted this in most instances, but not everywhere (e.g. line 375). Please check the whole manuscript and adjust where necessary.

4) In the last sentence of the Abstract Methods and Findings section, please describe the main limitation(s) of the study's methodology. I appreciate that you included the limitations of the data, but please also include the limitations of the methodology.

5) In the abstract, please include the important dependent variables that are adjusted for in the analyses.

6) Author summary: Please mention the sociodemographic analyses in the section “What did the researchers do and find?’

7) Author summary: what did the researchers do and find: please consider removing ‘age-at-death heaping’ as this is not a general term for a laymens audience.

8) Author summary: 'what did the researchers do and find', first point: Please change this statement, as your study provides the adjusted estimates and stillbirth rates. Unless I am wrong, you don't provide evidence that these are improved compared to the raw DHS data.

9) In the author summary, in the final bullet point of 'What Do These Findings Mean?', please include the main limitations of the study in non-technical language.

FUNDING STATEMENT

10) Please change 'author(s)' to 'authors' in the funding statement.

DATA AVAILABILITY

11) PLOS defines the “minimal data set” to consist of the data set used to reach the conclusions drawn in the manuscript with related metadata and methods, and any additional data required to replicate the reported study findings in their entirety. Authors do not need to submit their entire data set, or the raw data collected during an investigation. Please submit the following data:

The values behind the means, standard deviations and other measures reported;

The values used to build graphs;

* I appreciate your reply that these will be provided, but we do need these data before we can proceed with the paper. Please include the data now in a supplemental file. Please also provide a title and legend for this file. Finally, please mention this supplemental file in the legend of the corresponding figures.

12) Please update your data availability statement.

-Please clarify which data is available instead of 'all data'; e.g. DHS surveys

-thank you for including the URL

-you state that the data is available 'upon request'. Please clarify whether these data are freely available, or whether there are restrictions. Please also mention what authors have to do to request the data.

* PLOS Medicine requires that the de-identified data underlying the specific results in a published article be made available, without restrictions on access, in a public repository or as Supporting Information at the time of article publication, provided it is legal and ethical to do so. Please see the policy at http://journals.plos.org/plosmedicine/s/data-availability and FAQs at http://journals.plos.org/plosmedicine/s/data-availability#loc-faqs-for-data-policy

CODE AVAILABILITY

13) Please include the statement on code availability in the data availability statement. Please see guidance here:https://journals.plos.org/plosmedicine/s/code-sharing-guidance#loc-data-availability-statement-examples

* I appreciate your response here that "The statistical code will be deposited in a public repository and cited through a persistent identifier in the revised submission.” However, we need this statement, as well as the DOI, before we can proceed with your manuscript.

INCLUSIVITY IN GLOBAL RESEARCH

PLOS has a 'Inclusivity in Global Research' policy which aims to promote collaboration and inclusivity in global health research. You are required to complete PLOS’ questionnaire on inclusivity in global research and submit it with your revised paper. The policy and questionnaire can be found at https://journals.plos.org/plosone/s/best-practices-in-research-reporting.

14) Please confirm whether you have provided us with this questionnaire.

Furthermore, we have several additional editorial requests:

-Please justify the choice of the 17 countries assessed. Please provide clarification in the introduction, as well as the methods and the results. If I understand correctly, you looked at LMIC with enough DHS surveys, but this is not at all clear from the manuscript itself.

-Please explain how the statistical adjustments through the modified Gompertz-Makeham model altered estimates or trends: You currently provide the modified estimated and the trends, but you don't provide how the model changes these estimates.

-Line 93-95: “Despite the enormous global significance of stillbirths and early neonatal mortality, lack of high-quality data and application of appropriate analytical tools limited the usefulness of such data.” Please consider rephrasing, as it currently states that lack of data limits the usefulness of the data.

-Results, first paragraph: Please consider including the number of countries and a description of what type of countries (e.g: LMIC? Africa? Africa/asia/south-america?, 4 regions?), and the number of surveys that were included. In general, please provide more information in this paragraph in order to provide additional clarity for the reader.

-Results, first paragraph:Please explain the heaping index.

-Results, first paragraph:Please provide the explanation for the D0-1 and D2-6 measures in the results section for clarity for the reader.

-line 296: what do you mean ‘significantly adjusted’?

-line 296-303: Please include more of the numerical results in the paragraph.

-line 295-299: Please consider splitting this sentence in two for clarity.

-line 304: Please clarify what the sensitivity analysis entailed.

-line 324-325: consider writing “annual changes were generally more pronounced for early neonatal mortality than for stillbirths, which affected the overall perinatal mortality (numbers?)

-Line 391: please change ‘weak health system’ to ‘weak health systems’ (plural)

-Line 406: please remove ‘indeed’.

-Line 412: ‘survey value’ what do you mean here, please clarify. Also consider whether ‘value’ needs to be plural.

-Line 422: please change ‘political’ to ‘politics’

Table 1: am I correct that these are the data from the Survey (not-adjusted)?

-Please provide a clear title (please remove ‘95% CI from the title), and provide a legend with additional information. Please also consider including the years of first-last surveys in this table, rather than in a supplemental table.

-Table 2: Please provide a legend with more information (e.g.: this is pooled data from all 17 countries?), and ensure you clarify all abbreviations (including mGM). Please also include covariates these aRRs are adjusted for in the legend.

Figures 1-3: Please provide legends for these figures.

# Author comment: Author response to Decision Letter 5

**DOI:** 10.1371/journal.pmed.1004791.r009

27 Jul 2026

Attachment Submitted filename: PLOS_Response_to_Editor.docx

# Editor report: Decision Letter 5

**DOI:** 10.1371/journal.pmed.1004791.r010

**Authors:** Suzanne De Bruijn

30 Jul 2026

Dear Dr Bellizzi,

On behalf of my colleagues and the Academic Editor, Margaret Kruk, I am pleased to inform you that we have agreed to publish your manuscript "Trends of stillbirth, early neonatal and perinatal mortality rates and sociodemographic differentials of perinatal mortality rates in 17 low- and middle-income countries:  A modeling study" (PMEDICINE-D-25-03514R5) in PLOS Medicine.

Before your manuscript can be formally accepted you will need to complete some formatting changes, which you will receive in a follow up email. Please be aware that it may take several days for you to receive this email; during this time no action is required by you. Once you have received these formatting requests, please note that your manuscript will not be scheduled for publication until you have made the required changes.

In the meantime, please log into Editorial Manager at http://www.editorialmanager.com/pmedicine/, click the "Update My Information" link at the top of the page, and update your user information to ensure an efficient production process.

PRESS

We frequently collaborate with press offices. If your institution or institutions have a press office, please notify them about your upcoming paper at this point, to enable them to help maximise its impact. If the press office is planning to promote your findings, we would be grateful if they could coordinate with medicinepress@plos.org. If you have not yet opted out of the early version process, we ask that you notify us immediately of any press plans so that we may do so on your behalf.

We also ask that you take this opportunity to read our Embargo Policy regarding the discussion, promotion and media coverage of work that is yet to be published by PLOS. As your manuscript is not yet published, it is bound by the conditions of our Embargo Policy. Please be aware that this policy is in place both to ensure that any press coverage of your article is fully substantiated and to provide a direct link between such coverage and the published work. For full details of our Embargo Policy, please visit http://www.plos.org/about/media-inquiries/embargo-policy/.

Thank you again for submitting to PLOS Medicine. We look forward to publishing your paper.

Sincerely,

Suzanne De Bruijn, PhD

Associate Editor

PLOS Medicine
